# Transcriptional signatures of BALB/c mouse macrophages housing multiplying *Leishmania amazonensis *amastigotes

**DOI:** 10.1186/1471-2164-10-119

**Published:** 2009-03-20

**Authors:** José Osorio y Fortéa, Emilie de La Llave, Béatrice Regnault, Jean-Yves Coppée, Geneviève Milon, Thierry Lang, Eric Prina

**Affiliations:** 1 Institut Pasteur, Unité d'Immunophysiologie et Parasitisme Intracellulaire, Département de Parasitologie et Mycologie, 25 rue du Docteur Roux, 75724 Paris, France; 2 Institut Pasteur, Génopole Plate-Forme 2, Puces à ADN, 28 rue du Docteur Roux, 75724 Paris, France

## Abstract

**Background:**

Mammal macrophages (MΦ) display a wide range of functions which contribute to surveying and maintaining tissue integrity. One such function is phagocytosis, a process known to be subverted by parasites like *Leishmania (L)*. Indeed, the intracellular development of *L. amazonensis *amastigote relies on the biogenesis and dynamic remodelling of a phagolysosome, termed the parasitophorous vacuole, primarily within dermal MΦ.

**Results:**

Using BALB/c mouse bone marrow-derived MΦ loaded or not with amastigotes, we analyzed the transcriptional signatures of MΦ 24 h later, when the amastigote population was growing. Total RNA from MΦ cultures were processed and hybridized onto Affymetrix Mouse430_2 GeneChips^®^, and some transcripts were also analyzed by Real-Time quantitative PCR (RTQPCR). A total of 1,248 probe-sets showed significant differential expression. Comparable fold-change values were obtained between the Affymetrix technology and the RTQPCR method. Ingenuity Pathway Analysis software^® ^pinpointed the up-regulation of the sterol biosynthesis pathway (p-value = 1.31e-02) involving several genes (1.95 to 4.30 fold change values), and the modulation of various genes involved in polyamine synthesis and in pro/counter-inflammatory signalling.

**Conclusion:**

Our findings suggest that the amastigote growth relies on early coordinated gene expression of the MΦ lipid and polyamine pathways. Moreover, these MΦ hosting multiplying *L. amazonensis *amastigotes display a transcriptional profile biased towards parasite-and host tissue-protective processes.

## Background

*L. amazonensis *are protozoan parasites belonging to the trypanosomatidae family. In natural settings, the *L. amazonensis *perpetuation relies on blood-feeding sand fly and rodent hosts. The development of promastigotes proceeds within the gut lumen of the sand fly hosts and ends with metacyclic promastigotes. The latter, once delivered into the mammal dermis, differentiate as amastigotes mainly within the resident dermal macrophage (MΦ) acting as *bona fide *host cells. Following the parasite inoculation and before the development of the more or less transient skin damages that characterize cutaneous leishmaniasis there is an asymptomatic phase lasting for several days or weeks during which the intracellular amastigote progeny expands. This expansion takes place within a compartment named parasitophorous vacuole (PV) that displays properties similar to late endosomes/lysosomes and the size of which grows significantly for *Leishmania *belonging to the *mexicana *complex [1,2]. In this study we sought to analyze the transcriptional signatures of a homogeneous population of MΦ derived *in vitro *from BALB/c mouse bone marrow CSF-1 dependent progenitors and hosting amastigotes that are actively multiplying. The Affymetrix GeneChip technology was used to compare the gene expression profiles of *L. amazonensis *amastigotes-hosting bone marrow-derived MΦ and parasite-free ones. This *in vitro *transcriptomics approach was combined with the Ingenuity biological network analysis to highlight the mouse MΦ biological processes the multiplying *L. amazonensis *amastigotes rely on within their giant communal PV. Our findings suggest that MΦ hosting multiplying amastigotes contribute to carve a parasite-as well as a host tissue-protective environment.

## Results and Discussion

**Figure 1:**
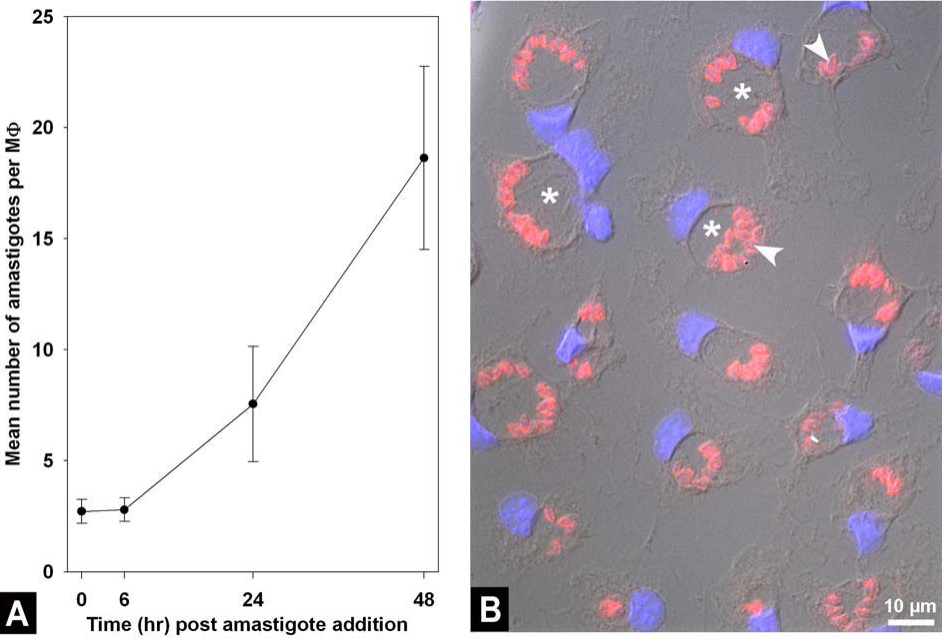
**Time course of intracellular amastigote population size increase and MΦ culture imaging**. **A**: time course experiment showing the evolution of the amastigote population within MΦ. Mean number of amastigotes per MΦ were plotted against the time points selected. Ten microscope fields split up into biological duplicates were visualized and more than 200 MΦ nuclei were counted. **B**: *L. amazonensis*-housing bone marrow-derived MΦ imaged 24 h post amastigote (4 parasites per MΦ) addition. Nuclei were stained with Hoechst (blue) and amastigote with 2A3.26 mAb and Texas Red-labelled conjugate (red). Image acquisition was performed using an immunofluorescence and differential interference contrast inverted microscope (Zeiss Axiovert 200 M). Asterisk: Parasitophorous vacuoles; arrow heads: Amastigotes.

**Figure 2:**
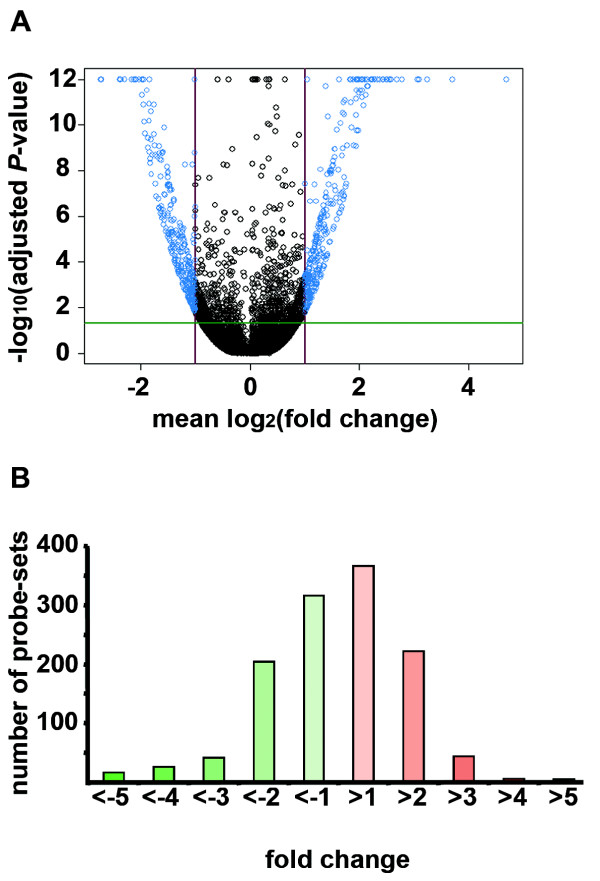
**Affymetrix outcome**. **A**: Volcano plot. 1,248 probe-sets showed differential expression at the 0.05 threshold (green line): 605 positive and 643 negative FC values of which 454 in the right and 507 in the left upper corners (± 1.75 FC threshold, red lines, blue circles). **B**: Fold-change distribution of the 1,248 probe sets.

*L. amazonensis *amastigotes subvert MΦ as host cells where they enter a cell-cycling phase lasting several days (Fig. 1A). We compared the transcriptomes of amastigote-free MΦ and amastigote-harbouring MΦ 24 h after the uptake of amastigotes carefully purified from nude mouse lesions. At this time-point amastigotes were multiplying within a huge PV (Fig. 1B) and their population size had almost doubled (Fig. 1A). Among the 45,101 probe-sets of the Mouse430_2 GeneChip, 1,248 (2.77%) were displaying features of differential expression at the 5% significance level (Fig. 2, see Additional file 1). Of these, 1,206 matched Ingenuity Pathway Analysis database version 5.5.1 which represented 898 genes with a known function. About 80% of these genes were incorporated into either Ingenuity's canonical pathway or biological network (*i.e. *their products interact with other molecules in Ingenuity's knowledge base). The symbols of the modulated genes are specified in the text (fold change [FC] values between brackets), while their full names are given in Additional file 1. Furthermore, comparable FC values were obtained between the Affymetrix technology and the Real Time quantitative Polymerase Chain Reaction (RTQPCR) method (Table 1) [3].

**Table 1:** List of differentially expressed genes between *L. amazonensis*-harbouring MΦ and parasite-free MΦ.

**Symbol**	**Name**	**Probe-set**	**LocusLink**	**Affymetrix (RTqPCR)**	**P-value**
***abcD2***	ATP-binding cassette, sub-family D (ALD), member 2	1438431_at^a^	26874	-2.11	4.40e-03
***acaca***	acetyl-Coenzyme A carboxylase alpha	1427595_at	107476	-1.32	4.79e-03
***acsl3***	acyl-CoA synthetase long-chain family member 3	1452771_s_at	74205	+2.09	1.48e-03
***adhfe1***	alcohol dehydrogenase, iron containing, 1	1424393_s_at	76187	+1.61	4.40e-02
***akr1a1***	aldo-keto reductase family 1, member A1 (aldehyde reductase)	1430123_a_at	58810	+1.13	1.22e-03
***aldoA***	aldolase 1, A isoform	1433604_x_at^a^	11674	+1.72	1.28e-02
***aldoC***	aldolase 3, C isoform	1451461_a_at	11676	+1.89	1.13e-02
***anxA1***	annexin A1	1444016_at^a^	16952	+2.68	4.47e-05
***apoc2***	apolipoprotein C-II	1418069_at	11813	-1.63	4.57e-02
***arg2***	Arginase 2	1418847_at	11847	NM (+1.91)	NS
***atf1***	activating transcription factor 1	1417296_at	11908	+1.84	4.20e-03
***atf3***	activating transcription factor 3	1449363_at	11910	+1.77	1.09e-02
***atp6V0a1***	ATPase, H+ transporting, lysosomal V0 subunit a isoform 1	1460650_at^a^	11975	+1.82	8.31e-03
***atp6V0c***	ATPase, H+ transporting, V0 subunit C	1435732_x_at	11984	+1.27	5.48e-13
***atp6V0d2***	ATPase, H+ transporting, V0 subunit D, isoform 2	1444176_at^a^	24234	+2.32	1.12e-05
***atp6V1a***	ATPase, H+ transporting, V1 subunit A1	1422508_at	11964	+1.57	3.96e-02
***atp6V1c1***	ATPase, H+ transporting, V1 subunit C, isoform 1	1419546_at^a^	66335	+2.31	1.10e-05
***atp6V1d***	ATPase, H+ transporting, V1 subunit D	1416952_at^a^	73834	+1.82	6.97e-03
***atp6V1g1***	ATPase, H+ transporting, V1 subunit G isoform 1	1423255_at^a^	66290	+1.84	3.78e-03
***atp6V1h***	ATPase, H+ transporting, lysosomal, V1 subunit H	1415826_at	108664	+1.69	2.39e-02
***azin1***	antizyme inhibitor 1	1422702_at	54375	+1.96	1.46e-03
***brd8***	bromodomain containing 8	1427193_at	78656	+1.08	3.75e-02
***c1qa***	complement component 1, q subcomponent, alpha polypeptide	1417381_at	12259	-1.48	3.15e-02
***c1qb***	complement component 1, q subcomponent, beta polypeptide	1417063_at	12260	-1.77	3.31e-04
***c3***	complement component 3	1423954_at	12266	-2.37	7.05e-06
***c4b***	complement component 4 (within H-2S)	1418021_at	12268	-1.76	4.55e-02
***c5ar1***	complement component 5a receptor 1	1439902_at	247623	-1.63	4.62e-02
***ccr2***	chemokine (C-C motif) receptor 2	1421187_at^a^	12772	-1.83 (-2.35)	6.42e-03
***ccr3***	chemokine (C-C motif) receptor 3	1422957_at	12771	-2.58 (-3.88)	2.49e-05
***cd14***	CD14 antigen	1417268_at	12475	-1.73	1.54e-03
***cd200***	CD200 antigen	1448788_at	17470	+4.14 (+6.52)	5.48e-13
***cd274***	CD274 antigen	1419714_at	60533	+1.93	1.61e-03
***cd86***	CD86 antigen	1420404_at^a^	12524	-1.83 (-1.03)	1.44e-02
***cfh***	complement component factor h	1450876_at	12628	-2.80	6.08e-06
***c-fos***	FBJ osteosarcoma oncogene	1423100_at	14281	-1.93	3.30e-03
***ch25h***	cholesterol 25-hydroxylase	1449227_at	12642	-6.57	1.39e-22
***cmklr1***	chemokine-like receptor 1	1456887_at	14747	-2.20	1.57e-04
***cx3cr1***	chemokine (C-X3-C) receptor 1	1450020_at	13051	-2.65 (-5.26)	2.39e-05
***cyp51***	cytochrome P450, family 51	1450646_at^a^	13121	+2.78	2.10e-07
***dhcr24***	24-dehydrocholesterol reductase	1451895_a_at	74754	+3.17	2.69e-09
***dio2***	deiodinase, iodothyronine, type II	1418937_at^a^	13371	+25.92 (+41.03)	0.00e+00
***eno2***	enolase 2, gamma neuronal	1418829_a_at	13807	+2.60	6.08e-06
***fabp3***	fatty acid binding protein 3	1416023_at	14077	+2.29	5.58e-05
***fabp4***	fatty acid binding protein 4	1417023_a_at^a^	11770	+6.42	0.00e+00
***fabp5***	fatty acid binding protein 5	1416022_at^a^	16592	+1.57	4.70e-08
***fbp1***	fructose bisphosphatase 1	1448470_at	14121	-2.16	4.68e-03
***fdft1***	farnesyl diphosphate farnesyl transferase 1	1438322_x_at^a^	14137	+2.62	4.00e-06
***fdps***	farnesyl diphosphate synthetase	1423418_at	110196	+3.59	9.78e-12
***h-2ma***	histocompatibility 2, class II, locus DMa	1422527_at	14998	-1.88	3.00e-03
***h60***	histocompatibility 60	1439343_at	15101	-2.07	5.30e-09
***hk2***	hexokinase 2	1422612_at	15277	+1.75	1.09e-02
***hk3***	hexokinase 3	1435490_at	212032	+2.03	3.72e-04
***hmgcr***	3-hydroxy-3-methylglutaryl-Coenzyme A reductase	1427229_at	15357	+1.95	2.34e-03
***hmgcs1***	3-hydroxy-3-methylglutaryl-Coenzyme A synthase 1	1433446_at	208715	+2.48	1.07e-06
***hsd17b7***	hydroxysteroid (17-beta) dehydrogenase 7	1457248_x_at	15490	+2.73	1.41e-05
***icam1***	intercellular adhesion molecule	1424067_at	15894	-1.75	1.43e-02
***icam2***	intercellular adhesion molecule 2	1448862_at	15896	-1.85	2.88e-02
***idi1***	isopentenyl-diphosphate delta isomerase	1451122_at^a^	319554	+2.72	2.77e-07
***ifngr1***	interferon gamma receptor 1	1448167_at	15979	-1.83 (-2.16)	4.66e-03
***il10***	interleukin 10	1450330_at	16153	-2.97 (-4.46)	1.11e-07
***il10ra***	interleukin 10 receptor, alpha	1448731_at	16154	-2.16 (-2.56)	4.40e-04
***il11ra1***	interleukin 11 receptor, alpha chain 1	1417505_s_at	16157	+2.24 (+3.55)	9.89e-05
***il17rb***	interleukin 17 receptor B	1420678_a_at	50905	-1.41	2.93e-02
***il18***	interleukin 18	1417932_at	16173	-1.77 (-2.12)	1.06e-02
***il1b***	interleukin 1 beta	1449399_a_at	16176	-3.09 (-5.17)	3.49e-07
***il1rn***	interleukin 1 receptor antagonist	1423017_a_at^a^	16181	+4.19 (+7.86)	0.00e+00
***insig1***	insulin induced gene 1	1454671_at	231070	+2.62	9.17e-08
***itga4***	integrin alpha 4	1456498_at^a^	16401	-2.06	2.37e-03
***itgal***	integrin alpha L	1435560_at	16408	-2.00	7.72e-03
***klrk1***	killer cell lectin-like receptor subfamily K, member 1	1450495_a_at	27007	-1.72	2.21e-02
***ldhA***	lactate dehydrogenase 1, A chain	1419737_a_at	16828	+1.79	2.71e-04
***ldlr***	low density lipoprotein receptor	1450383_at^a^	16835	+4.68	1.49e-13
***lipe***	lipase, hormone sensitive	1422820_at	16890	-2.20	2.90e-03
***lpl***	lipoprotein lipase	1431056_a_at	16956	-1.44	3.24e-02
***lss***	lanosterol synthase	1420013_s_at	16987	+2.05	2.29e-03
***maoa***	monoamine oxidase A	1428667_at^a^	17161	+2.56	4.71e-06
***mapk14***	mitogen activated protein kinase 14 (p38 mapk)	1416703_at	26416	-1.61	4.97e-02
***mgll***	monoglyceride lipase	1426785_s_at	23945	+3.40	3.75e-08
***mvd***	mevalonate (diphospho) decarboxylase	1417303_at^a^	192156	+2.15	6.33e-04
***ncoa4***	nuclear receptor coactivator 4	1450006_at	27057	+1.65	3.15e-02
***nfkbia***	nuclear factor of kappa light chain gene enhancer in B-cells inhibitor, alpha	1448306_at	18035	-1.83	5.53e-03
***nos2***	nitric oxide synthase 2, inducible, macrophage	1420393_at	18126	NM (+1.28)	NS
***odc1***	Ornithine decarboxylase 1	1427364_a_at	18263	NM (+1.18)	NS
***p4ha2***	procollagen-proline, 2-oxoglutarate 4-dioxygenase (proline 4-hydroxylase), α II polypeptide	1417149_at	18452	+2.27	1.96e-03
***pfkl***	phosphofructokinase, liver, B-type	1439148_a_at	18641	+1.68	2.32e-02
***pkg1***	phosphoglycerate kinase 1	1417864_at	18655	+1.70	8.88e-03
***pkm2***	pyruvate kinase, muscle	1417308_at	18746	+1.51	4.57e-02
***ppap2B***	phosphatidic acid phosphatase type 2B	1448908_at^a^	67916	+8.53	0.00e+00
***pros1***	protein S (alpha)	1426246_at	19128	-2.09	2.66e-03
***relb***	avian reticuloendotheliosis viral (v-rel) oncogene related B	1417856_at	19698	-1.91	1.94e-02
***sat1***	spermidine/spermine N1-acetyl transferase 1	1420502_at	20229	+1.47	2.30e-02
***sc4mol***	sterol-C4-methyl oxidase-like	1423078_a_at	66234	+2.28	1.61e-05
***sc5d***	sterol-C5-desaturase (fungal ERG3, delta-5-desaturase) homolog (S. cerevisae)	1451457_at^a^	235293	+2.57	2.37e-06
***scd1***	stearoyl-Coenzyme A desaturase 1	1415964_at^a^	20249	+2.68	4.50e-05
***scd2***	stearoyl-Coenzyme A desaturase 2	1415824_at^a^	20250	+2.45	1.32e-06
***serping1***	serine (or cysteine) peptidase inhibitor, clade G, member 1	1416625_at	12258	-1.35	4.84e-05
***slc7a2***	solute carrier family 7 (cationic amino acid transporter, y+ system), member 2	1436555_at^a^	11988	+4.14	6.07e-12
***sms***	spermine synthase	1434190_at^a^	20603	NM [-1.38^b^]	NS
***socs6***	suppressor of cytokine signaling 6	1450129_a_at	54607	+1.84	4.18e-03
***sqle***	squalene epoxidase	1415993_at	20775	+4.30	0.00e+00
***srebf2***	sterol regulatory element binding factor 2	1426744_at	20788	+1.84	1.26e-02
***srm***	spermidine synthase	1421260_a_at	20810	NM [-1.22^b^]	NS
***stard4***	StAR-related lipid transfer (START) domain containing 4	1429239_a_at^a^	170459	+2.31	2.43e-04
***tlr2***	toll-like receptor 2	1419132_at	24088	-3.11 (-1.58)	1.83e-08
***tlr7***	toll-like receptor 7	1449640_at	170743	-1.77 (-1.07)	4.61e-02
***tlr8***	toll-like receptor 8	1450267_at	170744	-1.79	1.00e-02
***tollip***	toll interacting protein	1423048_a_at	54473	+1.69	3.57e-02

This table is an excerpt from the table of the 1,248 significantly modulated probe-sets, available as online Additional file 1, and contains some genes tested by RTQPCR.

^a ^when several probe-sets detect a target gene, data are only shown for the most modulated one. NM: No Modulation significantly detected with Affymetrix technology. NS: Not Significant *p*-value. ^b ^mean values obtained from the raw fluorescence intensities.

Though transcriptional changes due to the phagocytic uptake process *per se *– known to occur mostly within the first 2 hours post particle addition – cannot be completely excluded, the MΦ transcript modulation – detected at 24 h post the amastigote addition – very likely reflects MΦ reprogramming due to the presence of cell cycling amastigotes within giant PV. Indeed, in our experimental conditions, no extracellular amastigotes could be evidenced in the MΦ culture (a) after a brief centrifugation step and (b) one hour contact with adherent MΦ indicating that the phagocytic uptake of *L. amazonensis *amastigotes is a rapid and efficient process. Furthermore, it is worth mentioning that the size of the amastigote population hosted within the MΦ PV rapidly expands within the first 24 h (Fig. 1A) [4]. Using also mouse bone marrow-derived MΦ as host cells for *Leishmania*, Gregory and coworkers demonstrated that the gene expression profiles of control MΦ and MΦ that have phagocytosed latex beads 24 h before were very similar. They evidenced a statistically significant difference for only 15 probe sets. None of the 29 corresponding probe sets in the mouse 430 DNA Affymetrix gene chip was present in the list of 1248 modulated probe sets observed in presence of *L. amazonensis *amastigotes. Thus, these data strongly support our conclusion that the gene expression profile observed 24 h after the phagocytosis of *L. amazonensis amastigotes *was due to the presence of intracellular cell-cycling parasites.

### *L. amazonensis *amastigotes set up an optimal sub cellular niche

#### Modulation of MΦ genes encoding vacuolar proton ATPase sub-units

Within their host cells, *L. amazonensis *amastigotes are known to multiply efficiently in the acidic environment of the MΦ PV [1]. In presence of amastigotes, we observed an up-regulation of the gene expression of eight isoforms of the V0 and V1 sub-units of the MΦ vacuolar proton ATPase (*atp6V0a1*, *atp6V0c*, *atp6V0d2*, *atp6V1a*, *atp6V1c1*, *atp6V1d*, *atp6V1g1 *and *atp6V1h*: +1.27 < FC < +2.32) [5]. This could contribute to the sustained acidification of the PV lumen which has been shown to be important at least for the optimal amastigote nutrient acquisition [6,7].

#### Coordinated modulation of MΦ lipid metabolism

**Figure 3:**
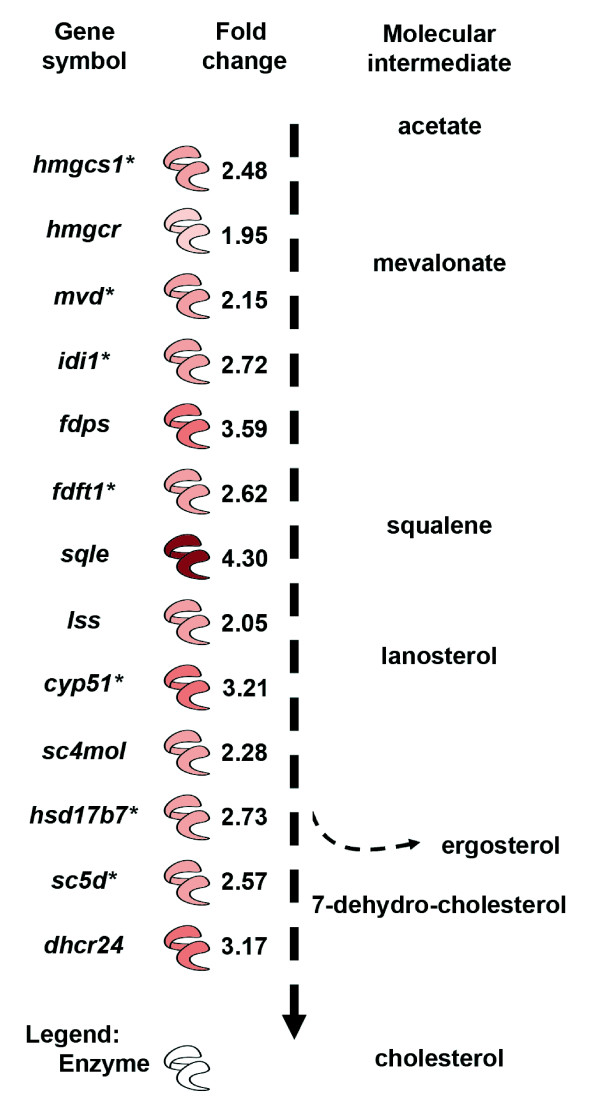
**Modulation of the sterol biosynthesis pathway in *L. amazonensis*-hosting MΦ**. *L. amazonensis*-hosting MΦ display an up-regulation of several genes involved in sterol biosynthesis (*, at least 2 probe-sets modulated).

The most relevant biological networks fitting our dataset were strongly associated to the function "lipid metabolism", the most significant canonical metabolic pathway being "biosynthesis of steroids" (*p*-value = 1.31e-02). Indeed, several up-regulated genes (Fig. 3, Table 1) were involved i) in cholesterol uptake (*ldlr*: + 4.68), ii) in cholesterol transport (*fabp4*: + 6.42 and *stard4*: + 2.31) and iii) in sterol biosynthesis (*hmgcs1*, *hmgcr*, *mvd*, *idi1*, *fdps*, *fdft1*, *sqle*, *lss*, *cyp51*, *sc4mol*, *hsd17b7*, *sc5d *and *dhcr24*: +1.95 < FC < +4.30). Worth is mentioning the most up-regulated gene encoding type II deiodinase (*dio2*, + 25.92), an enzyme converting intracellular thyroxin (T4) to tri-iodothyronine (T3), the more active form of thyroid hormone. It has previously been demonstrated in mouse hepatocytes that the molecular basis for the connection of T3 and cholesterol metabolism involves the master transcriptional activator of the aforementioned genes, namely *srebf2 *(+ 1.84) the promoter of which contains a thyroid hormone response element [8]. Furthermore, thyroid hormone receptors can activate transcription of target genes upon T3 binding and this could be facilitated by co-activators *ncoa4 *(+ 1.65) and *brd8 *(+ 1.08). Interestingly, opposite to *dio2*, the most down-regulated gene was cholesterol-25-hydrolase (*ch25h*: -6.57), an enzyme acting downstream this pathway by breaking down cholesterol and by synthesizing a co-repressor of *srebf2 *transcriptional activation [9]. Upstream this pathway, several up-regulated genes involved in glycolysis could also contribute to increase the supply of acetate (*acsl3, adhfe1, akr1a1, aldoa, aldoc, eno2, hk2, hk3, ldha, pfkl, pkg1 *and *pkm2*: +1.13 < FC < +2.61). Of note was the down-regulation of genes encoding enzymes competing i) with *hmgcs1 *for acetate (*acaca*: -1.32) and ii) with *aldoa *and *aldoc *for fructose, 1-6, biphosphate, which is needed to produce glyceraldehyde-3-phosphate upstream the sterol biosynthesis pathway (*fbp1*: -2.16). In addition, the up-regulation of the transcription factor encoded by *atf3 *(+ 1.77) was consistent with the down-modulation of *fbp1*. These data suggest that the available intracellular pool of sterol-synthesis molecular intermediates was maintained by a gene expression program relying on a coordinated regulation at both the transcriptional level by *srebf2*, *atf1 *(+ 1.84) and *atf3*, and also most likely at the post-transcriptional level by *insig1 *(+ 2.62) encoding a sterol-sensing protein that regulates the intracellular cholesterol level [10].

The expression of several genes involved in the fatty acid biosynthesis pathway was also up-regulated with the modulation of *ppap2b *(+ 8.53), *scd1 *(+ 2.68), *scd2 *(+ 2.45) and *acsl3 *(+ 2.09). Moreover, genes encoding fatty acid binding proteins that play a role in fatty acid uptake and transport were up-regulated (*fabp3*: + 2.29, *fabp4*: + 6.42 and *fabp5*: + 1.57). Extracellular lipolysis was down-modulated (*lipe*: -2.20, *lpl*: -1.44 and *apoc2*: -1.63), while intracellular catabolism of triglycerides mediated via *mgll *was up-regulated (+ 3.40). Fatty acid transport to peroxisome was diminished with *abcd2 *down-modulation (-2.11). Since this was not described neither for *L. major *nor *L. donovani *[11], this could be unique for the *L. mexicana *complex, all sub-species of which multiply within giant communal PV [1]. Indeed, previous experimental work performed with *L. mexicana *[12,13], which is very close to *L. amazonensis *(both share the same distinctive feature to multiply within a communal PV), has shown that amastigotes could take advantage of the MΦ sterol biosynthesis pathway to produce ergosterol.

These data were in agreement with the sterol biosynthesis machinery of the MΦ host cell being exploited by the cell-cycling amastigotes for both their own cell membrane sterols, in particular ergosterol and the PV membrane sterol-dependent remodelling. Indeed, cholesterol availability might play a role in the formation of the PV lipid rafts [14] that could be involved in the control of fusion events leading to the sustained remodelling of the huge communal PV membrane where the aforementioned proton pump components are regularly delivered.

#### Modulation of MΦ polyamine metabolism

**Figure 4:**
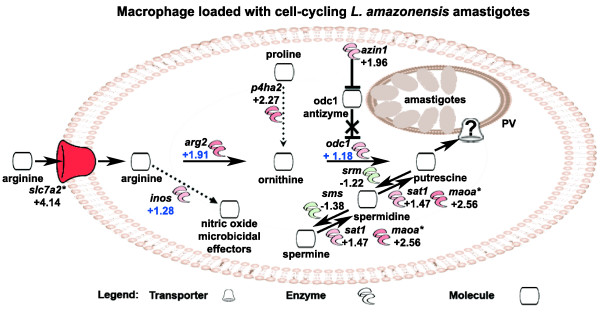
**Modulation of the polyamine biosynthesis pathways in *L. amazonensis*-hosting MΦ**. *L. amazonensis*-hosting MΦ display a gene expression coordination of several genes involved in polyamine biosynthesis (*, at least 2 probe-sets modulated; blue values determined by RTQPCR).

Polyamines (*e.g. *putrescine) derived from arginine catabolism are essential compounds for amastigote growth [15]. Using the Affymetrix technology we failed to detect, at the 5% significance threshold, arginase-2 (*arg2*) and ornithine decarboxylase-1 (*odc1*), two enzymes leading to the formation of polyamines through arginine catabolism. Indeed, while for *arg2 *the raw fluorescence intensity values were below or close to the background level, for *odc1 *the raw fluorescence intensities before data processing displayed only a slight increase (+ 1.21) in presence of amastigotes (see Additional file 1). However, the up-regulation of SLC7A2 (+ 4.14) in MΦ hosting amastigotes was a strong incentive for monitoring the abundance of *arg2 *and *odc1 *transcripts with a validated RTQPCR method. Using this method we did detect a slight variation of the expression of *arg2 *(+ 1.91) and *odc1 *(+ 1.18) (Table 1). Therefore, in presence of amastigotes, *arg2 *could favour arginine transformation into ornithine, the latter being catalyzed in turn by *odc1 *to generate putrescine (Fig. 4).

ODC1-antizyme plays a role in the regulation of polyamine synthesis by binding to and inhibiting ODC1. The transcript abundance of *azin1 *encoding ODC1-antizyme inhibitor-1 was higher (+ 1.96) when amastigotes were present, so that this inhibitor might prevent antizyme-mediated ODC1 degradation. Of note, ornithine could also be generated from proline by *p4ha2 *(+ 2.27), and putrescine from spermine and spermidine by the successive action of *sat1 *(+ 1.47) and *maoa *(+ 2.56). Spermidine synthase (*srm*) and spermine synthase (*sms*), two enzymes catalyzing the reverse reactions leading to the formation of spermine from putrescine, were not detected with Affymetrix (5% threshold), although their transcript abundance decreased in presence of amastigotes (-1.22 and -1.38, respectively; see Additional file 1). No gene expression modulation was detected with Affymetrix for *nos2 *(5% threshold) that encodes a competing enzyme for arginine substrate leading to the production of microbe-targeting nitric oxide derivatives (fluorescence intensity was below the background level, see Additional file 1), and only a slight up-regulation was detected with RTQPCR (+ 1.28) (Table 1). The present data further extend former observations [15,16], and highlight a coordinated gene expression modulation that sustains a metabolic flux leading to the biosynthesis of putrescine from arginine and proline *via *ornithine, and from spermine and spermidine.

### *L. amazonensis *amastigotes set up an optimal dermis niche

#### Decreased expression of genes involved in the entry of non leishmanial micro-organisms as well as in the sensing and processing of microbial molecules

Several genes involved in classical and alternate complement component pathways were down-regulated (*c1qa*, *c1qb*, *serping1*, *c3*, *c4b*, *cfh*, *c5ar1 *and *pros1*: -2.80 < FC < -1.35) as well as some genes of the Toll-like receptor signalling pathway (*tlr2*, *tlr7*, *tlr8*, *cd14*, *mapk14*, *c-fos *and *nfkbia*: -3.11 < FC < -1.61. Furthermore, the negative regulator *tollip *also was up-regulated (+ 1.69). These pathways are known to contribute to the entry of micro-organisms and the sensing/processing of microbial molecules. In presence of the intracellular cell-cycling amastigotes these biological processes would be restricted, if not prevented. Indeed, it is conceivable that non-*Leishmania *micro-organisms or microbial molecules might trigger a different MΦ transcriptional program that could interfere with the one already set up by *L. amazonensis *amastigotes for their multiplication. Nevertheless, it has recently been demonstrated that the other *L. amazonensis *developmental stage, the promastigote, was still able to enter MΦ already hosting amastigotes, to transform into amastigote and to multiply efficiently within the PV [17].

The above data suggested that *L. amazonensis *amastigotes were able to control MΦ expression of the early complement components, the proteolytic products of which are known to be pro-inflammatory. This complement component pathway down-modulation was also recently described for human MΦ housing *L. major *parasites [18]. The down-modulation of the Toll-like receptor pathway also suggested prevention of the inflammatory process signalling. At this stage, although some anti-inflammatory genes were not up-modulated (*il10*: -2.97 and *il10ra: *-2.16) the gene expression modulation for the majority of the listed genes involved in inflammatory processes showed that the presence of cell-cycling amastigotes imposed an immune unbalance favouring the shaping of a counter-inflammatory and safe dermis niche for these parasites (*il1rn*, *il1b*, *il11ra1*, *il17rb, il18, socs6, cd200, nfkbia, relB, c-fos *and *anxA1*, an inhibitor of phospholipase A2 mediated-inflammation: 1.41 < | FC | < 4.19).

#### Decreased expression of genes involved in the chemokine-dependent MΦ traffic

The down-modulation of the expression of genes encoding chemokine receptors (*ccr2*, *ccr3*, *cx3cr1 *and *cmklr1*: -2.65 < FC < -1.83) suggested that amastigote-harbouring MΦ were less responsive to chemo-attractant gradients and thus less amenable to enter into the afferent lymphatics. This is consistent with the dominant residence of *L. amazonensis*-hosting MΦ in the skin. In favour of this possible reduced emigration of MΦ from the dermis niche was the down-regulation of *itga4 *(-2.06) encoding an integrin shown to contribute to the lymphatic adhesion/transmigration [19]. It is beyond the scope of this article to discuss about more than a dozen of chemokine receptor ligands the gene expression of which was modulated (see Additional file 1). Indeed, the interpretation is not that straightforward because of the complexity of their partial overlapping functions and/or common receptors.

#### Decreased expression of genes involved in the cellular communication with leukocytes prone to display parasite-damaging functions

The modulation of several transcripts indicated a prevention of MΦ communication with leukocytes that could be rapidly recruited such as NK lymphocytes, and T-lymphocytes. For instance, H60 is one of the ligand able to efficiently activate NK-lymphocytes by binding to the NKG2D receptor (encoded by *klrk1*). In presence of amastigotes, the *h60 *MΦ expression was down-modulated (-2.07), suggesting the prevention of this "immune synapse" by which parasitized MΦ and NK lymphocytes can communicate. Interestingly, NKG2D receptor engagement by H60 ligand in MΦ, that normally leads to the production of MΦ leishmanicidal molecules such as NO and TNF-α [20], could be impaired in MΦ hosting amastigotes since the expression of *klrk1 *gene was also down-modulated (-1.72). Besides, the gene expression of the co-stimulatory molecule CD86 was reduced (-1.83), while that of the inhibitory receptor CD274 (also referred to as B7-H1) was increased (+ 1.93). In addition, the transcript abundance of the co-stimulatory molecules ICAM1 (-1.75), ICAM2 (-1.85) and LFA-1 (or integrin-alpha L, – 2.0) was also reduced. The down-modulation of several genes involved in antigen presentation by MHC class II molecules was recently discussed for human MΦ housing *L. major *parasites [18]. This data suggested plausible reduced effectiveness of this other "immune synapse" involving TCR-dependent signalling by which MΦ and T-lymphocytes can communicate. Consistent with this was the reduced transcription level in MΦ hosting *L. amazonensis *amastigotes of *h-2ma *(-1.88) and of *ifngr1 *(-1.83 FC) that encodes the receptor for IFNγ, a cytokine secreted by both activated NK- and T-lymphocytes and involved upstream the MHC class II gene up-regulation.

## Conclusion

The Affymetrix GeneChip technology has allowed – for many cell lineages – the global analysis of several thousand transcripts simultaneously to be carried out in a robust fashion [21]. The remarkable coordination of gene expression as well as coherent biological interaction networks displayed by MΦ subverted as host cells by the multiplying *L. amazonensis *amastigotes allow highlighting the power of this technology at two different levels: (a) the amastigote-hosting MΦ transcriptional features *per se *and (b) the features of MΦ hosting cell-cycling amastigotes which would have been captured within the dermal environment. Further *in vivo *quantitative analysis will have to be set up for validating or not the present transcriptional profile at early stage after the first wave of amastigote multiplication in the ear dermis of naïve BALB/c mice. Overall, the gene expression profile of MΦ hosting amastigotes did not strictly fall into either of the MΦ "activation" profiles, as it was also the case for *L. chagasi *[22]. Nevertheless, consistent with the multiplication of the amastigote developmental stage, some overlap with features of the alternative MΦ activation could be observed, such as the up-regulation of *arg2 *and *il1rn*, and the down-regulation of *cd14 *(-1.73 FC).

In addition to the conversion of the MΦ arginine metabolism from a parasite-damaging pathway to a parasite-supportive one, the most clear-cut and novel output of the present analysis was the up-regulation of the MΦ fatty acid biosynthesis pathway. Coupled to the polyamine biosynthesis the MΦ lipids could not only be a source of nutrients for the amastigotes but could also contribute to the PV unique membrane features [2,23]. Lipids could not only influence the PV membrane curvature but also coordinate the recruitment and retention of key protein export to the PV where multiplying amastigotes are known to be attached [2]. This makes it conceivable that the multiplying amastigotes could take up trophic resources and sense non-trophic signals.

We have highlighted a promising set of transcripts accounting for the BALB/c mouse macrophage reprogrammed as cell-cycling amastigote hosting cells. We do not ignore that transcript modulation changes revealed by microarray analysis could be uncoupled to changes revealed by proteomic and phosphoproteomic analysis. We did not explore how these mRNA changes manifest at the level of the proteome but the present genomewide data will provide a unique resource (a) against which to compare any proteomic/phosphoproteomic data (b) to allow identifying novel small compounds displaying static or cidal activity towards cell-cycling amastigotes hosted within the macrophage PV. Indeed the readout assay we designed allows high content imaging in real time of (a) the amastigotes (b) the amastigotes-hosting PV as well as the macrophages *per se *[24] and can be up-scaled for high throughput screening of small compound libraries.

## Methods

### Mice, MΦ and amastigotes

Swiss *nu/nu *and BALB/c mice were used (following National Scientific Ethics Committee guidelines) for *L. amazonensis *(LV79, MPRO/BR/1972/M1841) amastigote propagation and bone marrow-derived MΦ preparation, respectively. Four amastigotes per MΦ were added. Parasite-harbouring MΦ (>98%) and parasite-free ones were cultured at 34°C either for 24 h for transcriptomic studies or for different time periods for microscopy analyses [25].

### Kinetic study of the intracellular amastigote population size

At different time points post amastigote addition, MΦ cultures were processed for immunofluorescence and phase contrast microscopy. Briefly, MΦ cultures on coverslips were fixed, permeabilized, incubated with the amastigote-specific mAb 2A3.26 and Texas Red-labelled conjugate, stained with Hoechst 33342 and mounted in Mowiol for observation under an inverted microscope as previously described [25]. Ratios of amastigotes per MΦ (between 200 and 700 MΦ nuclei being counted) were calculated and expressed as mean numbers of amastigotes per MΦ at each time point.

### GeneChip hybridization and data analysis

Total RNA were extracted from MΦ (RNeasy+ Mini-Kit, Qiagen), their quality control (QC) and concentration were determined using NanoDrop ND-1000 micro-spectrophotometer and their integrity was assessed [26] using Agilent-2100 Bioanalyzer (RNA Integrity Numbers ≥ 9). Hybridizations were performed following the Affymetrix protocol . MIAME-compliant data are available through ArrayExpress and GEO databases , accession: E-MEXP-1595; , accession: GSE11497). Based on AffyGCQC program QC assessment [27], hybridizations of biological duplicates were retained for downstream analysis. Raw data were pre-processed to obtain expression values using GC-RMA algorithm [28]. Unreliable probe-sets called "absent" by Affymetrix GCOS software  for at least 3 GeneChips out of 4 were discarded, as well as probe-sets called "absent" once within samples plus once within controls. LPE tests [29] were performed to identify significant differences in gene expression between parasite-free and parasite-harbouring MΦ. Benjamini-Hochberg (BH) multiple-test correction [30] was applied to control for the number of false positives with an adjusted 5% statistical significance threshold. A total of 1,248 probe-sets showing significant differential expression were input into Ingenuity Pathway Analysis software v5.5.1  to perform a biological interaction network analysis. Although a cross-hybridization study was performed by Gregrogy and coworkers (11) on a mouse U74av2 DNA Affymetrix gene chip (12,488 transcripts) with RNA from *Leishmania donovani*, it was important to also assess the absence of significant cross-hybridization in our experimental conditions. To this end, we compared the gene chip data obtained with MΦ RNA alone with those obtained with the same RNA preparation spiked with different amount of *L. amazonensis *RNA. Our data showed that *L. amazonensis *RNA did not interfere with mouse RNA hybridization onto GeneChips (data not shown). Indeed, fold-change values for a technical replicate of mouse RNA were not significantly different from those observed for mouse RNA spiked with up to 10% of *L. amazonensis *RNA taking the non-spiked mouse RNA as reference (one-sample one-sided Student's t-test P-values < 5% for all 45,101 probe-sets, the 1,248 significantly modulated probe-sets, the probe-sets of the 107 genes in Table 1 and the probe-sets of the 13 genes in Figure 3). Therefore, the observed over-expressions were not due to cross-hybridization between the mouse and the amastigote transcripts, thus providing valid information about the reprogramming of MΦ hosting cell-cycling amastigotes.

### Real-time quantitative PCR

RTQPCR were performed on cDNA from various biological samples including those used for the hybridizations using a LightCycler^®^480 (Roche Diagnostics). Primer sequences are available upon request. Gene expression analysis using qBase [31] allowed determining the normalized relative quantities between parasite-free and parasite-harbouring MΦ.

## Authors' contributions

JOF performed the hybridization experiments, the bioinformatical, statistical and pathway analyses, prepared most of the figures and tables and drafted the manuscript. ELL contributed to the pathway analysis and participated in RTQPCR assays and analyses. BR was involved in the design of the study and participated in hybridization experiments and statistical analyses. JYC reviewed the manuscript. GM participated in the conception of the study, in its design and coordination and contributed to draft the manuscript. TL participated in the conception of the study, in its design and coordination and reviewed the manuscript. EP was involved in the conception, the design and the coordination of the study, prepared and carried out the *in vitro *experiments and the RNA isolations, performed the RT-qPCR assays and analyses, participated in the preparation of figures and tables, in the analysis of the data and in manuscript preparation. All authors approved the manuscript and they have no conflicting financial interests.

## Supplementary Material

Additional file 1 **This table lists all the probe-sets that were significantly modulated in MΦ housing multiplying amastigotes compared to uninfected ones.** Annotation files are updated quarterly on Affymetrix Support web site . Click here for file
