# Supplementary material for: Transcriptional signatures of BALB/c mouse macrophages housing multiplying Leishmania amazonensis amastigotes
Source: BMC Genomics. 2009 Mar 20;10:119. doi: 10.1186/1471-2164-10-119 (PMC2666765; doi:10.1186/1471-2164-10-119)
Supplement: Additional file 1 — This table lists all the probe-sets that were significantly modulated in MΦ housing multiplying amastigotes compared to uninfected ones. Annotation files are updated quarterly on Affymetrix Support web site . [file 1471-2164-10-119-S1.pdf]

Table 1
List of the 1,248 probe-sets showing significant differential expression at the 5% adjusted significance level

**Definition of column names:**	
**Probe Name**	Affymetrix probe set ID (https://www.affymetrix.com/analysis/netaffx/index.affx)
**Acc. Num.**	UniGene ID (http://www.ncbi.nlm.nih.gov/sites/entrez?db=unigene) obtained automatically from the last update (March 19th 2008; Mouse430_2.na25.annot.csv.zip annotation file available at http://www.affymetrix.com/support/technical/annotationfilesmain.affx)
**LocusLink**	Entrez gene ID (http://www.ncbi.nlm.nih.gov/sites/entrez?db=gene) obtained automatically from the last update (March 19th 2008; Mouse430_2.na25.annot.csv.zip annotation file available at http://www.affymetrix.com/support/technical/annotationfilesmain.affx / NA stands for "Not Available"
**Gene Name**	Gene name (when available) obtained automatically from the last update (March 19th 2008; Mouse430_2.na25.annot.csv.zip annotation file available at http://www.affymetrix.com/support/technical/annotationfilesmain.affx / NA stands for "Not Available"
**Gene Symbol**	Gene symbol (when available) obtained automatically from the last update (March 19th 2008; Mouse430_2.na25.annot.csv.zip annotation file available at http://www.affymetrix.com/support/technical/annotationfilesmain.affx / NA stands for "Not Available"
**Stat.**	LPE test statistic
**P**	LPE test statistic *p*-value
**Adj. P**	Adusted *p*-value (*p*-value corrected for multiple testing using Benjamini & Hochberg method)
**mean I**	Mean signal within Infected samples (log(base 2))
**mean NI**	Mean signal within Non Infected samples (log(base 2))
**Fold Change**	log(base 2) fold change = mean.I - mean.NI (green color is used for down-regulated genes while red is used for up-regulated ones)
**FC**	Linear fold change (green color is used for down-regulated genes while red is used for up-regulated ones)

	A	B	C	D	E	F	G	H	I	J	K	L	M
1	Probe Name	Acc. Num	LocusLink	Gene Name	Gene Symbol	Stat.	P	Adj. P	mean I	mean NI	Fold Change	FC	Ensembl
2	1415723_at	BQ176989	217869	eukaryotic translation initiation factor 5	Eif5	3.56	3.65E-04	1.33E-02	11.20	10.40	0.81	1.75	
3	1415749_a_at	NM_017475	54170	Ras-related GTP binding C	Rragc	3.30	9.59E-04	2.86E-02	13.20	12.50	0.71	1.64	
4	1415810_at	BB702754	18140	ubiquitin-like, containing PHD and RING finger domains, 1	Uhrf1	-3.54	4.04E-04	1.44E-02	5.24	6.25	-1.01	-2.02	
5	1415822_at	BG060909	20250	stearoyl-Coenzyme A desaturase 2	Scd2	4.32	1.54E-05	9.89E-04	12.00	11.00	0.99	1.99	
6	1415823_at	BG060909	20250	stearoyl-Coenzyme A desaturase 2	Scd2	4.43	9.40E-06	6.56E-04	8.55	7.49	1.05	2.08	
7	1415824_at	BG060909	20250	stearoyl-Coenzyme A desaturase 2	Scd2	5.77	7.86E-09	1.32E-06	10.70	9.42	1.29	2.45	
8	1415826_at	NM_133826	108664	ATPase, H+ transporting, lysosomal V1 subunit H	Atp6v1h	3.37	7.62E-04	2.39E-02	10.60	9.85	0.75	1.69	
9	1415834_at	NM_026268	67603	dual specificity phosphatase 6	Dusp6	-5.64	1.70E-08	2.65E-06	9.18	10.50	-1.27	-2.42	
10	1415871_at	NM_009369	21810	transforming growth factor, beta induced	Tgfbi	-8.35	6.99E-17	4.92E-14	7.74	9.72	-1.98	-3.94	
11	1415886_at	AB043953	27387	SH2 domain containing 3C	Sh2d3c	5.62	1.88E-08	2.92E-06	7.40	5.86	1.54	2.90	
12	1415911_at	NM_008378	16210	imprinted and ancient	Impact	3.58	3.46E-04	1.28E-02	9.39	8.59	0.80	1.74	
13	1415922_s_at	NM_010807	17357	MARCKS-like 1	Marcksl1	-4.74	2.17E-06	1.87E-04	9.17	10.20	-1.06	-2.09	
14	1415936_at	NM_013867	29815	breast cancer anti-estrogen resistance 3	Bcar3	3.31	9.44E-04	2.83E-02	9.36	8.62	0.74	1.67	
15	1415943_at	BI788645	20969	syndecan 1	Sdc1	9.95	0.00E+00	0.00E+00	10.00	7.77	2.27	4.84	
16	1415944_at	BI788645	20969	syndecan 1	Sdc1	7.99	1.33E-15	7.89E-13	8.50	6.46	2.04	4.11	
17	1415945_at	NM_008566	17218	minichromosome maintenance deficient 5, cell division cycle 46 (S. cerevisiae)	Mcm5	-3.75	1.77E-04	7.47E-03	5.67	6.72	-1.05	-2.08	
18	1415960_at	NM_010824	17523	myeloperoxidase	Mpo	-3.97	7.23E-05	3.64E-03	5.21	6.34	-1.13	-2.19	
19	1415964_at	NM_009127	20249	stearoyl-Coenzyme A desaturase 1	Scd1	5.06	4.30E-07	4.50E-05	6.17	4.75	1.42	2.68	
20	1415965_at	NM_009127	20249	stearoyl-Coenzyme A desaturase 1	Scd1	6.17	6.72E-10	1.45E-07	4.77	3.38	1.40	2.64	
21	1415993_at	NM_009270	20775	squalene epoxidase	Sqle	8.71	0.00E+00	0.00E+00	9.17	7.06	2.10	4.30	
22	1416010_a_at	NM_010119	13660	EH-domain containing 1	Ehd1	-5.51	3.64E-08	5.18E-06	8.83	10.10	-1.25	-2.37	
23	1416011_x_at	NM_010119	13660	EH-domain containing 1	Ehd1	-6.39	1.70E-10	4.12E-08	7.87	9.37	-1.50	-2.83	
24	1416013_at	NM_011116	18807	phospholipase D family, member 3	Pld3	3.28	1.04E-03	3.05E-02	13.40	12.70	0.68	1.61	
25	1416021_a_at	BC002008	16592 /// 547041 /// 620603	fatty acid binding protein 5, epidermal /// similar to Fatty acid-binding protein, epidermal (E-FABP) (Psoriasis-associated fatty acid-binding protein homolog) (PA-FABP) (Keratinocyte lipi binding protein) /// similar to Fatty acid binding protein 5, epidermal	Fabp5 /// LOC547041 /// LOC620603	4.13	3.62E-05	2.03E-03	15.00	14.60	0.43	1.34	
26	1416022_at	BC002008	16592	fatty acid binding protein 5, epidermal	Fabp5	6.36	1.99E-10	4.70E-08	15.20	14.50	0.65	1.57	
27	1416023_at	NM_010174	14077	fatty acid binding protein 3, muscle and heart	Fabp3	5.01	5.50E-07	5.58E-05	8.63	7.43	1.19	2.29	
28	1416030_a_at	NM_008568	17220	minichromosome maintenance deficient 7 (S. cerevisiae)	Mcm7	-4.94	7.67E-07	7.48E-05	5.75	7.11	-1.36	-2.57	
29	1416031_s_at	NM_008568	17220	minichromosome maintenance deficient 7 (S. cerevisiae)	Mcm7	-3.18	1.49E-03	4.00E-02	6.05	6.93	-0.88	-1.84	
30	1416034_at	NM_009846	12484	CD24a antigen	Cd24a	4.74	2.14E-06	1.85E-04	7.58	6.32	1.26	2.40	
31	1416041_at	NM_011361	20393	serum/glucocorticoid regulated kinase 1	Sgk1	4.22	2.40E-05	1.45E-03	12.40	11.40	0.96	1.95	
32	1416067_at	NM_013562	15982	interferon-related developmental regulator 1	Ifrd1	5.68	1.34E-08	2.13E-06	11.40	10.10	1.29	2.44	
33	1416076_at	NM_007629	12429 /// 268697 /// 434175 /// 667005	cyclin B1, related sequence 1 /// cyclin B1 /// predicted gene, EG434175 /// similar to cyclin B1	Ccnb1 /// Ccnb1-rs1 /// EG434175 /// LOC667005	-3.58	3.41E-04	1.27E-02	3.27	4.00	-0.73	-1.66	
34	1416084_at	AA124553	100047896 /// 22682	zinc finger, AN1-type domain 5 /// similar to zinc finger protein ZNF216	LOC100047896 /// Zfand5	3.54	4.02E-04	1.44E-02	11.20	10.40	0.81	1.75	
35	1416085_s_at	AA124553	100047896 /// 22682	zinc finger, AN1-type domain 5 /// similar to zinc finger protein ZNF216	LOC100047896 /// Zfand5	3.14	1.71E-03	4.46E-02	12.50	11.70	0.72	1.64	
36	1416097_at	NM_138682	192198	leucine rich repeat containing 4	Lrrc4	-6.19	6.19E-10	1.34E-07	4.91	6.62	-1.72	-3.29	
37	1416122_at	NM_009829	12444	cyclin D2	Ccnd2	-5.96	2.47E-09	4.72E-07	7.18	8.64	-1.46	-2.76	
38	1416156_at	NM_009502	22330	vinculin	Vcl	4.91	9.12E-07	8.68E-05	9.11	7.99	1.12	2.17	
39	1416157_at	NM_009502	22330	vinculin	Vcl	3.73	1.88E-04	7.90E-03	5.71	4.66	1.05	2.07	
40	1416250_at	NM_007570	12227	B-cell translocation gene 2, anti-proliferative	Btg2	-3.61	3.02E-04	1.15E-02	7.19	8.08	-0.90	-1.86	
41	1416251_at	NM_008567	17219	minichromosome maintenance deficient 6 (MIS5 homolog, S. pombe) (S. cerevisiae)	Mcm6	-4.30	1.68E-05	1.06E-03	8.43	9.41	-0.98	-1.98	
42	1416258_at	NM_009387	21877	thymidine kinase 1	Tk1	-5.96	2.50E-09	4.76E-07	4.21	5.83	-1.62	-3.07	
43	1416268_at	BC005486	23872	E26 avian leukemia oncogene 2, 3' domain	Ets2	-5.09	3.58E-07	3.79E-05	6.96	8.23	-1.27	-2.42	
44	1416273_at	NM_009396	21928	tumor necrosis factor, alpha-induced protein 2	Tnfaip2	6.59	4.42E-11	1.19E-08	10.10	8.64	1.47	2.76	
45	1416309_at	BC009096	108907	nucleolar and spindle associated protein 1	Nusap1	-3.64	2.71E-04	1.06E-02	4.47	5.49	-1.02	-2.02	
46	1416345_at	W82151	30058	translocase of inner mitochondrial membrane 8 homolog a1 (yeast)	Timm8a1	3.43	6.03E-04	1.99E-02	9.19	8.41	0.77	1.71	
47	1416382_at	NM_009982	13032	cathepsin C	Ctsc	-5.24	1.60E-07	1.89E-05	13.60	14.40	-0.76	-1.70	
48	1416412_at	NM_010945	18201	neutral sphingomyelinase (N-SMase) activation associated factor	Nsmaf	-3.42	6.26E-04	2.05E-02	7.04	7.90	-0.86	-1.82	
49	1416416_x_at	NM_010358	14862	glutathione S-transferase, mu 1	Gstm1	6.58	4.84E-11	1.29E-08	11.00	9.49	1.48	2.79	
50	1416424_at	BC011116	66905	mannose-6-phosphate receptor binding protein 1	M6prbp1	3.26	1.13E-03	3.22E-02	9.45	8.72	0.73	1.65	
51	1416442_at	NM_010499	15936	immediate early response 2	Ier2	-3.18	1.50E-03	4.01E-02	7.64	8.40	-0.76	-1.69	
52	1416469_at	BB705823	269593	leucine zipper protein 1	Luzp1	-3.20	1.39E-03	3.80E-02	8.13	8.87	-0.74	-1.67	
53	1416505_at	NM_010444	15370	nuclear receptor subfamily 4, group A, member 1	Nr4a1	-8.84	9.31E-19	6.87E-16	5.03	7.40	-2.37	-5.16	
54	1416519_at	NM_018730	100043483 /// 100048508 /// 54217 /// 622534	ribosomal protein L36 /// similar to Rpl36 protein	LOC100043483 /// LOC100048508 /// LOC622534 /// Rpl36	-3.50	4.72E-04	1.65E-02	13.60	14.20	-0.53	-1.45	
55	1416552_at	NM_025274	434423	developmental pluripotency associated 5A	Dppa5a	3.70	2.18E-04	8.94E-03	3.29	2.77	0.52	1.44	
56	1416592_at	AF276917	93692	glutaredoxin	Glrx	-3.27	1.08E-03	3.14E-02	10.90	11.60	-0.77	-1.70	
57	1416593_at	AF276917	93692	glutaredoxin	Glrx	-3.60	3.16E-04	1.19E-02	10.70	11.50	-0.84	-1.79	
58	1416614_at	BC010712	58521	EP300 interacting inhibitor of differentiation 1	Eid1	-3.48	5.02E-04	1.72E-02	11.90	12.70	-0.80	-1.74	
59	1416625_at	NM_009776	12258	serine (or cysteine) peptidase inhibitor, clade G, member 1	Serping1	-5.04	4.68E-07	4.84E-05	2.26	2.70	-0.43	-1.35	
60	1416672_s_at	NM_053177	94178	mucolipin 1	Mcoln1	3.11	1.89E-03	4.79E-02	9.28	8.59	0.70	1.62	
61	1416698_a_at	NM_016904	54124	CDC28 protein kinase 1b	Cks1b	-3.47	5.20E-04	1.77E-02	9.25	10.00	-0.78	-1.71	
62	1416700_at	BC009002	74194	Rho family GTPase 3	Rnd3	-3.26	1.11E-03	3.18E-02	4.88	5.81	-0.93	-1.91	
63	1416703_at	BC012235	26416	mitogen activated protein kinase 14	Mapk14	-3.09	1.99E-03	4.97E-02	9.55	10.20	-0.69	-1.61	
64	1416723_at	AI639846	21413	transcription factor 4	Tcf4	-3.60	3.16E-04	1.19E-02	10.10	11.00	-0.82	-1.77	
65	1416724_x_at	AI639846	21413	transcription factor 4	Tcf4	-3.39	7.01E-04	2.23E-02	9.14	9.90	-0.76	-1.69	
66	1416759_at	NM_138315	171580	microtubule associated monoxygenase, calponin and LIM domain containing 1	Mical1	3.34	8.42E-04	2.59E-02	7.73	6.87	0.86	1.81	
67	1416774_at	NM_009516	22390	wee 1 homolog (S. pombe)	Wee1	3.27	1.07E-03	3.11E-02	6.77	5.85	0.92	1.89	
68	1416802_a_at	NM_026410	67849	cell division cycle associated 5	Cdca5	-3.11	1.88E-03	4.78E-02	6.19	7.04	-0.85	-1.80	
69	1416805_at	NM_133187	68659	RIKEN cDNA 1110032E23 gene	1110032E23Rik	8.88	0.00E+00	0.00E+00	10.70	8.66	1.99	3.98	RIKEN cDNA 1110032E23 gene (Uncharacterized protein C4orf18 homolog)
70	1416811_s_at	NM_007796	13024 /// 13025	cytotoxic T lymphocyte-associated protein 2 alpha /// cytotoxic T lymphocyte-associated protein 2 beta	Ctla2a /// Ctla2b	-6.36	1.96E-10	4.66E-08	9.94	11.40	-1.47	-2.77	
71	1416827_at	NM_011539	21391	thromboxane A synthase 1, platelet	Tbxas1	-3.24	1.19E-03	3.36E-02	9.25	9.98	-0.72	-1.65	
72	1416840_at	NM_026524	68041	Mid1 interacting protein 1 (gastrulation specific G12-like (zebrafish))	Mid1ip1	3.22	1.26E-03	3.51E-02	11.30	10.50	0.74	1.67	
73	1416878_at	AI594880	66493	mitochondrial ribosomal protein L51	Mrpl51	-4.02	5.73E-05	3.02E-03	3.09	3.85	-0.75	-1.69	
74	1416921_x_at	NM_007438	11674	aldolase 1, A isoform	Aldoa	3.11	1.89E-03	4.79E-02	13.00	12.30	0.68	1.61	
75	1416933_at	NM_008898	18984	P450 (cytochrome) oxidoreductase	Por	3.24	1.20E-03	3.37E-02	12.00	11.30	0.75	1.68	
76	1416951_a_at	NM_023721	73834	ATPase, H+ transporting, lysosomal V1 subunit D	Atp6v1d	3.19	1.41E-03	3.85E-02	10.50	9.78	0.71	1.64	
77	1416952_at	NM_023721	73834	ATPase, H+ transporting, lysosomal V1 subunit D	Atp6v1d	3.77	1.62E-04	6.97E-03	11.90	11.10	0.87	1.82	
78	1416978_at	NM_010189	14132	Fc receptor, IgG, alpha chain transporter	Fcgrt	-4.07	4.62E-05	2.50E-03	9.57	10.50	-0.91	-1.89	
79	1417010_at	NM_013915	30928	zinc finger protein 238	Zfp238	4.51	6.60E-06	4.84E-04	10.40	9.43	1.00	2.01	
80	1417019_a_at	NM_011799	23834	cell division cycle 6 homolog (S. cerevisiae)	Cdc6	-4.73	2.29E-06	1.96E-04	4.75	6.08	-1.33	-2.52	
81	1417023_a_at	NM_024406	11770	fatty acid binding protein 4, adipocyte	Fabp4	12.97	0.00E+00	0.00E+00	13.50	10.80	2.68	6.42	
82	1417031_at	NM_025864	66950	RIKEN cDNA 2310028N02 gene	2310028N02Rik	4.12	3.76E-05	2.10E-03	8.64	7.67	0.97	1.96	RIKEN cDNA 2310028N02 gene (Transmembrane protein C1orf75 homolog)
83	1417063_at	NM_009777	12260	complement component 1, q subcomponent, beta polypeptide	C1qb	-4.60	4.21E-06	3.31E-04	13.00	13.80	-0.82	-1.77	
84	1417111_at	BB070019	17155	mannosidase 1, alpha	Man1a	-3.83	1.30E-04	5.93E-03	9.21	10.10	-0.86	-1.81	
85	1417116_at	BG069516	102857	solute carrier family 6 (neurotransmitter transporter, creatine), member 8	Slc6a8	6.69	2.27E-11	6.51E-09	11.40	9.89	1.51	2.85	
86	1417145_at	NM_133921	100978	nuclear transcription factor, X-box binding-like 1	Nfxl1	-6.59	4.51E-11	1.21E-08	8.51	10.00	-1.51	-2.84	
87	1417149_at	NM_011031	18452	procollagen-proline, 2-oxoglutarate 4-dioxygenase (proline 4-hydroxylase), alpha II polypeptide	P4ha2	4.14	3.49E-05	1.96E-03	6.33	5.15	1.18	2.27	
88	1417216_at	NM_138606	18715	proviral integration site 2	Pim2	3.56	3.70E-04	1.35E-02	3.68	3.06	0.62	1.54	
89	1417268_at	NM_009841	12475	CD14 antigen	Cd14	-4.21	2.59E-05	1.54E-03	12.90	13.60	-0.79	-1.73	
90	1417292_at	NM_008330	15953	interferon gamma inducible protein 47	Ifi47	-3.10	1.96E-03	4.93E-02	10.90	11.60	-0.73	-1.66	

	A	B	C	D	E	F	G	H	I	J	K	L	M
91	1417296_at	NM_007497	100047421 /// 11908	activating transcription factor 1 /// similar to activating transcription factor 1	Atf1 /// LOC100047421	3.93	8.52E-05	4.20E-03	10.70	9.82	0.88	1.84	
92	1417300_at	NM_133888	100340	sphingomyelin phosphodiesterase, acid-like 3B	Smpdl3b	-5.85	4.95E-09	8.61E-07	5.98	7.55	-1.57	-2.96	
93	1417303_at	NM_138656	192156	mevalonate (diphospho) decarboxylase	Mvd	4.44	8.96E-06	6.33E-04	4.92	3.82	1.10	2.15	
94	1417308_at	NM_011099	18746	pyruvate kinase, muscle	Pkm2	3.13	1.77E-03	4.57E-02	13.70	13.10	0.60	1.51	
95	1417323_at	NM_019976	56742	proline/serine-rich coiled-coil 1	Psrc1	-3.36	7.70E-04	2.41E-02	5.80	6.74	-0.94	-1.92	
96	1417381_at	NM_007572	12259	complement component 1, q subcomponent, alpha polypeptide	C1qa	-3.27	1.09E-03	3.15E-02	13.20	13.80	-0.57	-1.48	
97	1417400_at	NM_030690	75646	retinoic acid induced 14	Rai14	6.29	3.25E-10	7.43E-08	8.49	6.93	1.56	2.95	
98	1417401_at	NM_030690	75646	retinoic acid induced 14	Rai14	4.72	2.41E-06	2.05E-04	6.65	5.31	1.34	2.52	
99	1417435_at	BG261907	16795	like-glycosyltransferase	Large	3.47	5.18E-04	1.77E-02	8.08	7.22	0.86	1.81	
100	1417450_a_at	NM_011524	21335	transforming, acidic coiled-coil containing protein 3	Tacc3	-4.18	2.87E-05	1.66E-03	4.43	5.60	-1.16	-2.24	
101	1417483_at	AB026551	80859	nuclear factor of kappa light polypeptide gene enhancer in B-cells inhibitor, zeta	Nfkbiz	-7.37	1.65E-13	7.02E-11	6.11	8.04	-1.93	-3.81	
102	1417495_x_at	BB332449	12870	ceruloplasmin	Cp	-7.02	2.16E-12	7.85E-10	3.49	5.23	-1.74	-3.34	
103	1417505_s_at	BC004619	100038993 /// 16157 /// 16158	interleukin 11 receptor, alpha chain 1 /// interleukin 11 receptor, alpha chain 2 /// similar to interleukin 11 receptor, alpha chain 2	Il11ra1 /// Il11ra2 /// LOC100038993	4.88	1.06E-06	9.89E-05	8.59	7.42	1.17	2.24	
104	1417523_at	AF181829	56193	pleckstrin	Plek	4.24	2.25E-05	1.39E-03	12.20	11.20	0.97	1.96	
105	1417534_at	NM_010580	16419	integrin beta 5	Itgb5	-3.18	1.50E-03	4.01E-02	10.00	10.70	-0.72	-1.64	
106	1417542_at	BB737182	20112	ribosomal protein S6 kinase, polypeptide 2	Rps6ka2	7.02	2.19E-12	7.90E-10	8.43	6.66	1.78	3.42	
107	1417543_at	BB737182	20112	ribosomal protein S6 kinase, polypeptide 2	Rps6ka2	7.46	8.42E-14	3.72E-11	5.96	4.02	1.94	3.84	
108	1417586_at	BM230269	21853	timeless homolog (Drosophila)	Timeless	-3.79	1.53E-04	6.66E-03	5.78	6.84	-1.06	-2.08	
109	1417587_at	BM230269	21853	timeless homolog (Drosophila)	Timeless	-5.78	7.34E-09	1.23E-06	4.53	6.14	-1.60	-3.04	
110	1417601_at	NM_015811	50778	regulator of G-protein signaling 1	Rgs1	4.43	9.27E-06	6.50E-04	13.30	12.30	0.96	1.94	
111	1417632_at	U13836	11975	ATPase, H+ transporting, lysosomal V0 subunit A1	Atp6v0a1	3.87	1.08E-04	5.10E-03	10.40	9.56	0.86	1.82	
112	1417649_at	NM_009876	12577	cyclin-dependent kinase inhibitor 1C (P57)	Cdkn1c	14.41	0.00E+00	0.00E+00	8.63	4.93	3.70	12.99	
113	1417689_a_at	BC013542	67182	PDZK1 interacting protein 1	Pdzk1ip1	4.51	6.53E-06	4.80E-04	9.30	8.29	1.01	2.02	
114	1417694_at	NM_021356	14388	growth factor receptor bound protein 2-associated protein 1	Gab1	-3.37	7.61E-04	2.38E-02	5.87	6.81	-0.94	-1.92	
115	1417757_at	NM_021468	22249	unc-13 homolog B (C. elegans)	Unc13b	4.52	6.30E-06	4.66E-04	7.27	6.04	1.24	2.36	
116	1417779_at	NM_025636	66566	RIKEN cDNA 2310079N02 gene	2310079N02Rik	-3.49	4.79E-04	1.66E-02	8.27	9.07	-0.80	-1.75	RIKEN cDNA 2310079N02 gene (Probable UPF0334 kinase-like protein C1orf57 homolog)
117	1417793_at	NM_019440	54396	interferon inducible GTPase 2	Iigp2	-3.96	7.39E-05	3.72E-03	9.92	10.80	-0.90	-1.86	
118	1417815_a_at	NM_012032	26943	serine incorporator 3	Serinc3	-4.11	4.02E-05	2.22E-03	13.20	13.90	-0.70	-1.62	
119	1417816_s_at	NM_012032	26943	serine incorporator 3	Serinc3	-3.91	9.09E-05	4.42E-03	7.90	8.82	-0.92	-1.89	
120	1417821_at	NM_033075	110956	DNA segment, Chr 17, human D6S56E 5	D17H6S56E-5	-7.13	1.01E-12	3.78E-10	6.17	8.03	-1.86	-3.63	
121	1417822_at	NM_033075	110956	DNA segment, Chr 17, human D6S56E 5	D17H6S56E-5	-5.38	7.41E-08	9.59E-06	5.72	7.19	-1.47	-2.78	
122	1417856_at	NM_009046	19698	avian reticuloendotheliosis viral (v-rel) oncogene related B	Relb	-3.44	5.87E-04	1.94E-02	6.25	7.19	-0.93	-1.91	
123	1417864_at	NM_008828	18655 /// 668435	phosphoglycerate kinase 1 /// predicted gene, EG668435	EG668435 /// Pgk1	3.70	2.16E-04	8.88E-03	13.50	12.70	0.77	1.70	
124	1417868_a_at	NM_022325	64138	cathepsin Z	Ctsz	3.98	7.03E-05	3.57E-03	15.40	15.10	0.29	1.22	
125	1417871_at	NM_010476	15490	hydroxysteroid (17-beta) dehydrogenase 7	Hsd17b7	4.34	1.40E-05	9.20E-04	5.89	4.67	1.22	2.33	
126	1417910_at	X75483	12428	cyclin A2	Ccna2	-4.69	2.67E-06	2.24E-04	4.51	5.82	-1.31	-2.48	
127	1417932_at	NM_008360	16173	interleukin 18	Il18	-3.64	2.70E-04	1.06E-02	8.87	9.69	-0.82	-1.77	
128	1417938_at	BC003738	19362	RAD51 associated protein 1	Rad51ap1	-3.18	1.48E-03	3.99E-02	4.49	5.38	-0.89	-1.85	
129	1418003_at	NM_025427	66214	RIKEN cDNA 1190002H23 gene	1190002H23Rik	3.74	1.81E-04	7.64E-03	4.09	3.32	0.76	1.70	RIKEN cDNA 1190002H23 gene (Response gene to complement 32 protein (RGC-32))
130	1418004_a_at	NM_023056	65963	transmembrane protein 176B	Tmem176b	-6.91	4.82E-12	1.61E-09	8.21	9.81	-1.60	-3.03	RIKEN cDNA 1810009M01 gene (Transmembrane protein 176B (Protein LR8))
131	1418021_at	NM_009780	12268 /// 675521	complement component 4B (Childo blood group) /// similar to Complement C4 precursor	C4b /// LOC675521	-3.13	1.76E-03	4.55E-02	6.67	7.49	-0.82	-1.76	
132	1418025_at	NM_011498	20893	basic helix-loop-helix domain containing, class B2	Bhlhb2	8.79	0.00E+00	0.00E+00	9.84	7.84	2.00	4.01	
133	1418057_at	NM_009384	21844	T-cell lymphoma invasion and metastasis 1	Tiam1	-3.79	1.52E-04	6.63E-03	5.36	6.44	-1.08	-2.11	
134	1418069_at	NM_009695	11813	apolipoprotein C-II	Apoc2	-3.13	1.77E-03	4.57E-02	9.10	9.80	-0.70	-1.63	
135	1418074_at	AK007601	20448	ST6 (alpha-N-acetyl-neuraminyl-2,3-beta-galactosyl-1,3)-N-acetylgalactosaminide alpha-2,6-sialyltransferase 4	St6galnac4	3.91	9.32E-05	4.51E-03	8.05	7.07	0.98	1.97	
136	1418075_at	AK007601	20448	ST6 (alpha-N-acetyl-neuraminyl-2,3-beta-galactosyl-1,3)-N-acetylgalactosaminide alpha-2,6-sialyltransferase 4	St6galnac4	3.36	7.93E-04	2.46E-02	9.72	8.98	0.74	1.68	
137	1418078_at	U60330	19192	proteaseome (prosome, macropain) 28 subunit, 3	Psme3	3.43	6.12E-04	2.01E-02	8.18	7.34	0.84	1.78	
138	1418126_at	NM_013653	20304	chemokine (C-C motif) ligand 5	Ccl5	-3.13	1.73E-03	4.50E-02	12.00	12.70	-0.71	-1.64	
139	1418204_s_at	NM_019467	11629	allograft inflammatory factor 1	Aif1	-3.59	3.26E-04	1.22E-02	12.20	13.00	-0.78	-1.72	
140	1418209_a_at	NM_019410	18645	profilin 2	Pfn2	3.46	5.43E-04	1.83E-02	3.97	3.29	0.68	1.61	
141	1418252_at	NM_008812	18600 /// 638935	peptidyl arginine deiminase, type II /// similar to peptidyl arginine deiminase, type II	LOC638935 /// Padi2	3.42	6.32E-04	2.06E-02	7.00	6.05	0.95	1.93	
142	1418265_s_at	NM_008391	16363	interferon regulatory factor 2	Irf2	-3.54	4.03E-04	1.44E-02	7.91	8.74	-0.83	-1.78	
143	1418350_at	L07264	15200	heparin-binding EGF-like growth factor	Hbegf	7.83	4.66E-15	2.56E-12	5.91	3.91	2.00	4.01	
144	1418394_a_at	NM_011925	26364	CD97 antigen	Cd97	-3.16	1.58E-03	4.17E-02	8.04	8.78	-0.74	-1.67	
145	1418480_at	NM_023785	57349	pro-platelet basic protein	Ppbp	-3.13	1.73E-03	4.50E-02	3.99	4.80	-0.81	-1.75	
146	1418497_at	AF020737	14168	fibroblast growth factor 13	Fgf13	-4.02	5.88E-05	3.08E-03	7.25	8.24	-0.99	-1.98	
147	1418571_at	NM_013749	27279	tumor necrosis factor receptor superfamily, member 12a	Tnfrsf12a	4.98	6.49E-07	6.45E-05	8.21	6.96	1.24	2.37	
148	1418572_x_at	NM_013749	27279	tumor necrosis factor receptor superfamily, member 12a	Tnfrsf12a	5.11	3.28E-07	3.52E-05	8.24	6.97	1.27	2.42	
149	1418611_at	NM_013533	14788	G protein-coupled receptor 162	Gpr162	4.01	6.08E-05	3.17E-03	8.36	7.39	0.97	1.96	
150	1418634_at	NM_008714	18128	Notch gene homolog 1 (Drosophila)	Notch1	-3.26	1.13E-03	3.22E-02	7.53	8.32	-0.79	-1.72	
151	1418645_at	L07645	15109	histidine ammonia lyase	Hal	9.25	0.00E+00	0.00E+00	12.30	10.30	2.05	4.13	
152	1418711_at	BB371842	18590	platelet derived growth factor, alpha	Pdgfa	3.35	8.00E-04	2.48E-02	6.40	5.43	0.96	1.95	
153	1418718_at	BC019961	66102	chemokine (C-X-C motif) ligand 16	Cxcl16	-3.33	8.64E-04	2.65E-02	9.76	10.50	-0.75	-1.68	
154	1418774_a_at	U03434	11977	ATPase, Cu++ transporting, alpha polypeptide	Atp7a	-3.62	2.95E-04	1.13E-02	8.57	9.39	-0.82	-1.77	
155	1418776_at	NM_029509	76074	RIKEN cDNA 5830443L24 gene	5830443L24Rik	-3.28	1.06E-03	3.09E-02	5.75	6.67	-0.92	-1.89	RIKEN cDNA 5830443L24 gene (guanylate binding protein 8)
156	1418816_at	BG073376	67064	chromatin modifying protein 1B	Chmp1b	3.89	1.01E-04	4.80E-03	11.90	11.00	0.89	1.86	
157	1418817_at	BG073376	67064	chromatin modifying protein 1B	Chmp1b	3.54	3.97E-04	1.43E-02	12.80	12.00	0.79	1.73	
158	1418819_at	NM_026011	67166	ADP-ribosylation factor-like 8B	Arl8b	4.84	1.31E-06	1.19E-04	10.70	9.61	1.08	2.12	
159	1418826_at	NM_027209	69774	membrane-spanning 4-domains, subfamily A, member 6B	Ms4a6b	-3.27	1.08E-03	3.13E-02	13.40	14.00	-0.54	-1.45	
160	1418829_a_at	NM_013509	13807	enolase 2, gamma neuronal	Eno2	5.47	4.40E-08	6.08E-06	8.20	6.82	1.38	2.60	
161	1418840_at	NM_011050	18569	programmed cell death 4	Pdcd4	-3.99	6.59E-05	3.41E-03	7.21	8.20	-0.98	-1.98	
162	1418912_at	BB559706	67448	plexin domain containing 2	Plxdc2	-10.49	1.00E-25	8.52E-23	4.50	7.24	-2.74	-6.66	
163	1418930_at	NM_021274	100045000 /// 15945	chemokine (C-X-C motif) ligand 10 /// similar to Small inducible cytokine B10 precursor (CXCL10) (Interferon-gamma-induced protein CRG-2) (Gamma-IP10) (IP-10) (C7)	Cxcl10 /// LOC100045000	-8.33	8.01E-17	5.55E-14	7.23	9.26	-2.03	-4.09	
164	1418932_at	AY061760	100046232 /// 18030	nuclear factor, interleukin 3, regulated /// similar to NFIL3/E4BP4 transcription factor	LOC100046232 /// Nfil3	-3.86	1.14E-04	5.31E-03	4.86	5.96	-1.10	-2.14	
165	1418937_at	AF177196	13371	deiodinase, iodothyronine, type II	Dio2	24.14	0.00E+00	0.00E+00	7.74	3.05	4.70	25.92	
166	1418938_at	AF177196	13371	deiodinase, iodothyronine, type II	Dio2	8.02	1.11E-15	6.76E-13	4.53	2.90	1.63	3.10	
167	1418939_at	NM_008250	15284	H2.0-like homeobox	Hlx	-4.22	2.44E-05	1.47E-03	7.15	8.19	-1.04	-2.06	
168	1418992_at	NM_007972	14058	coagulation factor X	F10	4.14	3.51E-05	1.98E-03	5.52	4.39	1.13	2.19	
169	1419029_at	BM234652	50527	ERO1-like (S. cerevisiae)	Ero1l	3.48	5.07E-04	1.73E-02	9.06	8.28	0.79	1.72	1419029_at (Endoplasmic oxidoreductin-1-like protein) (ERO1-L)
170	1419030_at	BM234652	50527	ERO1-like (S. cerevisiae)	Ero1l	3.34	8.23E-04	2.54E-02	8.52	7.74	0.79	1.72	1419029_at (Endoplasmic oxidoreductin-1-like protein) (ERO1-L)
171	1419033_at	AW556821	70415	RIKEN cDNA 2610018G03 gene	2610018G03Rik	-3.98	7.00E-05	3.56E-03	4.01	5.06	-1.05	-2.07	RIKEN cDNA 2610018G03 gene (Serine/threonine-protein kinase MST4 (EC 2.7.11.1) (STE20-like kinase MST4) (MST-4))
172	1419042_at	BM239828	60440	interferon inducible GTPase 1	Iigp1	-4.71	2.42E-06	2.05E-04	9.53	10.60	-1.06	-2.09	
173	1419043_a_at	BM239828	60440	interferon inducible GTPase 1	Iigp1	-4.64	3.44E-06	2.81E-04	10.30	11.30	-1.07	-2.10	

	A	B	C	D	E	F	G	H	I	J	K	L	M
174	1419082_at	NM_011111	18788	serine (or cysteine) peptidase inhibitor, clade B, member 2	Serpinb2	-6.38	1.78E-10	4.30E-08	2.61	3.60	-0.99	-1.99	
175	1419125_at	NM_011206	19253	protein tyrosine phosphatase, non-receptor type 18	Ptpn18	-4.39	1.11E-05	7.60E-04	9.90	10.90	-1.00	-2.00	
176	1419127_at	NM_023456	109648	neuropeptide Y	Npy	9.10	0.00E+00	0.00E+00	11.80	9.74	2.05	4.13	
177	1419132_at	NM_011905	24088	toll-like receptor 2	Tlr2	-6.52	7.02E-11	1.83E-08	6.80	8.44	-1.64	-3.11	
178	1419144_at	NM_053094	93671	CD163 antigen	Cd163	-4.23	2.36E-05	1.43E-03	3.86	4.94	-1.09	-2.12	
179	1419153_at	AK017673	68026	RIKEN cDNA 2810417H13 gene	2810417H13Rik	-4.53	5.88E-06	4.40E-04	7.23	8.34	-1.11	-2.16	RIKEN cDNA 2810417H13 gene (PCNA-associated factor (p15PAF) (HCV NS5A-transactivated protein 9 homolog))
180	1419168_at	BC024684	50772	mitogen-activated protein kinase 6	Mapk6	3.24	1.21E-03	3.39E-02	7.84	7.02	0.82	1.76	
181	1419193_a_at	NM_022024	63986	glia maturation factor, gamma	Gmfg	-3.27	1.06E-03	3.10E-02	8.67	9.42	-0.74	-1.67	
182	1419194_s_at	NM_022024	63986	glia maturation factor, gamma	Gmfg	-4.63	3.72E-06	2.96E-04	9.60	10.60	-1.04	-2.06	
183	1419209_at	NM_008176	14825	chemokine (C-X-C motif) ligand 1	Cxcl1	3.49	4.78E-04	1.66E-02	6.17	5.17	1.00	2.00	
184	1419213_at	NM_019750	56441	N-acetyltransferase 6	Nat6	4.73	2.25E-06	1.93E-04	8.95	7.86	1.09	2.13	
185	1419270_a_at	AF091101	110074	deoxyuridine triphosphatase	Dut	-3.47	5.22E-04	1.78E-02	5.54	6.52	-0.98	-1.98	
186	1419282_at	U50712	100048556 /// 20293	chemokine (C-C motif) ligand 12 /// similar to monocyte chemoattractant protein-5	Ccl12 /// LOC100048556	-4.58	4.58E-06	3.55E-04	7.62	8.71	-1.09	-2.14	
187	1419289_a_at	NM_009303	20972	synaptogyrin 1	Syngr1	3.41	6.54E-04	2.12E-02	11.40	10.60	0.78	1.72	
188	1419309_at	NM_010329	14726	podoplanin	Pdpn	8.43	0.00E+00	0.00E+00	9.62	7.68	1.94	3.83	
189	1419315_at	NM_029612	98365	SLAM family member 9	Slamf9	-3.28	1.06E-03	3.09E-02	11.80	12.60	-0.75	-1.69	
190	1419321_at	NM_010172	14068	coagulation factor VII	F7	4.90	9.41E-07	8.93E-05	7.60	6.29	1.31	2.48	
191	1419401_at	AF403041	142688	ankyrin repeat and SOCS box-containing protein 13	Asb13	-3.95	7.94E-05	3.95E-03	6.49	7.53	-1.04	-2.05	
192	1419513_a_at	NM_007900	13605	ect2 oncogene	Ect2	-3.67	2.39E-04	9.64E-03	4.70	5.74	-1.04	-2.06	
193	1419515_at	NM_013710	26382	FYVE, RhoGEF and PH domain containing 2	Fgd2	-7.11	1.12E-12	4.18E-10	6.57	8.39	-1.81	-3.51	
194	1419544_at	AA987147	66335	ATPase, H+ transporting, lysosomal V1 subunit C1	Atp6v1c1	4.96	7.19E-07	7.05E-05	12.40	11.30	1.12	2.18	
195	1419545_a_at	AA987147	66335	ATPase, H+ transporting, lysosomal V1 subunit C1	Atp6v1c1	5.19	2.15E-07	2.46E-05	14.10	13.20	0.93	1.91	
196	1419546_at	AA987147	66335	ATPase, H+ transporting, lysosomal V1 subunit C1	Atp6v1c1	5.35	8.56E-08	1.10E-05	12.60	11.40	1.21	2.31	
197	1419561_at	NM_011337	20302	chemokine (C-C motif) ligand 3	Ccl3	3.98	6.81E-05	3.48E-03	12.10	11.20	0.91	1.88	
198	1419583_at	NM_007625	12418	chromobox homolog 4 (Drosophila Pc class)	Cbx4	-3.12	1.83E-03	4.67E-02	7.96	8.69	-0.73	-1.66	
199	1419594_at	NM_007800	13035	cathepsin G	Ctsg	-3.26	1.11E-03	3.18E-02	5.06	6.00	-0.93	-1.91	
200	1419601_at	AF322631	16513	potassium inwardly-rectifying channel, subfamily J, member 10	Kcnj10	-4.57	4.86E-06	3.72E-04	5.56	6.84	-1.28	-2.42	
201	1419605_at	NM_010796	17312	macrophage galactose N-acetyl-galactosamine specific lectin 1	Mgl1	-3.60	3.19E-04	1.20E-02	7.25	8.13	-0.89	-1.85	
202	1419651_at	AK009389	67149	Na+/K+ transporting ATPase interacting 1	Nkain1	4.34	1.45E-05	9.42E-04	5.16	4.04	1.13	2.18	RIKEN cDNA 2610200G18 gene (Sodium/potassium-transporting ATPase subunit beta-1-interacting protein 1 (Na(+)/K(+)-transporting ATPase subunit beta-1-interacting protein 1) (Protein FAM77C))
203	1419692_a_at	NM_008521	17001	leukotriene C4 synthase	Ltc4s	-6.90	5.31E-12	1.74E-09	3.36	5.01	-1.66	-3.15	
204	1419697_at	NM_019494	56066	chemokine (C-X-C motif) ligand 11	Cxcl11	-7.75	8.93E-15	4.62E-12	6.52	8.49	-1.98	-3.93	
205	1419706_a_at	NM_031185	83397	A kinase (PRKA) anchor protein (gravin) 12	Akap12	3.31	9.24E-04	2.79E-02	3.81	3.19	0.62	1.53	
206	1419714_at	NM_021893	60533	CD274 antigen	Cd274	4.19	2.74E-05	1.61E-03	11.20	10.20	0.95	1.93	
207	1419737_a_at	NM_010699	16828	lactate dehydrogenase A	Ldha	4.65	3.30E-06	2.71E-04	14.10	13.20	0.84	1.79	
208	1419748_at	NM_011994	26874	ATP-binding cassette, sub-family D (ALD), member 2	Abcd2	-3.41	6.61E-04	2.14E-02	5.31	6.29	-0.97	-1.96	
209	1419873_s_at	AI323359	12978	colony stimulating factor 1 receptor	Csf1r	-3.40	6.75E-04	2.17E-02	12.40	13.10	-0.72	-1.65	
210	1420008_s_at	AU017197	211652	WW, C2 and coiled-coil domain containing 1	Wwc1	6.34	2.23E-10	5.21E-08	6.36	4.61	1.75	3.37	cDNA sequence BC037006 (Protein WWC1 (WW domain-containing protein 1) (Kidney and brain protein) (KIBRA))
211	1420013_s_at	C77434	16987	lanosterol synthase	Lss	4.10	4.17E-05	2.29E-03	7.97	6.94	1.04	2.05	
212	1420088_at	AI462015	18035	nuclear factor of kappa light chain gene enhancer in B-cells inhibitor, alpha	Nfkbia	-3.13	1.74E-03	4.51E-02	10.40	11.20	-0.72	-1.65	
213	1420197_at	AI323528	17873	Growth arrest and DNA-damage-inducible 45 beta	Gadd45b	5.22	1.79E-07	2.10E-05	3.70	2.83	0.87	1.82	
214	1420336_at	NM_025629	66548	ADAMTS-like 5	Adamtsl5	3.74	1.88E-04	7.87E-03	6.54	5.47	1.07	2.09	
215	1420380_at	AF065933	20296	chemokine (C-C motif) ligand 2	Ccl2	7.67	1.69E-14	8.35E-12	11.50	9.80	1.73	3.33	
216	1420394_s_at	U05264	14727 /// 14728	glycoprotein 49 A /// leukocyte immunoglobulin-like receptor, subfamily B, member 4	Gp49a /// Lilrb4	4.78	1.79E-06	1.58E-04	14.80	14.20	0.59	1.50	
217	1420404_at	NM_019388	12524	CD86 antigen	Cd86	-3.54	4.01E-04	1.44E-02	7.31	8.18	-0.87	-1.83	
218	1420498_a_at	NM_023118	13132	disabled homolog 2 (Drosophila)	Dab2	-3.54	4.02E-04	1.44E-02	11.10	11.90	-0.84	-1.79	
219	1420499_at	NM_008102	14528	GTP cyclohydrolase 1	Gch1	3.21	1.33E-03	3.65E-02	10.90	10.20	0.73	1.65	
220	1420502_at	NM_009121	20229	spermidine/spermine N1-acetyl transferase 1	Sat1	3.38	7.25E-04	2.30E-02	14.10	13.60	0.56	1.47	
221	1420548_a_at	NM_023197	66356	RIKEN cDNA 2310008H09 gene	2310008H09Rik	4.39	1.15E-05	7.81E-04	8.35	7.28	1.07	2.09	RIKEN cDNA 2310008H09 gene (unknown)
222	1420617_at	NM_026252	67579	cytoplasmic polyadenylation element binding protein 4	Cpeb4	5.76	8.64E-09	1.43E-06	10.80	9.46	1.29	2.45	
223	1420618_at	NM_026252	67579	cytoplasmic polyadenylation element binding protein 4	Cpeb4	4.59	4.37E-06	3.41E-04	12.00	11.00	1.05	2.07	
224	1420678_a_at	NM_019583	50905	interleukin 17 receptor B	Il17rb	-3.29	9.85E-04	2.93E-02	2.86	3.35	-0.49	-1.41	
225	1420679_a_at	NM_025446	66253	androgen-induced 1	Aig1	4.15	3.28E-05	1.87E-03	9.35	8.42	0.93	1.91	
226	1420760_s_at	NM_008681	17988	N-myc downstream regulated gene 1	Ndrg1	4.38	1.18E-05	8.02E-04	11.50	10.50	1.00	2.00	
227	1420796_at	NM_009644	11624	aryl-hydrocarbon receptor repressor	Ahrr	-5.98	2.26E-09	4.35E-07	4.94	6.60	-1.66	-3.17	
228	1420811_a_at	NM_007614	12387	catenin (cadherin associated protein), beta 1	Ctnnb1	3.44	5.86E-04	1.94E-02	13.90	13.30	0.63	1.54	
229	1420818_at	NM_009192	20491	src-like adaptor	Sla	-3.64	2.69E-04	1.06E-02	5.91	6.92	-1.01	-2.02	
230	1420819_at	NM_009192	20491	src-like adaptor	Sla	-5.10	3.40E-07	3.64E-05	7.27	8.51	-1.25	-2.37	
231	1420847_a_at	NM_010207	14183	fibroblast growth factor receptor 2	Fgfr2	4.24	2.28E-05	1.39E-03	3.14	2.61	0.53	1.45	
232	1420887_a_at	NM_009743	12048	Bcl2-like 1	Bcl2l1	3.35	8.02E-04	2.48E-02	8.41	7.62	0.80	1.74	
233	1420911_a_at	NM_008594	17304	milk fat globule-EGF factor 8 protein	Mfge8	4.05	5.03E-05	2.69E-03	8.34	7.36	0.98	1.97	
234	1420965_a_at	BM120053	13803	ectodermal-neural cortex 1	Enc1	-5.00	5.87E-07	5.91E-05	9.00	10.10	-1.13	-2.18	
235	1420998_at	BG966751	104156	ets variant gene 5	Etv5	-3.71	2.04E-04	8.47E-03	6.73	7.68	-0.96	-1.94	
236	1421027_a_at	AI595932	17260	myocyte enhancer factor 2C	Mef2c	-6.51	7.31E-11	1.89E-08	9.88	11.40	-1.50	-2.83	
237	1421028_a_at	AI595932	17260	myocyte enhancer factor 2C	Mef2c	-6.39	1.70E-10	4.12E-08	8.19	9.67	-1.48	-2.78	
238	1421031_a_at	NM_023516	69573	RIKEN cDNA 2310016C08 gene	2310016C08Rik	5.94	2.84E-09	5.27E-07	8.85	7.44	1.41	2.65	RIKEN cDNA 2310016C08 gene (Hypoxia-inducible gene 2 protein)
239	1421073_a_at	NM_008965	19219	prostaglandin E receptor 4 (subtype EP4)	Ptger4	-5.99	2.10E-09	4.07E-07	4.92	6.58	-1.67	-3.18	
240	1421186_at	BB148128	12772	chemokine (C-C motif) receptor 2	Ccr2	-3.75	1.77E-04	7.47E-03	9.66	10.50	-0.84	-1.79	
241	1421187_at	BB148128	12772	chemokine (C-C motif) receptor 2	Ccr2	-3.80	1.46E-04	6.42E-03	8.24	9.12	-0.88	-1.83	
242	1421228_at	AF128193	20306	chemokine (C-C motif) ligand 7	Ccl7	6.64	3.18E-11	8.88E-09	10.70	9.16	1.49	2.80	
243	1421384_at	NM_010748	17101	lysosomal trafficking regulator	Lyst	3.18	1.47E-03	3.97E-02	7.36	6.52	0.85	1.80	
244	1421392_a_at	NM_007464	11796	baculoviral IAP repeat-containing 3	Birc3	-3.54	3.95E-04	1.42E-02	7.51	8.36	-0.86	-1.81	
245	1421408_at	NM_030691	80719	immunoglobulin superfamily, member 6	Igsf6	4.28	1.90E-05	1.19E-03	11.50	10.50	0.98	1.97	
246	1421424_a_at	NM_008486	16790	alanyl (membrane) aminopeptidase	Anpep	5.92	3.29E-09	6.05E-07	11.40	10.10	1.34	2.53	
247	1421480_a_at	NM_130895	110532	adenosine deaminase, RNA-specific, B1	Adarb1	3.78	1.55E-04	6.71E-03	3.24	2.72	0.52	1.43	
248	1421488_at	NM_013862	29809	RAB GTPase activating protein 1-like	Rabgap1l	3.22	1.30E-03	3.59E-02	4.83	4.01	0.82	1.76	
249	1421659_at	NM_013461	11549	adrenergic receptor, alpha 1a	Adra1a	3.15	1.64E-03	4.31E-02	6.44	5.54	0.90	1.87	
250	1421685_at	NM_027218	69810	C-type lectin domain family 4, member b1	Clec4b1	-5.75	8.97E-09	1.46E-06	6.85	8.30	-1.44	-2.72	
251	1421800_at	NM_011080	18686	per-hexamer repeat gene 1	Phxr1	-4.10	4.14E-05	2.28E-03	3.89	4.95	-1.06	-2.08	

	A	B	C	D	E	F	G	H	I	J	K	L	M
252	1421820_a_at	L28176	18016	neurofibromatosis 2	Nf2	3.56	3.74E-04	1.36E-02	9.42	8.63	0.79	1.73	
253	1421821_at	AF425607	16835	low density lipoprotein receptor	Ldlr	9.14	0.00E+00	0.00E+00	10.80	8.77	2.06	4.16	
254	1421846_at	BM730566	59043	WD repeat and SOCS box-containing 2	Wsb2	3.99	6.72E-05	3.46E-03	10.10	9.18	0.88	1.85	
255	1421847_at	BM730566	59043	WD repeat and SOCS box-containing 2	Wsb2	4.00	6.40E-05	3.32E-03	10.80	9.92	0.90	1.87	
256	1421924_at	M75135	20527	solute carrier family 2 (facilitated glucose transporter), member 3	Slc2a3	-3.10	1.94E-03	4.90E-02	3.24	3.84	-0.60	-1.52	
257	1421937_at	NM_011932	26377	dual adaptor for phosphotyrosine and 3-phosphoinositides 1	Dapp1	-3.59	3.26E-04	1.22E-02	5.78	6.79	-1.01	-2.01	
258	1421992_a_at	NM_010517	16010	insulin-like growth factor binding protein 4	Igfbp4	-4.93	8.25E-07	7.98E-05	7.69	8.86	-1.17	-2.25	
259	1422013_at	NM_011999	26888	C-type lectin domain family 4, member a2	Clec4a2	-3.65	2.67E-04	1.05E-02	10.30	11.10	-0.84	-1.79	
260	1422024_at	NM_008026	14247	Friend leukemia integration 1	Fli1	-3.22	1.27E-03	3.53E-02	8.58	9.32	-0.73	-1.66	
261	1422039_at	NM_023680	79202	tumor necrosis factor receptor superfamily, member 22	Tnfrsf22	3.89	1.02E-04	4.82E-03	4.85	3.88	0.97	1.96	
262	1422041_at	NM_133209	170741	paired immunoglobin-like type 2 receptor beta 1	Pilrb1	-4.20	2.71E-05	1.60E-03	5.66	6.84	-1.17	-2.25	
263	1422101_at	NM_024290	79201	tumor necrosis factor receptor superfamily, member 23	Tnfrsf23	3.25	1.15E-03	3.27E-02	5.72	4.80	0.92	1.90	
264	1422125_at	NM_008311	15559	5-hydroxytryptamine (serotonin) receptor 2B	Htr2b	6.05	1.47E-09	2.96E-07	8.31	6.78	1.52	2.88	
265	1422138_at	NM_008873	18792	plasminogen activator, urokinase	Plau	4.35	1.37E-05	9.03E-04	9.60	8.63	0.97	1.96	
266	1422139_at	NM_008873	18792	plasminogen activator, urokinase	Plau	3.78	1.55E-04	6.71E-03	10.90	10.10	0.85	1.81	
267	1422185_a_at	NM_029787	109754	cytochrome b5 reductase 3	Cyb5r3	3.26	1.10E-03	3.17E-02	11.70	10.90	0.75	1.68	
268	1422218_at	NM_011027	18439	purinergic receptor P2X, ligand-gated ion channel, 7	P2rx7	-3.47	5.26E-04	1.78E-02	4.04	4.95	-0.91	-1.88	
269	1422431_at	NM_053201	107528	melanoma antigen, family E, 1	Magee1	-3.39	6.88E-04	2.21E-02	3.23	3.89	-0.66	-1.58	
270	1422438_at	NM_010145	13849	epoxide hydrolase 1, microsomal	Ephx1	-4.36	1.27E-05	8.54E-04	7.65	8.69	-1.04	-2.06	
271	1422474_at	BM246564	18578	phosphodiesterase 4B, cAMP specific	Pde4b	-4.59	4.44E-06	3.46E-04	5.12	6.42	-1.30	-2.46	
272	1422508_at	NM_007508	11964	ATPase, H+ transporting, lysosomal V1 subunit A	Atp6v1a	3.18	1.47E-03	3.96E-02	13.40	12.80	0.65	1.57	
273	1422518_at	BM938489	12361	calcium/calmodulin-dependent serine protein kinase (MAGUK family)	Cask	-4.17	3.10E-05	1.79E-03	5.46	6.63	-1.17	-2.26	
274	1422527_at	NM_010386	14998	histocompatibility 2, class II, locus DMa	H2-DMa	-4.03	5.68E-05	3.00E-03	8.78	9.69	-0.91	-1.88	
275	1422528_a_at	M58566	12192	zinc finger protein 36, C3H type-like 1	Zfp36l1	-5.13	2.97E-07	3.23E-05	8.78	9.94	-1.16	-2.23	
276	1422533_at	NM_020010	13121	cytochrome P450, family 51	Cyp51	6.01	1.91E-09	3.73E-07	6.64	4.96	1.68	3.21	
277	1422537_a_at	NM_010496	15902	inhibitor of DNA binding 2	Id2	6.70	2.02E-11	5.87E-09	11.60	10.10	1.52	2.86	
278	1422542_at	NM_011823	23890	G protein-coupled receptor 34	Gpr34	-5.76	8.57E-09	1.42E-06	7.38	8.77	-1.39	-2.63	
279	1422562_at	NM_019662	56437	Ras-related associated with diabetes	Rrad	-3.51	4.44E-04	1.56E-02	4.23	5.18	-0.95	-1.94	
280	1422592_at	NM_008729	100045979 /// 18163	catenin (cadherin associated protein), delta 2 /// similar to arm-repeat protein NPRAP/neurojungin	Ctnnd2 /// LOC100045979	-6.19	6.09E-10	1.33E-07	4.79	6.50	-1.72	-3.29	
281	1422593_at	NM_009681	11777	adaptor-related protein complex 3, sigma 1 subunit	Ap3s1	4.18	2.87E-05	1.66E-03	12.80	11.90	0.94	1.91	
282	1422603_at	BC005569	58809	ribonuclease, RNase A family 4	Rnase4	-4.22	2.49E-05	1.49E-03	11.70	12.70	-0.97	-1.95	
283	1422612_at	NM_013820	15277	hexokinase 2	Hk2	3.63	2.80E-04	1.09E-02	9.93	9.13	0.81	1.75	
284	1422631_at	BE989096	11622	aryl-hydrocarbon receptor	Ahr	-5.16	2.48E-07	2.78E-05	4.74	6.19	-1.45	-2.73	
285	1422645_at	AJ306425	15216	hemochromatosis	Hfe	-4.90	9.68E-07	9.10E-05	8.11	9.25	-1.14	-2.20	
286	1422648_at	BF533509	11988	solute carrier family 7 (cationic amino acid transporter, y+ system), member 2	Slc7a2	7.78	7.55E-15	3.95E-12	5.89	3.90	1.99	3.97	
287	1422702_at	BE626090	54375	antizyme inhibitor 1	Azin1	4.22	2.43E-05	1.46E-03	12.00	11.00	0.97	1.96	
288	1422706_at	AV370981	65112	transmembrane, prostate androgen induced RNA	Tmepai	-3.18	1.48E-03	3.97E-02	5.43	6.34	-0.91	-1.88	
289	1422707_at	BB205102	30955	phosphoinositide-3-kinase, catalytic, gamma polypeptide	Pik3cg	-3.29	9.98E-04	2.96E-02	7.24	8.05	-0.81	-1.76	
290	1422708_at	BB205102	30955	phosphoinositide-3-kinase, catalytic, gamma polypeptide	Pik3cg	-4.77	1.88E-06	1.65E-04	3.92	5.17	-1.25	-2.38	
291	1422751_at	NM_011599	21885	transducin-like enhancer of split 1, homolog of Drosophila E(spl)	Tle1	-3.11	1.89E-03	4.79E-02	5.48	6.36	-0.89	-1.85	
292	1422754_at	NM_021883	21916	tropomodulin 1	Tmod1	5.09	3.54E-07	3.76E-05	8.69	7.48	1.21	2.31	
293	1422814_at	NM_009791	12316	asp (abnormal spindle)-like, microcephaly associated (Drosophila)	Aspm	-3.19	1.42E-03	3.87E-02	2.82	3.28	-0.45	-1.37	
294	1422820_at	NM_010719	16890	lipase, hormone sensitive	Lipe	-4.03	5.48E-05	2.90E-03	5.52	6.65	-1.14	-2.20	
295	1422851_at	X58380	15364	high mobility group AT-hook 2	Hmga2	5.05	4.50E-07	4.69E-05	10.10	8.98	1.12	2.17	
296	1422869_at	NM_008587	17289	c-mer proto-oncogene tyrosine kinase	Mertk	-3.77	1.60E-04	6.91E-03	8.54	9.40	-0.86	-1.81	
297	1422903_at	NM_010745	17084	lymphocyte antigen 86	Ly86	-4.06	4.89E-05	2.63E-03	12.80	13.60	-0.77	-1.70	
298	1422953_at	NM_008039	14289	formyl peptide receptor, related sequence 2	Fpr-rs2	-6.79	1.14E-11	3.48E-09	7.56	9.18	-1.62	-3.08	
299	1422957_at	NM_009914	12771	chemokine (C-C motif) receptor 3	Ccr3	-5.18	2.18E-07	2.49E-05	3.93	5.30	-1.36	-2.58	
300	1422966_a_at	BB810450	22042	transferrin receptor	Tfrc	3.24	1.20E-03	3.38E-02	8.58	7.83	0.76	1.69	
301	1422967_a_at	BB810450	22042	transferrin receptor	Tfrc	3.28	1.05E-03	3.08E-02	7.17	6.28	0.89	1.86	
302	1423017_a_at	NM_031167	16181	interleukin 1 receptor antagonist	Il1rn	9.27	0.00E+00	0.00E+00	12.10	10.00	2.07	4.19	
303	1423048_a_at	BB400304	54473	toll interacting protein	Tollip	3.22	1.29E-03	3.57E-02	8.53	7.78	0.75	1.69	
304	1423049_a_at	AK002271	22003	tropomyosin 1, alpha	Tpm1	4.19	2.75E-05	1.62E-03	8.25	7.22	1.03	2.04	
305	1423064_at	BB795491	13435	DNA methyltransferase 3A	Dnmt3a	9.52	0.00E+00	0.00E+00	10.90	8.79	2.14	4.42	
306	1423065_at	BB795491	13435	DNA methyltransferase 3A	Dnmt3a	8.37	0.00E+00	0.00E+00	8.12	5.91	2.22	4.64	
307	1423066_at	BB795491	13435	DNA methyltransferase 3A	Dnmt3a	11.27	0.00E+00	0.00E+00	12.10	9.56	2.51	5.70	
308	1423078_a_at	AK005441	66234	sterol-C4-methyl oxidase-like	Sc4mol	5.27	1.34E-07	1.61E-05	11.00	9.84	1.19	2.28	
309	1423100_at	AV026617	14281	FBJ osteosarcoma oncogene	Fos	-4.00	6.37E-05	3.30E-03	11.20	12.20	-0.95	-1.93	
310	1423134_at	BG076340	80291	Rab interacting lysosomal protein-like 2	Rilpl2	7.25	4.21E-13	1.69E-10	12.90	11.30	1.59	3.02	cDNA sequence BC003324 (Histone-lysine N-methyltransferase, H4 lysine-20 specific (EC 2.1.1.43) (Histone H4-K20 methyltransferase))
311	1423153_x_at	AI987976	100048018 /// 12628	complement component factor h /// similar to complement component factor H	Cfh /// LOC100048018	-5.58	2.41E-08	3.65E-06	6.46	7.90	-1.45	-2.73	
312	1423160_at	BQ044290	114715	sprouty protein with EVH-1 domain 1, related sequence	Spred1	-4.41	1.01E-05	6.98E-04	9.44	10.40	-0.99	-1.99	
313	1423161_s_at	BQ044290	114715	sprouty protein with EVH-1 domain 1, related sequence	Spred1	-3.62	2.93E-04	1.13E-02	9.11	9.92	-0.81	-1.76	
314	1423162_s_at	BQ044290	114715	sprouty protein with EVH-1 domain 1, related sequence	Spred1	-3.70	2.14E-04	8.82E-03	7.76	8.64	-0.88	-1.84	
315	1423164_at	AK010724	664862 /// 83924	G protein-coupled receptor 137B /// G protein-coupled receptor 137B, pseudogene	Gpr137b /// Gpr137b-ps	5.40	6.75E-08	8.84E-06	9.17	7.94	1.23	2.35	
316	1423182_at	AK004668	57916	tumor necrosis factor receptor superfamily, member 13b	Tnfrsf13b	-3.32	9.12E-04	2.75E-02	8.64	9.39	-0.75	-1.68	
317	1423233_at	BB831146	12609	CCAAT/enhancer binding protein (C/EBP), delta	Cebpd	-3.63	2.82E-04	1.09E-02	5.58	6.61	-1.03	-2.04	
318	1423255_at	BI154058	66290	ATPase, H+ transporting, lysosomal V1 subunit G1	Atp6v1g1	3.96	7.54E-05	3.78E-03	13.00	12.10	0.88	1.84	
319	1423256_a_at	BI154058	100042604 /// 100044530 /// 638149 /// 66290 /// 666030	ATPase, H+ transporting, lysosomal V1 subunit G1 /// similar to ATPase, H+ transporting, V1 subunit G isoform 1 /// predicted gene, EG666030 /// similar to vacuolar H+ ATPase G1	Atp6v1g1 /// EG666030 /// LOC100042604 /// LOC100044530 /// LOC638149	3.41	6.49E-04	2.11E-02	14.10	13.60	0.56	1.48	
320	1423296_at	AK003436	57296	proteasome (prosome, macropain) 26S subunit, non-ATPase, 8	Psmd8	3.53	4.19E-04	1.49E-02	12.90	12.20	0.78	1.72	
321	1423345_at	AV286991	13244	degenerative spermatocyte homolog 1 (Drosophila)	Degs1	3.48	5.08E-04	1.74E-02	12.40	11.60	0.80	1.74	
322	1423350_at	AA510713	56468	suppressor of cytokine signaling 5	Socs5	-3.41	6.44E-04	2.10E-02	6.29	7.22	-0.92	-1.89	
323	1423413_at	AI987929	17988	N-myc downstream regulated gene 1	Ndrg1	4.83	1.38E-06	1.25E-04	10.90	9.84	1.09	2.13	
324	1423414_at	BB520073	19224	prostaglandin-endoperoxide synthase 1	Ptgs1	-3.57	3.56E-04	1.31E-02	5.28	6.30	-1.02	-2.03	
325	1423418_at	BI247584	110196	farnesyl diphosphate synthetase	Fdps	7.65	2.00E-14	9.78E-12	8.99	7.15	1.84	3.59	
326	1423449_a_at	BM234779	60595	actinin alpha 4	Actn4	3.21	1.33E-03	3.65E-02	11.40	10.70	0.74	1.67	
327	1423475_at	BB278418	94219	cyclin M2	Cnnm2	-3.15	1.65E-03	4.34E-02	5.01	5.92	-0.90	-1.87	
328	1423488_at	BC021914	67468	monocyte to macrophage differentiation-associated	Mmd	-4.00	6.24E-05	3.25E-03	7.98	8.92	-0.93	-1.91	
329	1423489_at	BC021914	100047565 /// 67468	monocyte to macrophage differentiation-associated /// similar to monocyte to macrophage differentiation-associated	LOC100047565 /// Mmd	-5.49	4.06E-08	5.71E-06	7.30	8.64	-1.34	-2.53	
330	1423569_at	AW108522	67092	glycine amidinotransferase (L-arginine:glycine amidinotransferase)	Gatm	-5.10	3.37E-07	3.61E-05	9.28	10.40	-1.15	-2.22	
331	1423593_a_at	AK004947	12978	colony stimulating factor 1 receptor	Csf1r	-3.80	1.44E-04	6.35E-03	12.10	12.90	-0.84	-1.79	
332	1423635_at	AV239587	12156	bone morphogenetic protein 2	Bmp2	-6.99	2.72E-12	9.57E-10	4.04	5.90	-1.86	-3.63	
333	1423721_at	M22479	22003	tropomyosin 1, alpha	Tpm1	3.32	9.00E-04	2.73E-02	9.37	8.63	0.74	1.67	
334	1423726_at	BC025496	26949	vesicle amine transport protein 1 homolog (T californica)	Vat1	4.13	3.66E-05	2.04E-03	10.80	9.85	0.93	1.90	
335	1423756_s_at	BC019836	16010	insulin-like growth factor binding protein 4	Igfbp4	-6.93	4.34E-12	1.47E-09	8.38	9.97	-1.59	-3.01	
336	1423757_x_at	BC019836	16010	insulin-like growth factor binding protein 4	Igfbp4	-5.88	4.22E-09	7.43E-07	6.63	8.13	-1.50	-2.83	
337	1423775_s_at	BC005475	233406	protein regulator of cytokinesis 1	Prc1	-3.80	1.45E-04	6.41E-03	4.27	5.30	-1.04	-2.05	
338	1423804_a_at	BC004801	319554	isopentenyl-diphosphate delta isomerase	Idi1	5.83	5.58E-09	9.59E-07	8.58	7.16	1.42	2.67	
339	1423805_at	BC006588	13132	disabled homolog 2 (Drosophila)	Dab2	-5.69	1.28E-08	2.05E-06	9.45	10.70	-1.29	-2.44	
340	1423809_at	BC004617	106795	transcription factor 19	Tcf19	-3.63	2.80E-04	1.09E-02	5.95	6.95	-1.01	-2.01	
341	1423847_at	BC025460	68298	non-SMC condensin I complex, subunit D2	Ncapd2	-3.09	1.99E-03	4.97E-02	3.42	4.08	-0.66	-1.58	RIKEN cDNA 2810406C15 gene (Condensin complex subunit 1 (Non-SMC condensin I complex subunit D2) (Chromosome condensation-related SMC-associated protein 1))

	A	B	C	D	E	F	G	H	I	J	K	L	M
342	1423909_at	BC010831	66058	transmembrane protein 176A	Tmem176a	-5.50	3.75E-08	5.32E-06	6.65	8.06	-1.41	-2.65	RIKEN cDNA 0610011I04 gene (Transmembrane protein 176A (Kidney-expressed gene 2 protein) (Gene signature 188))
343	1423954_at	K02782	12266	complement component 3	C3	-5.44	5.25E-08	7.05E-06	8.47	9.72	-1.24	-2.37	
344	1423989_at	BC025567	70381	RIKEN cDNA 2210010N04 gene	2210010N04Rik	4.30	1.72E-05	1.09E-03	9.30	8.34	0.97	1.95	RIKEN cDNA 2210010N04 gene (Class B basic helix-loop-helix protein 8 (bHLHB8) (Muscle, intestine and stomach expression 1) (MIST-1))
345	1424032_at	BC021548	74096	hydrogen voltage-gated channel 1	Hvcn1	3.75	1.76E-04	7.47E-03	9.34	8.50	0.84	1.79	RIKEN cDNA 0610039P13 gene (hydrogen voltage-gated channel 1)
346	1424046_at	AF002823	12235	budding uninhibited by benzimidazoles 1 homolog (S. cerevisiae)	Bub1	-4.14	3.42E-05	1.94E-03	4.26	5.39	-1.13	-2.19	
347	1424067_at	BC008626	15894	intercellular adhesion molecule	Icam1	-3.54	3.96E-04	1.43E-02	8.39	9.20	-0.81	-1.75	
348	1424089_a_at	U16321	21413	transcription factor 4	Tcf4	-3.86	1.14E-04	5.31E-03	9.36	10.20	-0.86	-1.82	
349	1424105_a_at	AF069051	30939	pituitary tumor-transforming 1	Pttg1	-4.72	2.32E-06	1.98E-04	7.90	9.00	-1.11	-2.16	
350	1424128_x_at	BC003261	20877	aurora kinase B	Aurkb	-4.44	9.04E-06	6.36E-04	4.02	5.20	-1.18	-2.26	
351	1424155_at	BC002148	11770	fatty acid binding protein 4, adipocyte	Fabp4	11.51	0.00E+00	0.00E+00	4.72	2.45	2.28	4.84	
352	1424162_at	BC027353	72169	tripartite motif protein 29	Trim29	4.78	1.74E-06	1.54E-04	5.05	3.85	1.20	2.30	
353	1424208_at	BC011193	19219	prostaglandin E receptor 4 (subtype EP4)	Ptger4	-6.30	2.98E-10	6.89E-08	8.83	10.30	-1.43	-2.69	
354	1424211_at	BC011293	70556	solute carrier family 25, member 33	Slc25a33	3.29	1.00E-03	2.97E-02	7.29	6.40	0.89	1.85	RIKEN cDNA 5730438N18 gene (Solute carrier family 25 member 33)
355	1424246_a_at	BC003808	21753	testis derived transcript	Tes	4.20	2.63E-05	1.56E-03	7.95	6.88	1.07	2.10	
356	1424343_a_at	BM200591	13664	eukaryotic translation initiation factor 1A	Eif1a	3.12	1.82E-03	4.65E-02	10.70	9.98	0.70	1.62	
357	1424354_at	BC020080	68487	transmembrane protein 140	Tmem140	3.12	1.84E-03	4.69E-02	11.20	10.50	0.71	1.64	RIKEN cDNA 1110007F12 gene (Transmembrane protein 140)
358	1424393_s_at	BC026584	76187	alcohol dehydrogenase, iron containing, 1	Adhfe1	3.14	1.68E-03	4.40E-02	4.22	3.53	0.69	1.61	
359	1424411_at	BB208266	407243	transmembrane protein 189	Tmem189	3.46	5.40E-04	1.83E-02	10.70	9.90	0.78	1.71	expressed sequence AI840826 (ubiquitin-conjugating enzyme variant Kua)
360	1424413_at	BE650508	70155	opioid growth factor receptor-like 1	Ogfrl1	-3.63	2.81E-04	1.09E-02	7.25	8.14	-0.90	-1.86	
361	1424443_at	AV378394	107769 /// 29877	hepatoma-derived growth factor, related protein 3 /// transmembrane 6 superfamily member 1	Hdgfrp3 /// Tm6sf1	-3.83	1.27E-04	5.79E-03	11.10	12.00	-0.91	-1.88	
362	1424444_a_at	BC019834	72244	RIKEN cDNA 1600014C10 gene	1600014C10Rik	-3.65	2.61E-04	1.03E-02	7.79	8.65	-0.86	-1.82	RIKEN cDNA 1600014C10 gene (Uncharacterized protein C19orf12 homolog)
363	1424453_at	BC018313	13026	phosphate cytidylyltransferase 1, choline, alpha isoform	Pcyt1a	3.64	2.68E-04	1.05E-02	8.81	7.97	0.84	1.79	
364	1424470_a_at	BC020532	223864	Rap guanine nucleotide exchange factor (GEF) 3	Rapgef3	6.19	6.08E-10	1.33E-07	6.68	4.95	1.73	3.31	
365	1424471_at	BC020532	223864	Rap guanine nucleotide exchange factor (GEF) 3	Rapgef3	7.88	3.11E-15	1.79E-12	7.25	5.09	2.16	4.47	
366	1424713_at	AY061807	75600	calmodulin-like 4	Calml4	-3.95	7.94E-05	3.95E-03	3.91	4.93	-1.02	-2.03	
367	1424783_a_at	BC019434	22236 /// 394430 /// 394432 /// 394433 /// 394434 /// 394435 /// 394436 /// 94284	UDP glucuronosyltransferase 1 family, polypeptide A2 /// UDP glucuronosyltransferase family, polypeptide A6A /// UDP glycosyltransferase 1 family, polypeptide A10 /// UDP glucuronosyltransferase 1 family, polypeptide A7C /// UDP glucuronosyltransferase 1 family	Ugt1a1 /// Ugt1a10 /// Ugt1a2 /// Ugt1a5 /// Ugt1a6a /// Ugt1a6b /// Ugt1a7c /// Ugt1a9	-3.72	2.02E-04	8.37E-03	9.16	9.99	-0.83	-1.78	
368	1424842_a_at	BC025502	231532	Rho GTPase activating protein 24	Arhgap24	7.82	5.33E-15	2.89E-12	8.15	6.10	2.05	4.15	
369	1424852_at	BB280300	17260	myocyte enhancer factor 2C	Mef2c	-6.68	2.44E-11	6.92E-09	8.73	10.30	-1.52	-2.87	
370	1424863_a_at	AF170301	15258	homeodomain interacting protein kinase 2	Hipk2	3.20	1.39E-03	3.79E-02	7.95	7.15	0.80	1.74	
371	1424882_a_at	BC011230	70021	5'-nucleotidase domain containing 2	Nt5dc2	-4.35	1.37E-05	9.03E-04	6.24	7.40	-1.16	-2.24	RIKEN cDNA 2510015F01 gene (Stabilin-1 precursor (Fasciclin, EGF-like, laminin-type EGF-like and link domain-containing scavenger receptor 1) (FEEL-1))
372	1424896_at	BC026975	64450	G protein-coupled receptor 85	Gpr85	-6.55	5.62E-11	1.48E-08	5.20	7.00	-1.80	-3.49	
373	1424911_a_at	BC024932	69032	lysozyme-like 4	Lyzl4	-3.29	1.01E-03	2.98E-02	8.70	9.44	-0.74	-1.68	
374	1424913_at	AK009800	217732	RIKEN cDNA 2310044G17 gene	2310044G17Rik	7.35	1.92E-13	8.07E-11	9.83	8.18	1.65	3.14	RIKEN cDNA 2310044G17 gene (Uncharacterized protein KIAA1737)
375	1424914_at	AK009800	217732	RIKEN cDNA 2310044G17 gene	2310044G17Rik	6.86	6.94E-12	2.22E-09	8.66	6.97	1.69	3.22	RIKEN cDNA 2310044G17 gene (Uncharacterized protein KIAA1737)
376	1424915_s_at	AK009800	217732	RIKEN cDNA 2310044G17 gene	2310044G17Rik	6.39	1.70E-10	4.12E-08	11.00	9.58	1.44	2.71	RIKEN cDNA 2310044G17 gene (Uncharacterized protein KIAA1737)
377	1424923_at	BC002065	20715	serine (or cysteine) peptidase inhibitor, clade A, member 3G	Serpina3g	-3.36	7.92E-04	2.46E-02	5.97	6.90	-0.93	-1.91	
378	1424930_s_at	BC016600	213956	expressed sequence AW544981	AW544981	4.26	2.00E-05	1.25E-03	7.02	5.83	1.19	2.28	1419029_at (Endoplasmic oxidoreductin-1-like protein) (ERO1-L)
379	1424976_at	BC021307	228543	ras homolog gene family, member V	Rhov	8.17	4.44E-16	2.90E-13	9.49	7.60	1.89	3.70	
380	1424986_s_at	AV338062	50754	F-box and WD-40 domain protein 7, archipelago homolog (Drosophila)	Fbxw7	3.45	5.66E-04	1.89E-02	7.51	6.60	0.91	1.88	
381	1424998_at	AF396935	52614	EGF-like module containing, mucin-like, hormone receptor-like sequence 4	Emr4	-9.96	2.20E-23	1.80E-20	3.18	5.56	-2.37	-5.18	
382	1425002_at	BC010462	209588	secreted and transmembrane 1A	Sectm1a	-4.90	9.66E-07	9.10E-05	3.77	5.02	-1.25	-2.38	cDNA sequence BC010462 (Secreted and transmembrane protein 1A precursor)
383	1425108_a_at	BC004728	207818	cDNA sequence BC004728	BC004728	-4.15	3.26E-05	1.86E-03	6.92	7.97	-1.05	-2.07	cDNA sequence BC004728 (DAZ-associated protein 2 (Deleted in azoospermia-associated protein 2))
384	1425125_at	AF356506	18302	oncoprotein induced transcript 3	Oit3	3.42	6.27E-04	2.05E-02	7.98	7.13	0.85	1.81	
385	1425144_at	BC013660	16613	kallikrein 1-related peptidase b11	Klk1b11	-3.13	1.76E-03	4.55E-02	5.38	6.28	-0.90	-1.86	
386	1425214_at	BC027331	233571	pyrimidinergic receptor P2Y, G-protein coupled, 6	P2ry6	-4.24	2.27E-05	1.39E-03	10.20	11.20	-0.97	-1.96	
387	1425294_at	BC024587	74748	SLAM family member 8	Slamf8	9.38	0.00E+00	0.00E+00	9.22	6.93	2.29	4.88	
388	1425329_a_at	AF332060	109754	cytochrome b5 reductase 3	Cyb5r3	4.01	6.07E-05	3.17E-03	10.40	9.50	0.89	1.86	
389	1425406_at	BC006623	26888	C-type lectin domain family 4, member a2	Clec4a2	-4.86	1.20E-06	1.10E-04	8.42	9.54	-1.11	-2.16	
390	1425407_s_at	BC006623	26888 /// 69810	C-type lectin domain family 4, member a2 /// C-type lectin domain family 4, member b1	Clec4a2 /// Clec4b1	-4.16	3.23E-05	1.85E-03	10.80	11.70	-0.98	-1.97	
391	1425436_x_at	U49865	16634 /// 16640	killer cell lectin-like receptor, subfamily A, member 3 /// killer cell lectin-like receptor subfamily A, member 9	Klra3 /// Klra9	3.68	2.33E-04	9.43E-03	9.89	9.07	0.82	1.76	
392	1425476_at	BM250666	12830	collagen, type IV, alpha 5	Col4a5	-3.25	1.15E-03	3.27E-02	4.54	5.45	-0.91	-1.88	
393	1425514_at	M60651	18708	phosphatidylinositol 3-kinase, regulatory subunit, polypeptide 1 (p85 alpha)	Pik3r1	-3.16	1.59E-03	4.21E-02	8.23	8.96	-0.73	-1.66	
394	1425536_at	D29797	20908	syntaxin 3	Stx3	4.05	5.22E-05	2.77E-03	10.60	9.65	0.90	1.87	
395	1425576_at	BC018218	229709	S-adenosylhomocysteine hydrolase-like 1	Ahcyl1	3.44	5.83E-04	1.94E-02	8.89	8.11	0.79	1.72	
396	1425603_at	BC006049	66058	transmembrane protein 176A	Tmem176a	-6.87	6.26E-12	2.03E-09	3.61	5.35	-1.74	-3.33	RIKEN cDNA 0610011I04 gene (Transmembrane protein 176A (Kidney-expressed gene 2 protein) (Gene signature 188))
397	1425627_x_at	J03952	100043965 /// 14862 /// 433943 /// 436518 /// 674086	glutathione S-transferase, mu 1 /// similar to Glutathione S-transferase Mu 1 (GST class-mu 1 (Glutathione S-transferase GT8.7) (pmGT10) (GST 1-1)	Gstm1 /// LOC100043965 /// LOC433943 /// LOC436518 /// LOC674086	4.02	5.93E-05	3.10E-03	6.81	5.68	1.13	2.19	
398	1425658_at	AY083458	235505	CD109 antigen	Cd109	5.80	6.50E-09	1.10E-06	8.96	7.60	1.36	2.56	
399	1425663_at	M57525	16181	interleukin 1 receptor antagonist	Il1rn	8.74	0.00E+00	0.00E+00	9.74	7.74	2.00	4.00	
400	1425686_at	BE284491	12633	CASP8 and FADD-like apoptosis regulator	Cflar	3.17	1.54E-03	4.10E-02	8.91	8.19	0.72	1.65	
401	1425784_a_at	D78264	56177	olfactomedin 1	Olfm1	-3.52	4.33E-04	1.53E-02	5.85	6.83	-0.98	-1.97	
402	1425809_at	BC018558	11770	fatty acid binding protein 4, adipocyte	Fabp4	10.39	0.00E+00	0.00E+00	5.60	3.22	2.38	5.20	
403	1425837_a_at	AF199491	100047134 /// 12457	CCR4 carbon catabolite repression 4-like (S. cerevisiae) /// similar to carbon catabolite repression 4 protein homolog	Ccrn4l /// LOC100047134	-3.80	1.43E-04	6.33E-03	7.26	8.19	-0.94	-1.91	
404	1425838_at	BC021814	11981	ATPase, class II, type 9A	Atp9a	6.49	8.49E-11	2.15E-08	2.65	2.12	0.52	1.44	
405	1425869_a_at	BC010403	19165	presenilin 2	Psen2	3.14	1.68E-03	4.40E-02	10.70	10.00	0.71	1.63	
406	1425946_at	BC019413	68312	glutathione S-transferase, mu 7	Gstm7	3.76	1.67E-04	7.15E-03	2.58	2.27	0.31	1.24	
407	1426002_a_at	AB018574	12545	cell division cycle 7 (S. cerevisiae)	Cdc7	3.22	1.28E-03	3.54E-02	4.47	3.71	0.75	1.69	
408	1426008_a_at	M62838	11988	solute carrier family 7 (cationic amino acid transporter, y+ system), member 2	Slc7a2	7.81	5.77E-15	3.10E-12	5.68	3.73	1.95	3.86	
409	1426081_a_at	AF177197	13371	deiodinase, iodothyronine, type II	Dio2	12.86	0.00E+00	0.00E+00	6.77	3.70	3.06	8.35	
410	1426112_a_at	BC003824	12517	CD72 antigen	Cd72	-4.28	1.86E-05	1.17E-03	10.60	11.60	-1.00	-2.00	
411	1426169_a_at	AF257136	56743	linker for activation of T cells family, member 2	Lat2	5.92	3.31E-09	6.06E-07	12.20	10.80	1.34	2.53	
412	1426172_a_at	AF374470	170786	CD209a antigen	Cd209a	-3.62	2.94E-04	1.13E-02	3.20	3.90	-0.71	-1.63	
413	1426242_at	U37500	20020	polymerase (RNA) II (DNA directed) polypeptide A	Polr2a	3.25	1.15E-03	3.27E-02	9.30	8.57	0.73	1.66	
414	1426246_at	Z25469	19128	protein S (alpha)	Pros1	-4.06	4.96E-05	2.66E-03	6.53	7.59	-1.06	-2.09	
415	1426258_at	BI648081	100047936 /// 20660	sortilin-related receptor, LDLR class A repeats-containing /// similar to sortilin-related receptor LDLR class A repeats-containing	LOC100047936 /// Sorl1	-3.10	1.93E-03	4.86E-02	5.99	6.85	-0.86	-1.82	

	A	B	C	D	E	F	G	H	I	J	K	L	M
416	1426261_s_at	D87867	22236 /// 394430 /// 394432 /// 394433 /// 394434 /// 394435 /// 394436 ///	UDP glucuronosyltransferase 1 family, polypeptide A2 /// UDP glucuronosyltransferase 1 family, polypeptide A6A /// UDP glycosyltransferase 1 family, polypeptide A10 /// UDP	Ugt1a1 /// Ugt1a10 /// Ugt1a2 /// Ugt1a5 /// Ugt1a6a /// Ugt1a6b /// Ugt1a7c /// Ugt1a9	-4.12	3.82E-05	2.12E-03	9.30	10.20	-0.92	-1.90	
417	1426278_at	AY090098	76933	interferon, alpha-inducible protein 27	Ifi27	-3.12	1.78E-03	4.58E-02	14.40	14.80	-0.35	-1.27	
418	1426288_at	AF247637	228357	low density lipoprotein receptor-related protein 4	Lrp4	4.10	4.17E-05	2.29E-03	5.06	4.00	1.05	2.08	
419	1426300_at	U95030	11658	activated leukocyte cell adhesion molecule	Alcam	4.13	3.70E-05	2.07E-03	11.60	10.60	0.94	1.92	
420	1426301_at	U95030	11658	activated leukocyte cell adhesion molecule	Alcam	4.53	5.97E-06	4.45E-04	11.20	10.20	1.03	2.04	
421	1426366_at	AW553784	239528	eukaryotic translation initiation factor 2C, 2	Eif2c2	6.02	1.69E-09	3.36E-07	9.86	8.52	1.34	2.54	
422	1426389_at	BG071931	227541	calcium/calmodulin-dependent protein kinase ID	Camk1d	-3.77	1.63E-04	6.99E-03	7.26	8.18	-0.93	-1.90	
423	1426403_at	BG801851	226977	ARP1 actin-related protein 1 homolog B (yeast)	Actr1b	3.41	6.56E-04	2.13E-02	7.23	6.31	0.93	1.90	
424	1426406_at	BB787289	67956	SET domain containing (lysine methyltransferase) 8	Setd8	4.22	2.48E-05	1.48E-03	8.14	7.09	1.05	2.07	RIKEN cDNA 2410195B05 gene (Histone-lysine N-methyltransferase, H4 lysine-20 specific (EC 2.1.1.43) (Histone H4-K20 methyltransferase))
425	1426441_at	BG065264	18174	solute carrier family 11 (proton-coupled divalent metal ion transporters), member 2	Slc11a2	3.85	1.16E-04	5.36E-03	6.95	5.87	1.08	2.11	
426	1426495_at	BQ031311	72425	RIKEN cDNA 2410042D21 gene	2410042D21Rik	3.45	5.58E-04	1.87E-02	8.32	7.50	0.83	1.78	RIKEN cDNA 2410042D21 gene (Uncharacterized protein C15orf29 homolog)
427	1426505_at	AI122415	216984	ecotropic viral integration site 2b	Evi2b	-3.55	3.82E-04	1.38E-02	10.50	11.40	-0.82	-1.77	
428	1426526_s_at	AU017520	12659	oviductal glycoprotein 1	Ovgp1	3.81	1.41E-04	6.26E-03	3.01	2.56	0.44	1.36	
429	1426554_a_at	BI407347	18648	phosphoglycerate mutase 1	Pgam1	5.42	5.89E-08	7.78E-06	14.20	13.20	0.96	1.95	
430	1426600_at	BM209618	20525	solute carrier family 2 (facilitated glucose transporter), member 1	Slc2a1	3.78	1.59E-04	6.85E-03	8.79	7.92	0.87	1.83	
431	1426607_at	BG068672	633640	predicted gene, EG633640	EG633640	4.32	1.54E-05	9.89E-04	6.79	5.57	1.22	2.33	1426607_at (Unknown)
432	1426620_at	BB549997	98388	carbohydrate sulfotransferase 10	Chst10	4.05	5.21E-05	2.77E-03	7.32	6.22	1.10	2.14	
433	1426645_at	AU079047	15519	heat shock protein 90kDa alpha (cytosolic), class A member 1	Hsp90aa1	3.41	6.53E-04	2.12E-02	11.00	10.30	0.77	1.71	RIKEN cDNA 4932703K07 gene (Heat shock protein HSP 90-alpha (HSP 86))
434	1426656_at	BM118729	75007	RIKEN cDNA 4930504E06 gene	4930504E06Rik	3.51	4.48E-04	1.57E-02	10.00	9.25	0.78	1.72	RIKEN cDNA 4930504E06 gene (Annexin A9 (Annexin-9) (Annexin-31) (Annexin XXXI))
435	1426663_s_at	BC024519	212980	solute carrier family 45, member 3	Slc45a3	6.36	1.97E-10	4.66E-08	5.18	3.63	1.55	2.92	
436	1426664_x_at	BC024519	212980	solute carrier family 45, member 3	Slc45a3	3.67	2.44E-04	9.81E-03	5.99	4.94	1.05	2.07	
437	1426724_at	AI314104	100047856 /// 71994	calponin 3, acidic /// similar to calponin 3, acidic	Cnn3 /// LOC100047856	3.44	5.84E-04	1.94E-02	2.94	2.55	0.39	1.31	
438	1426734_at	BB008324	224093	cDNA sequence BC022623	BC022623	-5.25	1.54E-07	1.83E-05	4.07	5.48	-1.41	-2.65	cDNA sequence BC022623 (Protein FAM43A)
439	1426744_at	BM123132	20788	sterol regulatory element binding factor 2	Srebf2	3.58	3.39E-04	1.26E-02	8.15	7.27	0.88	1.84	
440	1426748_s_at	AI552141	27406	ATP-binding cassette, sub-family F (GCN20), member 3	Abcf3	-3.33	8.81E-04	2.68E-02	3.92	4.77	-0.85	-1.80	
441	1426756_at	BG064057	108148	UDP-N-acetyl-alpha-D-galactosamine:polypeptide N-acetylgalactosaminyltransferase 2	Galnt2	-3.11	1.86E-03	4.74E-02	8.13	8.85	-0.72	-1.65	
442	1426781_at	BF021045	100929	tRNA-yW synthesizing protein 1 homolog (S. cerevisiae)	Tyw1	-3.37	7.39E-04	2.33E-02	5.43	6.39	-0.96	-1.95	
443	1426784_at	AV077293	217333	tripartite motif protein 47	Trim47	-3.92	8.73E-05	4.28E-03	6.36	7.40	-1.05	-2.06	
444	1426785_s_at	BI411560	23945	monoglyceride lipase	Mgll	6.41	1.49E-10	3.75E-08	6.35	4.58	1.76	3.40	
445	1426806_at	AV313559	109019	oligonucleotide/oligosaccharide-binding fold containing 2A	Obfc2a	3.78	1.54E-04	6.70E-03	9.42	8.58	0.85	1.80	RIKEN cDNA 5830411E10 gene (oligonucleotide/oligosaccharide-binding fold containing 2A)
446	1426808_at	X16834	16854	lectin, galactose binding, soluble 3	Lgals3	5.38	7.36E-08	9.55E-06	15.60	15.20	0.34	1.27	
447	1426812_a_at	BF166604	227737	RIKEN cDNA 9130404D14 gene	9130404D14Rik	5.32	1.04E-07	1.30E-05	12.20	10.90	1.21	2.31	RIKEN cDNA 9130404D14 gene (E3 ubiquitin-protein ligase LRSAM1 (EC 6.3.2.-) (Leucine-rich repeat and sterile alpha motif-containing protein 1))
448	1426830_a_at	BB831090	229709	S-adenosylhomocysteine hydrolase-like 1	Ahcyl1	3.46	5.37E-04	1.82E-02	11.00	10.20	0.78	1.72	
449	1426945_at	AW536621	70572	RAN binding protein 5	Ranbp5	4.47	7.84E-06	5.62E-04	10.50	9.55	1.00	2.00	
450	1426946_at	AW536621	70572	RAN binding protein 5	Ranbp5	4.55	5.45E-06	4.12E-04	9.98	8.97	1.01	2.01	
451	1426965_at	BC025198	76108	RAS related protein 2a	Rap2a	3.26	1.12E-03	3.21E-02	10.80	10.00	0.73	1.66	
452	1427002_s_at	AV059397	74008	arylsulfatase G	Arsg	-3.69	2.23E-04	9.10E-03	3.63	4.50	-0.88	-1.84	RIKEN cDNA 6330406P08 gene (WD repeat domain phosphoinositide-interacting protein 1 (WIPI-1))
453	1427005_at	BM234765	20620	polo-like kinase 2 (Drosophila)	Plk2	3.21	1.35E-03	3.70E-02	11.50	10.70	0.74	1.67	
454	1427015_at	BI732921	380969	Riken cDNA C230021P08 gene	C230021P08Rik	4.82	1.47E-06	1.32E-04	4.64	3.53	1.11	2.16	1427015_at (Uncharacterized protein KIAA1602 homolog)
455	1427021_s_at	AK002778	14319	ferritin heavy chain 1	Fth1	4.08	4.60E-05	2.49E-03	15.50	15.30	0.25	1.19	
456	1427049_s_at	AW555326	319757	smoothened homolog (Drosophila)	Smo	-7.01	2.43E-12	8.65E-10	3.29	4.95	-1.66	-3.16	
457	1427193_at	BM219644	78656	bromodomain containing 8	Brd8	3.20	1.37E-03	3.75E-02	2.01	1.91	0.10	1.08	
458	1427229_at	BB123978	15357	3-hydroxy-3-methylglutaryl-Coenzyme A reductase	Hmgcr	4.09	4.26E-05	2.34E-03	8.60	7.64	0.96	1.95	
459	1427261_at	BQ176786	211652	WW, C2 and coiled-coil domain containing 1	Wwc1	4.70	2.59E-06	2.18E-04	5.36	4.12	1.24	2.37	cDNA sequence BC037006 (Protein WWC1 (WW domain-containing protein 1) (Kidney and brain protein) (KIBRA))
460	1427327_at	BB775785	231805	paired immunoglobin-like type 2 receptor alpha	Pilra	-4.69	2.76E-06	2.31E-04	5.92	7.20	-1.28	-2.43	
461	1427329_a_at	AI326478	16019	immunoglobulin heavy chain 6 (heavy chain of IgM)	Igh-6	-3.87	1.08E-04	5.07E-03	7.12	8.08	-0.96	-1.95	
462	1427345_a_at	AK002700	20887	sulfotransferase family 1A, phenol-preferring, member 1	Sult1a1	-5.57	2.61E-08	3.87E-06	3.21	4.44	-1.23	-2.34	
463	1427351_s_at	BB226392	16019	immunoglobulin heavy chain 6 (heavy chain of IgM)	Igh-6	-7.61	2.70E-14	1.28E-11	6.42	8.37	-1.95	-3.87	
464	1427385_s_at	BC003232	109711	actinin, alpha 1	Actn1	3.27	1.09E-03	3.14E-02	6.56	5.63	0.93	1.91	
465	1427393_at	M23109	14071	coagulation factor IX	F9	-5.34	9.38E-08	1.19E-05	2.60	3.34	-0.74	-1.67	
466	1427412_s_at	BQ177183	192786	Rap guanine nucleotide exchange factor (GEF) 6	Rapgef6	4.22	2.40E-05	1.45E-03	8.14	7.09	1.05	2.07	
467	1427424_at	BB399489	100047499 /// 207839	UDP-N-acetyl-alpha-D-galactosamine:polypeptide N-acetylgalactosaminyltransferase 6 /// similar to UDP-N-acetyl-alpha-D-galactosamine:polypeptide N-acetylgalactosaminyltransferase 6	Galnt6 /// LOC100047499	5.51	3.62E-08	5.18E-06	8.02	6.60	1.42	2.68	
468	1427515_at	BB637972	212167	RIKEN cDNA A530088I07 gene	A530088I07Rik	-3.11	1.90E-03	4.80E-02	3.35	3.99	-0.64	-1.56	RIKEN cDNA A530088I07 gene (unknown)
469	1427595_at	BE650741	107476	acetyl-Coenzyme A carboxylase alpha /// hypothetical gene supported by BC042725	Acaca /// LOC382567	-3.89	1.00E-04	4.79E-03	2.46	2.86	-0.40	-1.32	
470	1427604_a_at	AF011336	11981	ATPase, class II, type 9A	Atp9a	3.99	6.69E-05	3.45E-03	4.82	3.84	0.99	1.98	
471	1427689_a_at	AJ242777	57783	TNFAIP3 interacting protein 1	Tnip1	3.37	7.55E-04	2.37E-02	9.74	8.99	0.75	1.68	
472	1427736_a_at	AJ318863	54199	chemokine (C-C motif) receptor-like 2	Ccrl2	4.35	1.36E-05	9.03E-04	9.64	8.67	0.97	1.96	
473	1427798_x_at	BF580235	NA	NA	NA	-3.12	1.79E-03	4.59E-02	4.37	5.23	-0.86	-1.82	1427798_x_at (Unknown)
474	1427922_at	AW045976	66531	RIKEN cDNA 2310061C15 gene	2310061C15Rik	-3.12	1.81E-03	4.64E-02	2.90	3.37	-0.47	-1.39	RIKEN cDNA 2310061C15 gene (UPF0287 protein C16orf61 homolog)
475	1427938_at	BB046347	56309	c-myc binding protein	Mycbp	-5.59	2.27E-08	3.45E-06	5.62	7.15	-1.53	-2.90	
476	1427939_s_at	BB046347	56309	c-myc binding protein	Mycbp	-4.33	1.50E-05	9.72E-04	5.21	6.44	-1.23	-2.34	
477	1427958_at	BB478745	213012	abhydrolase domain containing 10	Abhd10	3.35	8.10E-04	2.50E-02	4.29	3.55	0.74	1.67	
478	1427959_at	BB478745	213012	abhydrolase domain containing 10	Abhd10	4.43	9.29E-06	6.50E-04	7.87	6.73	1.14	2.21	
479	1427963_s_at	BE979765	103142	retinol dehydrogenase 9	Rdh9	-3.45	5.56E-04	1.87E-02	4.14	5.06	-0.92	-1.90	
480	1427974_s_at	BB048682	12289	calcium channel, voltage-dependent, L type, alpha 1D subunit	Cacna1d	-3.62	2.89E-04	1.11E-02	5.07	6.11	-1.04	-2.05	
481	1427981_a_at	AY033912	246277	cysteine sulfinic acid decarboxylase	Csad	-3.27	1.08E-03	3.13E-02	6.02	6.92	-0.90	-1.87	
482	1427994_at	BM230330	100047115 /// 246746	CD300 antigen like family member F /// similar to CD300 antigen like family member F	Cd300lf /// LOC100047115	5.65	1.64E-08	2.56E-06	10.90	9.66	1.27	2.41	
483	1428074_at	BE981853	72309	transmembrane protein 158	Tmem158	8.07	8.88E-16	5.48E-13	8.97	7.00	1.96	3.90	RIKEN cDNA 2310037P21 gene (Transmembrane protein 158 precursor (Ras-induced senescence protein 1))
484	1428083_at	AK018202	66961	RIKEN cDNA 2310043N10 gene	2310043N10Rik	3.52	4.28E-04	1.51E-02	9.50	8.71	0.78	1.72	RIKEN cDNA 2310043N10 gene (unknown)
485	1428138_s_at	AV233429	67166	ADP-ribosylation factor-like 8B	Arl8b	3.67	2.43E-04	9.78E-03	12.20	11.30	0.84	1.79	
486	1428142_at	AK003461	104156	ets variant gene 5	Etv5	-4.89	1.00E-06	9.39E-05	9.84	11.00	-1.11	-2.16	
487	1428168_at	AK003513	68481	myelin protein zero-like 1	Mpzl1	4.52	6.27E-06	4.65E-04	6.48	5.19	1.29	2.44	
488	1428192_at	AK003597	211255	kelch repeat and BTB (POZ) domain containing 7	Kbtbd7	-3.13	1.75E-03	4.55E-02	6.80	7.61	-0.81	-1.75	
489	1428267_at	AK010512	67487	DEAH (Asp-Glu-Ala-His) box polypeptide 40	Dhx40	-3.59	3.32E-04	1.24E-02	7.12	8.02	-0.89	-1.86	
490	1428283_at	AK004699	74134	cytochrome P450, family 2, subfamily s, polypeptide 1	Cyp2s1	6.56	5.47E-11	1.45E-08	4.59	3.18	1.41	2.65	
491	1428288_at	AW488885	70273	RIKEN cDNA 2310051E17 gene	2310051E17Rik	4.28	1.84E-05	1.16E-03	8.17	7.11	1.06	2.09	
492	1428289_at	AW488885	16601 /// 70273	Kruppel-like factor 9 /// RIKEN cDNA 2310051E17 gene	2310051E17Rik /// Klf9	4.41	1.05E-05	7.20E-04	8.32	7.24	1.08	2.11	

	A	B	C	D	E	F	G	H	I	J	K	L	M
493	1428332_at	AW556858	216505	phosphoinositide-3-kinase interacting protein 1	Pik3ip1	-4.64	3.46E-06	2.81E-04	4.67	5.97	-1.31	-2.47	RIKEN cDNA 1500004A08 gene (LIM domain kinase 2 (EC 2.7.11.1) (LIMK-2))
494	1428420_a_at	AK005443	74190	RIKEN cDNA 1200009I06 gene	1200009I06Rik	6.22	5.03E-10	1.12E-07	7.81	6.16	1.65	3.14	RIKEN cDNA 1200009I06 gene (SEC6-like protein C14orf73 homolog)
495	1428431_at	BB813478	71918	RIKEN cDNA 2310047A01 gene	2310047A01Rik	-3.51	4.53E-04	1.59E-02	8.04	8.86	-0.82	-1.76	RIKEN cDNA 2310047A01 gene (unknown)
496	1428432_at	BB813478	71918	RIKEN cDNA 2310047A01 gene	2310047A01Rik	-4.22	2.43E-05	1.46E-03	6.99	8.05	-1.06	-2.08	RIKEN cDNA 2310047A01 gene (unknown)
497	1428471_at	BQ176684	20411	sorbin and SH3 domain containing 1	Sorbs1	-5.46	4.75E-08	6.50E-06	2.93	3.96	-1.03	-2.04	
498	1428484_at	AK004768	71720	oxysterol binding protein-like 3	Osbpl3	-3.17	1.54E-03	4.10E-02	5.24	6.15	-0.91	-1.88	
499	1428512_at	AK012577	70237	basic helix-loop-helix domain containing, class B9	Bhlhb9	-3.83	1.28E-04	5.83E-03	4.09	5.11	-1.02	-2.03	
500	1428527_at	AK011015	76561	sorting nexin 7	Snx7	4.73	2.20E-06	1.90E-04	9.19	8.12	1.07	2.10	
501	1428572_at	AK011545	100045716 /// 70350	brain abundant, membrane attached signal protein 1 /// similar to 22 kDa neuronal tissue-enriched acidic protein	Basp1 /// LOC100045716	3.62	2.90E-04	1.11E-02	12.70	11.90	0.82	1.76	
502	1428578_s_at	AK003571	68507	protein tyrosine phosphatase, receptor type, f polypeptide (PTPRF), interacting protein (liprin), alpha 4	Ppfia4	-3.23	1.24E-03	3.48E-02	6.38	7.25	-0.87	-1.82	
503	1428587_at	BB823331	233724	transmembrane protein 41B	Tmem41b	3.46	5.41E-04	1.83E-02	9.61	8.84	0.77	1.70	
504	1428615_at	AK008952	67168	purinergic receptor P2Y, G-protein coupled, 5	P2ry5	-4.84	1.28E-06	1.17E-04	8.78	9.87	-1.10	-2.14	
505	1428623_at	AK011193	18844	plexin A1	Plxna1	5.61	2.02E-08	3.10E-06	9.16	7.87	1.28	2.44	
506	1428667_at	AW986246	17161	monoamine oxidase A	Maoa	5.53	3.24E-08	4.71E-06	8.45	7.10	1.36	2.56	
507	1428669_at	AK012810	107771	brain expressed myelocytomatosis oncogene	Bmyc	-3.82	1.31E-04	5.95E-03	5.29	6.38	-1.09	-2.13	
508	1428673_at	AK019497	68188	symplekin	Sympk	-3.25	1.14E-03	3.24E-02	7.27	8.07	-0.80	-1.74	
509	1428700_at	AK008013	74191	purinergic receptor P2Y, G-protein coupled 13	P2ry13	-5.74	9.34E-09	1.51E-06	5.61	7.19	-1.57	-2.98	
510	1428724_at	BB283573	74737	cleavage and polyadenylation factor subunit homolog (S. cerevisiae)	Pcf11	-3.53	4.12E-04	1.47E-02	3.57	4.38	-0.82	-1.76	RIKEN cDNA 2500001H09 gene (pre-mRNA cleavage complex II protein Pcf11)
511	1428739_at	BB772205	100042420 /// 69638	RIKEN cDNA 2310040A07 gene /// similar to 2310040A07Rik protein	2310040A07Rik /// LOC100042420	-4.45	8.48E-06	6.03E-04	4.24	5.46	-1.22	-2.32	RIKEN cDNA 2310040A07 gene (unknown)
512	1428751_at	AK005771	69310	Park2 co-regulated	Pacrg	3.68	2.30E-04	9.36E-03	7.76	6.81	0.95	1.93	
513	1428777_at	AK017680	114715	sprouty protein with EVH-1 domain 1, related sequence	Spred1	-3.54	4.03E-04	1.44E-02	7.99	8.81	-0.83	-1.77	
514	1428834_at	AK012530	319520	dual specificity phosphatase 4	Dusp4	6.97	3.25E-12	1.11E-09	10.30	8.77	1.55	2.93	
515	1428861_at	AK019472	78749	RIKEN cDNA 4631422O05 gene	4631422O05Rik	-3.48	4.97E-04	1.71E-02	6.11	7.07	-0.95	-1.94	RIKEN cDNA 4631422O05 gene (Filamin A-interacting protein 1-like)
516	1428902_at	AK003880	58250	carbohydrate sulfotransferase 11	Chst11	3.98	6.97E-05	3.54E-03	6.94	5.82	1.11	2.16	RIKEN cDNA 1110020P09 gene (Carbohydrate sulfotransferase 11 (EC 2.8.2.5) (Chondroitin 4-O-sulfotransferase 1), CAST)
517	1428909_at	C85657	319269	RIKEN cDNA A130040M12 gene	A130040M12Rik	-3.22	1.29E-03	3.57E-02	8.52	9.25	-0.73	-1.66	RIKEN cDNA A130040M12 gene (unknown)
518	1428942_at	AA796766	17750	metallothionein 2	Mt2	3.89	1.01E-04	4.80E-03	8.63	7.72	0.91	1.88	
519	1428943_at	BE134119	67725	nudix (nucleoside diphosphate linked moiety X)-type motif 13	Nudt13	3.32	8.96E-04	2.72E-02	5.84	4.89	0.95	1.93	
520	1428970_at	AV113878	72117	N-acetyltransferase 13	Nat13	3.39	7.06E-04	2.24E-02	10.40	9.63	0.76	1.69	
521	1428975_at	AK003860	66329	sushi domain containing 3	Susd3	-4.05	5.14E-05	2.75E-03	7.55	8.53	-0.97	-1.96	
522	1428988_at	AK006128	76408	ATP-binding cassette, sub-family C (CFTR/MRP), member 3	Abcc3	-4.03	5.61E-05	2.97E-03	7.74	8.69	-0.96	-1.94	
523	1428989_at	AK002941	67679	RIKEN cDNA 0710001D07 gene	0710001D07Rik	-3.77	1.62E-04	6.97E-03	3.01	3.67	-0.66	-1.58	RIKEN cDNA 0710001D07 gene (unknown)
524	1429012_at	BM246754	73341	Rac/Cdc42 guanine nucleotide exchange factor (GEF) 6	Arhgef6	-3.36	7.88E-04	2.45E-02	8.58	9.35	-0.76	-1.70	
525	1429064_at	AK013596	208440	DIP2 disco-interacting protein 2 homolog C (Drosophila)	Dip2c	-3.92	8.72E-05	4.28E-03	5.92	7.00	-1.08	-2.12	RIKEN cDNA 2900024P20 gene (DIP2 disco-interacting protein 2 homolog C)
526	1429088_at	AK007400	100048380 /// 77889	limb-bud and heart /// similar to limb-bud and heart	Lbh /// LOC100048380	-5.24	1.65E-07	1.94E-05	6.90	8.21	-1.31	-2.49	
527	1429092_at	AK009497	69568	vitamin K epoxide reductase complex, subunit 1-like 1	Vkorc1l1	5.13	2.88E-07	3.16E-05	10.00	8.86	1.14	2.20	
528	1429104_at	AK012581	67803	LIM domain containing 2	Limd2	-4.33	1.50E-05	9.71E-04	8.72	9.70	-0.98	-1.97	RIKEN cDNA 0610025L06 gene (LIM domain-containing protein 2)
529	1429156_at	BF453953	66311	RIKEN cDNA 2610036L11 gene	2610036L11Rik	-3.18	1.49E-03	4.00E-02	5.57	6.48	-0.90	-1.87	RIKEN cDNA 2610036L11 gene (Cancer-up-regulated gene 2 protein)
530	1429206_at	AK014194	69288	Rho-related BTB domain containing 1	Rhobtb1	-6.92	4.63E-12	1.56E-09	5.87	7.71	-1.84	-3.58	
531	1429228_at	BE980134	75216	RIKEN cDNA 4930534B04 gene	4930534B04Rik	3.25	1.17E-03	3.32E-02	7.64	6.79	0.84	1.79	RIKEN cDNA 4930534B04 gene (Uncharacterized protein C14orf145 homolog)
532	1429233_at	BM120495	52398	septin 11	sept11	3.22	1.30E-03	3.58E-02	5.65	4.74	0.91	1.88	
533	1429237_at	BF682137	228960	syntaxin 16	Stx16	-3.53	4.08E-04	1.45E-02	7.81	8.65	-0.83	-1.78	
534	1429239_a_at	AK014587	170459	StAR-related lipid transfer (START) domain containing 4	Stard4	4.68	2.91E-06	2.43E-04	7.88	6.67	1.21	2.31	
535	1429240_at	AK014587	170459	StAR-related lipid transfer (START) domain containing 4	Stard4	4.87	1.13E-06	1.04E-04	8.31	7.11	1.20	2.30	
536	1429250_at	AV256128	110350	dynein cytoplasmic 2 heavy chain 1	Dync2h1	6.02	1.71E-09	3.38E-07	2.80	2.24	0.56	1.48	
537	1429291_at	BM225206	70247	proteasome (prosome, macropain) 26S subunit, non-ATPase, 1	Psmd1	3.81	1.39E-04	6.23E-03	11.40	10.50	0.87	1.83	
538	1429427_s_at	BB175494	21416	transcription factor 7-like 2, T-cell specific, HMG-box	Tcf7l2	-3.84	1.22E-04	5.61E-03	5.68	6.76	-1.08	-2.11	
539	1429466_s_at	AK002310	208117 /// 68318	anterior pharynx defective 1c homolog (C. elegans) /// anterior pharynx defective 1b homolog (C. elegans)	Aph1b /// Aph1c	4.51	6.51E-06	4.79E-04	11.60	10.50	1.03	2.04	
540	1429514_at	AW111876	67916	phosphatidic acid phosphatase type 2B	Ppap2b	11.31	0.00E+00	0.00E+00	9.52	6.74	2.78	6.87	
541	1429637_at	AK008987	73663	RIKEN cDNA 2210419I08 gene	2210419I08Rik	9.39	0.00E+00	0.00E+00	6.68	4.23	2.45	5.46	RIKEN cDNA 2210419I08 gene (unknown)
542	1429646_at	AK007173	74306	RIKEN cDNA 1700112C13 gene	1700112C13Rik	9.66	0.00E+00	0.00E+00	7.34	4.76	2.58	5.98	RIKEN cDNA 1700112C13 gene (Testis serine protease 6)
543	1429656_at	BB041370	69288	Rho-related BTB domain containing 1	Rhobtb1	-6.74	1.60E-11	4.79E-09	4.29	6.12	-1.83	-3.55	
544	1429682_at	AK015259	74645	RIKEN cDNA 4930431B09 gene	4930431B09Rik	5.76	8.47E-09	1.41E-06	6.14	4.55	1.59	3.02	RIKEN cDNA 4930431B09 gene (Protein FAM46C)
545	1429693_at	AK017619	13132	disabled homolog 2 (Drosophila)	Dab2	-3.39	6.91E-04	2.21E-02	5.16	6.13	-0.97	-1.96	
546	1429723_at	AK018153	66674	RIKEN cDNA 6330409N04 gene	6330409N04Rik	3.49	4.75E-04	1.65E-02	9.07	8.28	0.79	1.73	RIKEN cDNA 6330409N04 gene (Chronic lymphocytic leukemia deletion region gene 6 protein homolog)
547	1429758_at	BB046208	74211	septin 14	sept14	3.34	8.48E-04	2.61E-02	9.68	8.94	0.74	1.67	RIKEN cDNA 1700017B05 gene (Uncharacterized protein C15orf39 homolog)
548	1429772_at	BB085537	18845	plexin A2	Plxna2	4.54	5.51E-06	4.15E-04	5.09	3.93	1.16	2.23	
549	1429775_a_at	AK009736	100044979 /// 664862 /// 83924	G protein-coupled receptor 137B /// G protein-coupled receptor 137B, pseudogene /// similar to Gpr137b protein	Gpr137b /// Gpr137b-ps /// LOC100044979	5.18	2.23E-07	2.53E-05	11.80	10.60	1.18	2.27	
550	1429778_at	AK015354	71648	optineurin	Optn	4.99	6.18E-07	6.20E-05	7.33	5.96	1.36	2.57	
551	1429881_at	AK018058	76117	Rho GTPase activating protein 15	Arhgap15	-3.39	6.99E-04	2.23E-02	7.48	8.30	-0.82	-1.77	
552	1429884_at	AK005172	14270	SLIT-ROBO Rho GTPase activating protein 2	Srgap2	-4.32	1.56E-05	9.99E-04	9.13	10.10	-0.97	-1.96	
553	1429954_at	AK014135	73149	C-type lectin domain family 4, member a3	Clec4a3	-4.43	9.30E-06	6.50E-04	11.50	12.60	-1.03	-2.04	
554	1430123_a_at	AK009462	58810	aldo-keto reductase family 1, member A4 (aldehyde reductase)	Akr1a4	4.27	1.97E-05	1.22E-03	15.70	15.50	0.17	1.13	
555	1430127_a_at	AK007904	12444	cyclin D2	Ccnd2	-5.32	1.02E-07	1.28E-05	7.11	8.42	-1.31	-2.49	
556	1430259_at	AK020374	21934	tumor necrosis factor receptor superfamily, member 11a	Tnfrsf11a	-3.23	1.24E-03	3.47E-02	8.57	9.30	-0.73	-1.66	
557	1430332_a_at	AK002832	110006	glucuronidase, beta	Gusb	4.41	1.02E-05	7.06E-04	11.90	10.90	1.01	2.01	
558	1430365_at	AK005913	100978	nuclear transcription factor, X-box binding-like 1	Nfxl1	-3.81	1.38E-04	6.21E-03	5.41	6.49	-1.08	-2.12	
559	1430530_s_at	AK012340	67824	NmrA-like family domain containing 1	Nmral1	-3.24	1.19E-03	3.36E-02	7.28	8.08	-0.80	-1.74	RIKEN cDNA 1110025F24 gene (NmrA-like family domain-containing protein 1)
560	1430533_a_at	BI134907	12387	catenin (cadherin associated protein), beta 1	Ctnnb1	4.63	3.59E-06	2.90E-04	10.70	9.65	1.04	2.05	
561	1430534_at	AW825994	78416	ribonuclease, RNase A family, 6	Rnase6	-4.80	1.57E-06	1.40E-04	5.97	7.28	-1.30	-2.47	
562	1430557_at	BB009682	73979	RIKEN cDNA 4930434J08 gene	4930434J08Rik	-5.45	4.96E-08	6.73E-06	6.41	7.83	-1.42	-2.68	RIKEN cDNA 4930434J08 gene (unknown)
563	1430604_a_at	AW986632	13132	disabled homolog 2 (Drosophila)	Dab2	-4.22	2.39E-05	1.45E-03	9.67	10.60	-0.95	-1.93	
564	1430623_s_at	AV263881	109019	oligonucleotide/oligosaccharide-binding fold containing 2A	Obfc2a	3.28	1.05E-03	3.07E-02	8.04	7.23	0.81	1.75	RIKEN cDNA 5830411E10 gene (oligonucleotide/oligosaccharide-binding fold containing 2A)
565	1430726_at	AV331288	71323	Ras association (RalGDS/AF-6) domain family 8	Rassf8	4.33	1.47E-05	9.57E-04	5.71	4.51	1.20	2.30	
566	1430735_at	AK015195	67578	protein associated with topoisomerase II homolog 2 (yeast)	Patl2	4.77	1.80E-06	1.58E-04	6.34	4.99	1.35	2.56	RIKEN cDNA 4930424G05 gene (protein associated with topoisomerase II homolog 2)
567	1430829_s_at	AK016860	26383	fat mass and obesity associated	Fto	4.14	3.44E-05	1.94E-03	5.08	4.01	1.07	2.10	
568	1430878_at	AV078837	70133	RIKEN cDNA 2210406H18 gene	2210406H18Rik	4.02	5.87E-05	3.08E-03	7.61	6.56	1.06	2.08	RIKEN cDNA 2210406H18 gene (Immunoglobulin superfamily, member 6)

	A	B	C	D	E	F	G	H	I	J	K	L	M
569	1431028_a_at	AK017345	75735	pantothenate kinase 1	Pank1	-3.33	8.65E-04	2.65E-02	4.86	5.81	-0.95	-1.93	
570	1431038_at	BF137610	213391	Ras association (RalGDS/AF-6) domain family 4	Rassf4	-3.16	1.60E-03	4.22E-02	4.06	4.89	-0.83	-1.78	
571	1431050_at	BE291900	73086	ribosomal protein S6 kinase, polypeptide 5	Rps6ka5	-3.80	1.43E-04	6.33E-03	5.13	6.21	-1.08	-2.12	
572	1431056_a_at	AK017272	16956 /// 669888	lipoprotein lipase /// similar to Lipoprotein lipase precursor (LPL)	LOC669888 /// Lpl	-3.25	1.14E-03	3.24E-02	13.50	14.00	-0.52	-1.44	
573	1431130_at	AK008392	70261	RIKEN cDNA 2010110P09 gene	2010110P09Rik	-3.98	6.79E-05	3.48E-03	3.05	3.78	-0.73	-1.65	RIKEN cDNA 2010110P09 gene (Calcineurin B homologous protein 2 (Hepatocellular carcinoma- associated antigen 520 homolog))
574	1431164_at	AK017818	52187	Ras-related GTP binding D	Rragd	4.96	7.02E-07	6.92E-05	4.97	3.75	1.22	2.33	
575	1431253_s_at	BF166521	71310	TBC1 domain family, member 9	Tbc1d9	3.25	1.14E-03	3.25E-02	7.06	6.17	0.90	1.86	RIKEN cDNA 4933431N12 gene (TBC1 domain family member 9)
576	1431392_at	AK019990	15400 /// 77580	homeo box A3 /// RIKEN cDNA 5730596B20 gene	5730596B20Rik /// Hoxa3	4.60	4.32E-06	3.38E-04	3.34	2.69	0.65	1.57	RIKEN cDNA 5730596B20 gene (unknown)
577	1431424_at	AK021336	77994	RIKEN cDNA 2810055G20 gene	2810055G20Rik	-4.40	1.06E-05	7.27E-04	3.88	5.02	-1.14	-2.20	RIKEN cDNA 2810055G20 gene (unknown)
578	1431435_at	AK018213	NA	NA	NA	-3.76	1.70E-04	7.25E-03	2.24	2.53	-0.29	-1.22	1431435_at
579	1431724_a_at	AK013804	70839	purinergic receptor P2Y, G-protein coupled 12	P2ry12	-7.20	6.24E-13	2.42E-10	5.75	7.67	-1.92	-3.79	
580	1431777_a_at	AK002970	94353	high mobility group nucleosomal binding domain 3	Hmgn3	-3.23	1.22E-03	3.42E-02	7.05	7.86	-0.81	-1.76	
581	1431843_a_at	AK011965	18037	nuclear factor of kappa light polypeptide gene enhancer in B-cells inhibitor, epsilon	Nfkbie	-4.63	3.61E-06	2.90E-04	6.17	7.42	-1.24	-2.36	
582	1432230_at	AK020853	215114	huntingtin interacting protein 1	Hip1	3.24	1.18E-03	3.33E-02	3.72	3.14	0.58	1.50	
583	1432473_a_at	AK015996	67928	ATP-binding cassette, sub-family A (ABC1), member 14	Abca14	-4.43	9.62E-06	6.70E-04	3.94	5.09	-1.16	-2.23	
584	1432509_at	AK017206	76000	RIKEN cDNA 5033430I15 gene	5033430I15Rik	-3.45	5.62E-04	1.88E-02	3.67	4.50	-0.82	-1.77	RIKEN cDNA 5033430I15 gene (unknown)
585	1433443_a_at	BB705380	100040592 /// 208715	3-hydroxy-3-methylglutaryl-Coenzyme A synthase 1 /// similar to Hmgcs1 protein	Hmgcs1 /// LOC100040592	4.23	2.38E-05	1.44E-03	10.50	9.53	0.94	1.92	
586	1433444_at	BB705380	100040592 /// 208715	3-hydroxy-3-methylglutaryl-Coenzyme A synthase 1 /// similar to Hmgcs1 protein	Hmgcs1 /// LOC100040592	4.73	2.26E-06	1.93E-04	11.50	10.50	1.08	2.11	
587	1433445_x_at	BB705380	100040592 /// 208715	3-hydroxy-3-methylglutaryl-Coenzyme A synthase 1 /// similar to Hmgcs1 protein	Hmgcs1 /// LOC100040592	4.45	8.60E-06	6.10E-04	11.60	10.60	1.02	2.02	
588	1433446_at	BB705380	100040592 /// 208715	3-hydroxy-3-methylglutaryl-Coenzyme A synthase 1 /// similar to Hmgcs1 protein	Hmgcs1 /// LOC100040592	5.81	6.25E-09	1.07E-06	9.41	8.10	1.31	2.48	
589	1433460_at	BB329157	104718	tetratricopeptide repeat domain 7B	Ttc7b	3.57	3.62E-04	1.33E-02	8.84	8.03	0.82	1.76	
590	1433511_at	BI689897	83602	General transcription factor II A, 1	Gtf2a1	3.82	1.36E-04	6.15E-03	9.13	8.27	0.86	1.82	RIKEN cDNA 6330549H03 gene (Transcription initiation factor IIA subunit 1)
591	1433604_x_at	BG065457	11674	aldolase 1, A isoform	Aldoa	3.58	3.44E-04	1.28E-02	13.10	12.30	0.78	1.72	
592	1433694_at	AV270888	100047223 /// 18576	phosphodiesterase 3B, cGMP-inhibited /// similar to phosphodiesterase 3B	LOC100047223 /// Pde3b	-7.80	6.27E-15	3.32E-12	7.37	9.26	-1.89	-3.70	
593	1433695_at	BQ174762	380686	cannabinoid receptor interacting protein 1	Cnrip1	3.86	1.13E-04	5.25E-03	11.30	10.50	0.88	1.84	RIKEN cDNA 1500041B16 gene (Uncharacterized protein C2orf32 homolog)
594	1433699_at	BM241351	21929	tumor necrosis factor, alpha-induced protein 3	Tnfaip3	6.77	1.26E-11	3.79E-09	9.77	8.25	1.52	2.86	
595	1433719_at	AI854429	331004	solute carrier family 9 (sodium/hydrogen exchanger), isoform 9	Slc9a9	-3.64	2.77E-04	1.08E-02	5.63	6.66	-1.02	-2.03	
596	1433720_s_at	AI647775	103172	Nur77 downstream gene 2	Ndg2	3.13	1.73E-03	4.50E-02	8.76	8.04	0.72	1.65	
597	1433733_a_at	BG069864	12952	cryptochrome 1 (photolyase-like)	Cry1	3.75	1.76E-04	7.46E-03	8.40	7.50	0.90	1.86	
598	1433759_at	BE949451	244745	dpy-19-like 1 (C. elegans)	Dpy19l1	-3.78	1.58E-04	6.83E-03	7.32	8.24	-0.93	-1.90	
599	1433775_at	BB163333	97130	expressed sequence C77080	C77080	5.21	1.93E-07	2.24E-05	8.60	7.36	1.25	2.38	expressed sequence C77080 (Uncharacterized protein KIAA1522)
600	1433789_at	BI082172	399101	small nucleolar RNA host gene (non-protein coding) 3	Snhg3	3.41	6.57E-04	2.13E-02	4.75	3.90	0.85	1.80	
601	1433796_at	BI734389	71946	endonuclease domain containing 1	Endod1	-3.29	1.01E-03	2.98E-02	7.32	8.13	-0.81	-1.75	RIKEN cDNA 2310067E08 gene (Endonuclease domain-containing 1 protein precursor (EC 3.1.30.-))
602	1433864_at	AV254798	239393	low density lipoprotein-related protein 12	Lrp12	3.81	1.38E-04	6.19E-03	11.30	10.40	0.87	1.83	
603	1433897_at	BQ176744	103266	expressed sequence AI597468	AI597468	3.18	1.45E-03	3.93E-02	7.57	6.74	0.83	1.78	expressed sequence AI597468 (UPF0444 protein C12orf23 homolog)
604	1433933_s_at	BB553107	101488	solute carrier organic anion transporter family, member 2b1	Slco2b1	-8.59	8.91E-18	6.47E-15	7.39	9.47	-2.08	-4.22	
605	1434010_at	AV326938	72750	amyotrophic lateral sclerosis 2 (juvenile) chromosome region, candidate 13 (human)	Als2cr13	-3.27	1.07E-03	3.11E-02	8.07	8.83	-0.76	-1.69	
606	1434021_at	BG064982	230866	RIKEN cDNA C230096C10 gene	C230096C10Rik	3.48	4.98E-04	1.71E-02	10.00	9.24	0.77	1.71	RIKEN cDNA C230096C10 gene (Uncharacterized protein KIAA0090 precursor)
607	1434033_at	AI325112	21885	transducin-like enhancer of split 1, homolog of Drosophila E(spl)	Tle1	-3.80	1.43E-04	6.35E-03	6.47	7.48	-1.00	-2.01	
608	1434109_at	AV291265	212531	SH3 domain binding glutamic acid-rich protein like 2	Sh3bgrl2	3.31	9.32E-04	2.81E-02	8.99	8.24	0.75	1.68	
609	1434129_s_at	BG917242	218454	lipoma HMGIC fusion partner-like 2	Lhfpl2	5.79	7.18E-09	1.21E-06	12.80	11.60	1.29	2.44	
610	1434130_at	BG917242	218454	lipoma HMGIC fusion partner-like 2	Lhfpl2	7.50	6.48E-14	2.92E-11	11.50	9.84	1.69	3.24	
611	1434148_at	BB364520	21413	transcription factor 4	Tcf4	-3.82	1.32E-04	6.00E-03	9.48	10.30	-0.86	-1.81	
612	1434149_at	BB364520	21413	transcription factor 4	Tcf4	-3.72	2.00E-04	8.30E-03	7.67	8.56	-0.89	-1.85	
613	1434150_a_at	AV171622	393082 /// 70152	methyltransferase like 7A /// UbiE-YGHL1 fusion protein	Mettl7a /// Ubie	3.73	1.93E-04	8.07E-03	10.50	9.67	0.83	1.78	RIKEN cDNA 3300001H21 gene (methyltransferase like 7A)
614	1434151_at	AV171622	70152	methyltransferase like 7A	Mettl7a	4.23	2.36E-05	1.44E-03	10.10	9.18	0.94	1.92	RIKEN cDNA 3300001H21 gene (methyltransferase like 7A)
615	1434194_at	AV337593	17756	microtubule-associated protein 2	Mtap2	-5.04	4.73E-07	4.88E-05	2.70	3.45	-0.75	-1.68	
616	1434218_at	BB400635	215476	RIKEN cDNA C330019G07 gene	C330019G07Rik	-3.47	5.24E-04	1.78E-02	3.61	4.42	-0.81	-1.76	RIKEN cDNA C330019G07 gene (unknown)
617	1434228_at	AV255921	381511	protein phosphatase 2C, magnesium dependent, catalytic subunit	Ppm2c	4.44	9.05E-06	6.36E-04	8.92	7.90	1.02	2.03	
618	1434252_at	BB711990	319880	transmembrane and coiled coil domains 3	Tmcc3	-3.22	1.29E-03	3.57E-02	9.40	10.10	-0.72	-1.65	
619	1434253_s_at	BB711990	319880	transmembrane and coiled coil domains 3	Tmcc3	-3.16	1.57E-03	4.15E-02	8.60	9.32	-0.72	-1.65	
620	1434263_at	AV307274	73822	RIKEN cDNA F630110N24 gene	F630110N24Rik	3.44	5.81E-04	1.93E-02	12.20	11.40	0.79	1.73	RIKEN cDNA 4930404N11 gene (Fizzy-related protein homolog (Fzr) (Cdh1/Hct1 homolog))
621	1434316_at	BQ174991	100047167 /// 269941	carbohydrate (chondroitin) synthase 1 /// similar to mKIAA0990 protein	Chsy1 /// LOC100047167	-3.31	9.43E-04	2.83E-02	6.93	7.77	-0.84	-1.79	
622	1434339_at	AW548221	214459	formin binding protein 1-like	Fnbp1l	-5.26	1.44E-07	1.72E-05	9.58	10.80	-1.19	-2.28	
623	1434362_at	BM249454	98715	expressed sequence AW550831	AW550831	3.98	6.92E-05	3.53E-03	5.58	4.48	1.10	2.14	expressed sequence AW550831 (unknown)
624	1434369_a_at	AV016515	12955	crystallin, alpha B	Cryab	-4.86	1.19E-06	1.10E-04	5.24	6.60	-1.37	-2.58	
625	1434372_at	BE688358	107350	expressed sequence AW112010	AW112010	-3.93	8.62E-05	4.24E-03	11.20	12.20	-0.93	-1.90	expressed sequence AW112010 ((Small secreted protein interferon-induced), from BMDM)
626	1434378_a_at	BG868949	17122	Max dimerization protein 4	Mxd4	-4.01	6.07E-05	3.17E-03	7.58	8.54	-0.96	-1.95	
627	1434379_at	BG868949	17122	Max dimerization protein 4	Mxd4	-3.48	5.03E-04	1.72E-02	5.29	6.28	-0.99	-1.99	
628	1434399_at	AV231866	100047499 /// 207839	UDP-N-acetyl-alpha-D-galactosamine:polypeptide N-acetylgalactosaminyltransferase 6 /// similar to UDP-N-acetyl-alpha-D-galactosamine:polypeptide N-acetylgalactosaminyltransferase 6	Galnt6 /// LOC100047499	4.56	5.02E-06	3.83E-04	8.60	7.52	1.08	2.12	
629	1434402_at	BQ176258	67630	sterile alpha motif domain containing 8	Samd8	5.44	5.40E-08	7.24E-06	11.10	9.89	1.23	2.34	
630	1434437_x_at	AV301324	20135	ribonucleotide reductase M2	Rrm2	-4.21	2.57E-05	1.53E-03	6.48	7.58	-1.11	-2.15	
631	1434500_at	BF585303	117160	tweety homolog 2 (Drosophila)	Ttyh2	3.27	1.06E-03	3.11E-02	8.62	7.86	0.76	1.69	
632	1434509_at	BM250883	192786	Rap guanine nucleotide exchange factor (GEF) 6	Rapgef6	3.57	3.62E-04	1.33E-02	9.85	9.06	0.79	1.73	
633	1434510_at	BF780807	23972	3'-phosphoadenosine 5'-phosphosulfate synthase 2	Papss2	3.48	4.95E-04	1.71E-02	6.38	5.39	1.00	2.00	
634	1434520_at	AU067703	235293	sterol-C5-desaturase (fungal ERG3, delta-5-desaturase) homolog (S. cerevisae)	Sc5d	3.79	1.49E-04	6.54E-03	4.81	3.87	0.94	1.92	
635	1434548_at	BM244064	26943	serine incorporator 3	Serinc3	-3.78	1.54E-04	6.71E-03	9.66	10.50	-0.85	-1.80	
636	1434559_at	AW536415	20908	syntaxin 3	Stx3	3.22	1.27E-03	3.53E-02	9.34	8.62	0.72	1.65	
637	1434572_at	BB750646	79221	histone deacetylase 9	Hdac9	-5.91	3.48E-09	6.35E-07	4.34	5.96	-1.62	-3.07	
638	1434580_at	AV280361	224794	ectonucleotide pyrophosphatase/phosphodiesterase 4	Enpp4	-3.82	1.33E-04	6.01E-03	7.39	8.32	-0.93	-1.91	
639	1434654_at	BE570422	338337	component of oligomeric golgi complex 3	Cog3	3.40	6.79E-04	2.18E-02	9.30	8.54	0.76	1.69	
640	1434674_at	BB463428	17101	lysosomal trafficking regulator	Lyst	4.44	8.98E-06	6.33E-04	10.00	9.04	0.99	1.98	
641	1434745_at	BQ175880	12444	cyclin D2	Ccnd2	-6.84	7.74E-12	2.46E-09	6.91	8.61	-1.71	-3.26	
642	1434754_at	AU067654	380711	GTPase activating RANGAP domain-like 4	Garnl4	-4.64	3.44E-06	2.81E-04	2.47	2.99	-0.52	-1.43	
643	1434798_at	BB769890	242341	ATPase, H+ transporting, lysosomal V0 subunit D2	Atp6v0d2	5.09	3.57E-07	3.79E-05	11.80	10.60	1.16	2.23	
644	1434799_x_at	BG793658	11674	aldolase 1, A isoform	Aldoa	3.45	5.57E-04	1.87E-02	13.30	12.50	0.74	1.67	
645	1434855_at	AV298377	245847	amidohydrolase domain containing 2	Amdhd2	3.36	7.90E-04	2.46E-02	9.86	9.12	0.74	1.68	RIKEN cDNA 5730457F11 gene (Putative N-acetylglucosamine-6-phosphate deacetylase (EC 3.5.1.25) (GlcNAc 6-P deacetylase))
646	1434856_at	AV256780	329154	ankyrin repeat domain 44	Ankrd44	-3.11	1.87E-03	4.74E-02	9.39	10.10	-0.70	-1.62	RIKEN cDNA E130014H08 gene (ankyrin repeat domain 44)
647	1434872_x_at	AV170241	100040532 /// 100043481 /// 100047207 /// 100047378 /// 67281	ribosomal protein L37 /// similar to ribosomal protein L37	LOC100040532 /// LOC100043481 /// LOC100047207 /// LOC100047378 /// Rpl37	-5.89	3.84E-09	6.89E-07	14.90	15.40	-0.45	-1.37	

	A	B	C	D	E	F	G	H	I	J	K	L	M
648	1434875_a_at	AV018952	94353	high mobility group nucleosomal binding domain 3	Hmgn3	-4.59	4.50E-06	3.50E-04	7.67	8.76	-1.09	-2.13	
649	1434881_s_at	BM220945	239217	potassium channel tetramerisation domain containing 12	Kctd12	-3.26	1.10E-03	3.16E-02	10.10	10.90	-0.74	-1.67	
650	1434903_s_at	BG073776	107527	interleukin 1 receptor-like 2	Il1rl2	3.36	7.70E-04	2.41E-02	5.50	4.56	0.94	1.92	
651	1434909_at	BF462770	52187	Ras-related GTP binding D	Rragd	5.32	1.06E-07	1.31E-05	8.81	7.56	1.25	2.38	
652	1434911_s_at	BG072763	71085	Rho GTPase activating protein 19	Arhgap19	-3.16	1.56E-03	4.13E-02	7.12	7.91	-0.79	-1.73	
653	1434916_at	AV377318	69568	vitamin K epoxide reductase complex, subunit 1-like 1	Vkorc1l1	3.21	1.34E-03	3.68E-02	8.56	7.81	0.75	1.68	
654	1434929_at	BI076809	232406	cDNA sequence BC035044	BC035044	-3.10	1.92E-03	4.84E-02	5.46	6.35	-0.89	-1.85	cDNA sequence BC035044 (Unknown)
655	1434945_at	AW743924	270084	lysophosphatidylcholine acyltransferase 2	Lpcat2	-6.87	6.43E-12	2.07E-09	6.25	8.04	-1.79	-3.45	1434945_at (Acyltransferase-like 1-A (EC 2.3.1.-), Lpcat2, (lysophosphatidylcholine acyltransferase 2)
656	1434955_at	BB134696	72925	membrane-associated ring finger (C3HC4) 1	March1	-3.31	9.36E-04	2.82E-02	9.04	9.78	-0.74	-1.67	
657	1434967_at	BM938007	67263	zinc finger, SWIM domain containing 6	Zswim6	-3.94	8.20E-05	4.06E-03	8.62	9.51	-0.89	-1.86	
658	1434980_at	AV230647	320207	phosphoinositide-3-kinase, regulatory subunit 5, p101	Pik3r5	3.63	2.84E-04	1.10E-02	10.40	9.59	0.81	1.75	
659	1435050_at	BB353607	215821	DNA segment, Chr 10, Brigham & Women's Genetics 1379 expressed	D10Bwg1379e	8.05	8.88E-16	5.48E-13	7.97	5.82	2.15	4.43	
660	1435051_at	AV375936	72404	WD repeat domain 44	Wdr44	-3.14	1.67E-03	4.38E-02	6.59	7.42	-0.83	-1.78	
661	1435069_at	AW493518	408064	cDNA sequence BC064078	BC064078	-4.14	3.42E-05	1.94E-03	6.95	7.99	-1.04	-2.06	cDNA sequence BC064078 (Unknown)
662	1435119_at	BE956710	NA	Adult male medulla oblongata cDNA, RIKEN full-length enriched library, clone:6330578N16 product:unclassifiable, full insert sequence	NA	3.48	5.03E-04	1.72E-02	6.16	5.16	1.00	2.00	1435119_at
663	1435136_at	BQ177743	107823	Wolf-Hirschhorn syndrome candidate 1 (human)	Whsc1	-3.40	6.69E-04	2.16E-02	4.18	5.10	-0.92	-1.89	
664	1435155_at	BI455486	70737	cingulin	Cgn	3.64	2.71E-04	1.06E-02	4.45	3.62	0.84	1.78	
665	1435176_a_at	BF019883	15902	inhibitor of DNA binding 2	Id2	6.39	1.67E-10	4.12E-08	14.20	13.00	1.18	2.27	
666	1435198_at	AA175473	545228	hypothetical protein LOC545228	LOC545228	-3.74	1.83E-04	7.71E-03	4.20	5.21	-1.01	-2.02	RIKEN cDNA 9630025F12 gene (unknown)
667	1435251_at	AV377013	217463	sorting nexin 13	Snx13	-3.83	1.30E-04	5.92E-03	8.46	9.33	-0.87	-1.83	
668	1435306_a_at	BM234447	16551	kinesin family member 11	Kif11	-3.89	9.90E-05	4.73E-03	4.30	5.37	-1.07	-2.10	
669	1435331_at	BM241008	100048304 /// 236312	pyrin and HIN domain family, member 1 /// hypothetical protein LOC100048304	LOC100048304 /// Pyhin1	-3.27	1.06E-03	3.10E-02	10.90	11.70	-0.77	-1.71	expressed sequence AI447904 (unknown)
670	1435344_at	BM246377	100044848 /// 319491	RIKEN cDNA 1110029I05 gene /// hypothetical protein LOC100044848	1110029I05Rik /// LOC100044848	-3.49	4.74E-04	1.65E-02	3.73	4.58	-0.85	-1.81	
671	1435390_at	BG067990	71151	exonuclease domain containing 1	Exod1	-3.68	2.34E-04	9.47E-03	3.10	3.78	-0.68	-1.60	
672	1435415_x_at	BB491008	17357	MARCKS-like 1	Marcksl1	-3.41	6.47E-04	2.11E-02	6.76	7.64	-0.88	-1.84	
673	1435436_at	BI647951	NA	Transcribed locus	NA	3.57	3.61E-04	1.33E-02	7.61	6.68	0.93	1.91	1435436_at (Endothelial PAS domain-containing protein 1 (EPAS-1) (Hypoxia-inducible factor 2 alpha)
674	1435458_at	AI323550	18712	proviral integration site 1	Pim1	3.56	3.68E-04	1.34E-02	9.23	8.43	0.80	1.74	1435458_at (Proto-oncogene serine/threonine-protein kinase Pim-1 (EC 2.7.11.1))
675	1435462_at	BQ176176	433022	phosphatidylinositol-specific phospholipase C, X domain containing 2	Plcxd2	9.33	0.00E+00	0.00E+00	6.75	4.31	2.45	5.46	1435462_at (New sequence: Phospholipase C?)
676	1435463_s_at	C76327	338367	myosin ID	Myo1d	5.88	4.07E-09	7.22E-07	6.47	4.83	1.64	3.13	
677	1435485_at	BE951647	230866	RIKEN cDNA C230096C10 gene	C230096C10Rik	3.13	1.76E-03	4.55E-02	8.68	7.96	0.72	1.65	RIKEN cDNA C230096C10 gene (Uncharacterized protein KIAA0090 precursor)
678	1435490_at	BB334625	212032	hexokinase 3	Hk3	4.57	4.86E-06	3.72E-04	10.50	9.46	1.02	2.03	
679	1435492_at	BG075016	54607	suppressor of cytokine signaling 6	Socs6	3.23	1.22E-03	3.42E-02	10.10	9.33	0.72	1.64	
680	1435560_at	BI554446	16408	integrin alpha L	Itgal	-3.74	1.83E-04	7.72E-03	6.37	7.37	-1.00	-2.00	
681	1435605_at	BB125424	242894	ARP3 actin-related protein 3 homolog B (yeast)	Actr3b	-4.53	5.81E-06	4.36E-04	4.38	5.63	-1.25	-2.39	
682	1435627_x_at	AV215438	17357 /// 668321	MARCKS-like 1 /// similar to MARCKS-like protein	LOC668321 /// Marcksl1	-3.63	2.82E-04	1.09E-02	7.06	7.97	-0.91	-1.88	
683	1435636_at	AI415636	108745	RIKEN cDNA 2310051F07 gene	2310051F07Rik	4.37	1.23E-05	8.26E-04	9.53	8.55	0.98	1.97	
684	1435641_at	BB109391	320137	RIKEN cDNA 9530018I07 gene	9530018I07Rik	-3.63	2.87E-04	1.11E-02	6.48	7.44	-0.96	-1.94	
685	1435645_at	AA472735	67468 /// 676546	monocyte to macrophage differentiation-associated /// similar to monocyte to macrophage differentiation-associated	LOC676546 /// Mmd	-3.41	6.41E-04	2.09E-02	5.37	6.35	-0.97	-1.96	
686	1435679_at	BB770843	71648	optineurin	Optn	6.08	1.21E-09	2.48E-07	7.63	5.99	1.64	3.11	
687	1435689_at	AV078506	381062	RIKEN cDNA 9030025P20 gene	9030025P20Rik	-3.80	1.43E-04	6.33E-03	7.98	8.86	-0.89	-1.85	1435689_at (RIKEN cDNA 9030025P20 gene (9030025P20Rik), transcript variant 1, mRNA)
688	1435690_at	BB476775	66356	RIKEN cDNA 2310008H09 gene	2310008H09Rik	4.63	3.59E-06	2.90E-04	7.39	6.13	1.26	2.39	RIKEN cDNA 2310008H09 gene (unknown)
689	1435697_a_at	BB503614	227929	pleckstrin homology, Sec7 and coiled-coil domains, binding protein	Pscdbp	-7.52	5.68E-14	2.58E-11	7.47	9.28	-1.81	-3.50	
690	1435732_x_at	AV172216	100039636 /// 100046757 /// 11984	ATPase, H+ transporting, lysosomal V0 subunit C /// similar to vacuolar H(+)-ATPase	Atp6v0c /// LOC100039636 /// LOC100046757	8.04	8.88E-16	5.48E-13	15.80	15.50	0.34	1.27	
691	1435793_at	BM219801	208117	anterior pharynx defective 1b homolog (C. elegans)	Aph1b	3.76	1.72E-04	7.33E-03	8.03	7.09	0.94	1.92	
692	1435828_at	BM240693	17132	avian musculoaponeurotic fibrosarcoma (v-maf) AS42 oncogene homolog	Maf	-5.25	1.51E-07	1.80E-05	8.37	9.57	-1.20	-2.30	1435828_at (Transcription factor Maf (Proto-oncogene c-maf))
693	1435832_at	BB332932	192198	leucine rich repeat containing 4	Lrrc4	-5.30	1.18E-07	1.44E-05	4.16	5.59	-1.43	-2.70	
694	1435840_x_at	AI452166	625360	similar to 2-cell-stage, variable group, member 3	LOC625360	-6.36	2.06E-10	4.82E-08	7.71	9.22	-1.51	-2.84	1435840_x_at (similar to 2-cell-stage, variable group, member 3 (LOC625360), mRNA)
695	1435857_s_at	AI848048	11803	amyloid beta (A4) precursor-like protein 1	Aplp1	5.49	4.13E-08	5.80E-06	2.96	2.37	0.59	1.50	
696	1435861_at	BM222792	233977	protein tyrosine phosphatase, receptor type, f polypeptide (PTPRF), interacting protein, alpha 1	Ppfia1	3.25	1.17E-03	3.32E-02	6.29	5.35	0.93	1.91	
697	1435872_at	BE631223	NA	Transcribed locus	NA	3.37	7.55E-04	2.37E-02	6.61	5.65	0.96	1.94	1435872_at (Proto-oncogene serine/threonine-protein kinase Pim-1 (EC 2.7.11.1))
698	1435884_at	BM248471	16443	intersectin 1 (SH3 domain protein 1A)	Itsn1	-3.12	1.83E-03	4.67E-02	8.02	8.75	-0.73	-1.66	
699	1435959_at	BM246535	76117	Rho GTPase activating protein 15	Arhgap15	-3.72	1.98E-04	8.25E-03	9.00	9.84	-0.84	-1.79	
700	1435975_at	BQ175496	102442	DENN/MADD domain containing 4A	Dennd4a	-3.24	1.21E-03	3.39E-02	6.14	7.03	-0.89	-1.85	expressed sequence AI115600
701	1435981_at	BM118398	78286	neuron navigator 2	Nav2	3.34	8.52E-04	2.62E-02	7.41	6.52	0.89	1.85	1435981_att (Unknown)
702	1436034_at	AW550283	216543	centrosomal protein 68	Cep68	-5.48	4.30E-08	5.99E-06	6.02	7.49	-1.47	-2.77	
703	1436037_at	BB205589	16401	integrin alpha 4	Itga4	-4.27	1.91E-05	1.20E-03	7.79	8.80	-1.01	-2.01	1436037_at (Integrin alpha-4 precursor (Integrin alpha-IV) (VLA-4), CD49d, Itga4)
704	1436150_at	BB391675	NA	RIKEN cDNA 2310028H24 gene	NA	3.66	2.49E-04	9.94E-03	6.70	5.66	1.04	2.05	RIKEN cDNA 2310028H24 gene (Uncharacterized protein C9orf25 homolog)
705	1436187_at	BB513634	68841	RIKEN cDNA 1110054M08 gene	1110054M08Rik	-3.97	7.17E-05	3.62E-03	3.51	4.43	-0.92	-1.89	RIKEN cDNA 1110054M08 gene (unknown)
706	1436312_at	AV317621	22778	IKAROS family zinc finger 1	Ikzf1	-3.91	9.10E-05	4.42E-03	8.86	9.74	-0.88	-1.84	
707	1436336_at	AV075452	329384	peptidyl-tRNA hydrolase 1 homolog (S. cerevisiae)	Ptrh1	4.38	1.20E-05	8.11E-04	8.15	7.06	1.09	2.13	RIKEN cDNA 2210013M04 gene (Uncharacterized protein C9orf117 homolog)
708	1436337_at	BF453911	652925	RIKEN cDNA 4930420K17 gene	4930420K17Rik	-4.52	6.07E-06	4.51E-04	8.22	9.26	-1.04	-2.06	RIKEN cDNA E030031F02 gene (cyclin D binding myb-like transcription factor 1 isoform 2)
709	1436346_at	AV246882	235505	CD109 antigen	Cd109	5.29	1.20E-07	1.46E-05	8.64	7.37	1.27	2.41	
710	1436405_at	BG068753	238130	dedicator of cytokinesis 4	Dock4	-3.61	3.03E-04	1.15E-02	6.21	7.19	-0.98	-1.97	
711	1436448_a_at	AA833146	19224	prostaglandin-endoperoxide synthase 1	Ptgs1	-5.02	5.10E-07	5.25E-05	5.79	7.16	-1.38	-2.60	
712	1436512_at	BI964400	320982 /// 632433	ADP-ribosylation factor-like 4C /// similar to ADP-ribosylation factor-like protein 7	Arl4c /// LOC632433	-3.18	1.45E-03	3.93E-02	8.97	9.69	-0.72	-1.64	
713	1436520_at	BB378317	382643	AHNAK nucleoprotein 2	Ahnak2	4.31	1.65E-05	1.05E-03	9.67	8.71	0.96	1.94	expressed sequence AI450948 (AHNAK nucleoprotein 2)
714	1436541_at	BB452927	66356	RIKEN cDNA 2310008H09 gene	2310008H09Rik	5.01	5.58E-07	5.65E-05	7.51	6.17	1.35	2.55	RIKEN cDNA 2310008H09 gene (unknown)
715	1436555_at	AV244175	11988	solute carrier family 7 (cationic amino acid transporter, y+ system), member 2	Slc7a2	7.72	1.20E-14	6.07E-12	8.00	5.95	2.05	4.14	expressed sequence AI158848 (Slc7a2)
716	1436576_at	BB239429	215900	RIKEN cDNA A630077B13 gene	A630077B13Rik	-6.29	3.21E-10	7.39E-08	8.18	9.63	-1.45	-2.74	RIKEN cDNA A630077B13 gene (Protein FAM26F)
717	1436580_at	BB193498	52120	Heparan-alpha-glucosaminide N-acetyltransferase	Hgsnat	-4.05	5.17E-05	2.75E-03	3.59	4.55	-0.96	-1.95	
718	1436618_at	BB379739	94282	sideroflexin 5	Sfxn5	-4.25	2.12E-05	1.31E-03	4.11	5.25	-1.14	-2.21	
719	1436633_at	BB323723	380741	Transcribed locus, strongly similar to XP_999396.1 hypothetical protein [Mus musculus]	NA	-5.48	4.15E-08	5.81E-06	5.64	7.15	-1.51	-2.84	
720	1436660_at	BB667837	81910	ribosome binding protein 1	Rrbp1	3.65	2.65E-04	1.05E-02	4.50	3.65	0.85	1.80	
721	1436763_a_at	AI267126	16601 /// 70273	Kruppel-like factor 9 /// RIKEN cDNA 2310051E17 gene	2310051E17Rik /// Klf9	3.26	1.13E-03	3.22E-02	5.95	5.01	0.93	1.91	
722	1436808_x_at	AI324988	17218	minichromosome maintenance deficient 5, cell division cycle 46 (S. cerevisiae)	Mcm5	-4.42	9.95E-06	6.90E-04	8.21	9.23	-1.02	-2.03	

	A	B	C	D	E	F	G	H	I	J	K	L	M
723	1436822_x_at	AW558862	100040745 /// 100040929 /// 100042067 /// 100042583 ///	hexokinase 1 /// ribosomal protein L17 /// predicted gene, EG620155 /// similar to 60S ribosomal protein L17 (L23) (Amino acid starvation-induced protein) (ASI) /// similar to Rpl17	EG620155 /// Hk1 /// LOC100040745 /// LOC100040929 /// LOC100042067 ///	-3.31	9.19E-04	2.78E-02	15.00	15.30	-0.25	-1.19	
724	1436890_at	BG963358	227620	UDP-N-acteylglucosamine pyrophosphorylase 1-like 1	Uap1l1	3.23	1.23E-03	3.45E-02	11.40	10.70	0.74	1.67	
725	1436905_x_at	BB218107	16792	lysosomal-associated protein transmembrane 5	Laptm5	-3.59	3.25E-04	1.22E-02	14.70	15.00	-0.34	-1.26	
726	1436994_a_at	BB533903	50708	histone cluster 1, H1c	Hist1h1c	-3.45	5.56E-04	1.87E-02	10.60	11.40	-0.80	-1.75	
727	1436995_a_at	AV124660	19941	ribosomal protein L26	Rpl26	-3.14	1.69E-03	4.42E-02	14.10	14.50	-0.40	-1.32	
728	1436999_at	AI504908	98496	RIKEN cDNA 5033414K04 gene	5033414K04Rik	-3.20	1.37E-03	3.75E-02	12.10	12.80	-0.72	-1.64	expressed sequence AL024069 (PTB-containing, cubilin and LRP1-interacting protein (P-CLI1) (Phosphotyrosine interaction domain-containing protein 1))
729	1437012_x_at	BB226235	223864	Rap guanine nucleotide exchange factor (GEF) 3	Rapgef3	4.94	7.91E-07	7.67E-05	6.15	4.76	1.39	2.62	
730	1437046_x_at	AI639566	75007	RIKEN cDNA 4930504E06 gene	4930504E06Rik	3.31	9.40E-04	2.82E-02	10.50	9.74	0.74	1.67	RIKEN cDNA 4930504E06 gene (Annexin A9 (Annexin-9) (Annexin-31) (Annexin XXXI))
731	1437072_at	BM241218	232201	Rho GTPase activating protein 25	Arhgap25	3.65	2.61E-04	1.03E-02	10.70	9.88	0.82	1.76	
732	1437152_at	BG072837	108797	mex3 homolog B (C. elegans)	Mex3b	-7.38	1.56E-13	6.70E-11	6.83	8.68	-1.85	-3.60	
733	1437174_at	BB405795	211586 /// 319491	transcription factor Dp 2 /// RIKEN cDNA 1110029I05 gene	1110029I05Rik /// Tfdp2	-5.15	2.67E-07	2.97E-05	2.93	3.86	-0.94	-1.92	
734	1437181_at	BM121149	93834	pellino 2	Peli2	-3.19	1.40E-03	3.82E-02	6.88	7.70	-0.82	-1.76	
735	1437226_x_at	AV110584	17357	MARCKS-like 1	Marcksl1	-5.42	5.83E-08	7.74E-06	9.59	10.80	-1.23	-2.34	
736	1437244_at	BB770972	100047967 /// 237436	growth arrest-specific 2 like 3 /// similar to growth arrest-specific 2 like 3	Gas2l3 /// LOC100047967	3.11	1.88E-03	4.78E-02	10.80	10.10	0.70	1.62	
737	1437245_at	BB778966	320782	transmembrane protein 154	Tmem154	5.01	5.46E-07	5.56E-05	7.11	5.72	1.39	2.62	RIKEN cDNA 9930117H01 gene (Transmembrane protein 154 precursor)
738	1437250_at	AV298358	381269	melanoregulin	Mreg	3.81	1.40E-04	6.25E-03	7.14	6.09	1.05	2.07	
739	1437279_x_at	BB533095	20969	syndecan 1	Sdc1	9.80	0.00E+00	0.00E+00	11.10	8.88	2.21	4.63	
740	1437302_at	AV083350	11555	adrenergic receptor, beta 2	Adrb2	-6.81	9.84E-12	3.06E-09	5.75	7.57	-1.83	-3.55	
741	1437304_at	AV276986	208650	Casitas B-lineage lymphoma b	Cblb	3.84	1.23E-04	5.64E-03	7.99	7.03	0.96	1.95	
742	1437313_x_at	C85885	97165	high mobility group box 2	Hmgb2	-3.87	1.10E-04	5.16E-03	8.63	9.51	-0.88	-1.84	
743	1437356_at	BM242490	321019	Epstein-Barr virus induced gene 2	Ebi2	-4.64	3.46E-06	2.81E-04	9.74	10.80	-1.05	-2.07	
744	1437405_a_at	BB787243	16010	insulin-like growth factor binding protein 4	Rab39	-6.13	8.92E-10	1.88E-07	9.62	11.00	-1.40	-2.63	
745	1437406_x_at	BB787243	16010	insulin-like growth factor binding protein 4	Igfbp4	-5.44	5.19E-08	7.00E-06	7.45	8.76	-1.31	-2.48	
746	1437414_at	AW987152	228913	zinc finger protein 217	Zfp217	-3.56	3.65E-04	1.33E-02	7.45	8.31	-0.86	-1.82	
747	1437463_x_at	BB532080	21810	transforming growth factor, beta induced	Tgfbi	-7.99	1.33E-15	7.89E-13	9.29	11.10	-1.84	-3.57	
748	1437466_at	AV315205	11658	activated leukocyte cell adhesion molecule	Alcam	4.24	2.22E-05	1.37E-03	10.10	9.17	0.94	1.92	
749	1437467_at	AV315205	11658	activated leukocyte cell adhesion molecule	Alcam	3.99	6.67E-05	3.45E-03	10.70	9.80	0.89	1.86	
750	1437497_a_at	C77384	15519	heat shock protein 90kDa alpha (cytosolic), class A member 1	Hsp90aa1	3.62	2.99E-04	1.14E-02	11.70	10.80	0.83	1.78	
751	1437594_x_at	BB376188	100046871 /// 78928	phosphatidylinositol glycan anchor biosynthesis, class T /// similar to GPI transamidase component PIG-T precursor (Phosphatidylinositol-glycan biosynthesis class T protein) (Neuronal development-associated protein 7)	LOC100046871 /// Pigt	-5.33	1.01E-07	1.27E-05	2.31	2.82	-0.51	-1.42	
752	1437618_x_at	BB273882	64450	G protein-coupled receptor 85	Gpr85	-4.34	1.41E-05	9.25E-04	6.24	7.40	-1.16	-2.24	
753	1437621_x_at	AV216768	236539 /// 665516 /// 666036 /// 668771 /// 675316	3-phosphoglycerate dehydrogenase /// similar to 3-phosphoglycerate dehydrogenase /// predicted gene, EG668771 /// hypothetical protein LOC675316	EG668771 /// LOC665516 /// LOC666036 /// LOC675316 /// Phgdh	3.57	3.64E-04	1.33E-02	6.36	5.33	1.02	2.03	1437621_x_at (D-3-PHOSPHOGLYCERATE DEHYDROGENASE (EC 1.1.1.95) (PGDH))
754	1437635_at	AW146002	73379	discoidin, CUB and LCCL domain containing 2	Dcbld2	4.23	2.31E-05	1.41E-03	6.32	5.11	1.21	2.31	
755	1437667_a_at	AW553304	12014	BTB and CNC homology 2	Bach2	6.41	1.50E-10	3.75E-08	3.65	2.64	1.01	2.01	RIKEN cDNA E030004N02 gene (Transcription regulator protein BACH2 (BTB and CNC homolog 2)
756	1437726_x_at	BB111335	12260	complement component 1, q subcomponent, beta polypeptide	C1qb	-5.56	2.65E-08	3.92E-06	13.60	14.40	-0.80	-1.74	
757	1437762_at	BB130995	270160	RAB39, member RAS oncogene family	Rab39	-5.46	4.87E-08	6.65E-06	7.42	8.74	-1.32	-2.49	
758	1437816_at	BF456245	18508	Paired box gene 6	Pax6	4.54	5.50E-06	4.15E-04	3.57	2.85	0.73	1.66	
759	1437865_at	AW546433	219140	spermatogenesis associated 13	Spata13	-3.33	8.80E-04	2.68E-02	3.61	4.39	-0.77	-1.71	
760	1437916_at	BB076850	50794	Adult male hypothalamus cDNA, RIKEN full-length enriched library, clone:A230066G06 product:unclassifiable, full insert sequence	NA	-3.60	3.21E-04	1.20E-02	3.11	3.78	-0.66	-1.58	
761	1437939_s_at	BM237633	13032	cathepsin C	Ctsc	-9.37	7.31E-21	5.78E-18	7.17	9.47	-2.29	-4.91	
762	1437942_x_at	AV100095	71924	epsilon-tubulin 1	Tube1	6.10	1.07E-09	2.22E-07	3.98	2.87	1.11	2.16	
763	1437992_x_at	BB039269	14609	gap junction protein, alpha 1	Gja1	-3.87	1.11E-04	5.18E-03	3.25	4.04	-0.79	-1.73	
764	1438009_at	W91024	319171 /// 665433	histone cluster 1, H2ao /// similar to histone 2a	Hist1h2ao /// RP23-480B19.10	-4.48	7.44E-06	5.40E-04	10.40	11.50	-1.04	-2.06	
765	1438041_at	BG963325	18583	phosphodiesterase 7A	Pde7a	-3.34	8.30E-04	2.56E-02	4.22	5.12	-0.90	-1.87	
766	1438068_at	BB251859	NA	Transcribed locus, strongly similar to XP_579813.1 hypothetical protein XP_579813 [Rattus norvegicus]	NA	4.14	3.41E-05	1.93E-03	6.31	5.13	1.18	2.27	1438068_at (Unknown)
767	1438118_x_at	AV147875	22352	vimentin	Vim	4.64	3.53E-06	2.86E-04	15.20	14.80	0.43	1.35	
768	1438161_s_at	BB251459	106344	replication factor C (activator 1) 4	Rfc4	-3.24	1.21E-03	3.40E-02	5.59	6.51	-0.92	-1.89	
769	1438163_x_at	BB451238	215160	Rhomboid domain containing 2	Rhbdd2	3.50	4.63E-04	1.62E-02	8.82	8.02	0.80	1.75	1438163_x_at (Unknown)
770	1438165_x_at	BB559097	26949	vesicle amine transport protein 1 homolog (T californica)	Vat1	4.33	1.52E-05	9.81E-04	11.00	10.10	0.98	1.97	
771	1438169_a_at	BB009122	232288	FERM domain containing 4B	Frmd4b	-3.34	8.42E-04	2.59E-02	9.62	10.40	-0.75	-1.68	
772	1438181_x_at	BB302809	69742	TM2 domain containing 2	Tm2d2	-3.17	1.52E-03	4.06E-02	9.77	10.50	-0.71	-1.64	
773	1438201_at	AV290622	381511	protein phosphatase 2C, magnesium dependent, catalytic subunit	Ppm2c	3.64	2.69E-04	1.06E-02	9.14	8.31	0.82	1.77	
774	1438223_at	BG065705	NA	NA	NA	3.13	1.75E-03	4.54E-02	5.88	4.98	0.90	1.86	1438223_at (Unknown)
775	1438291_x_at	AV069169	100040532 /// 100043481 /// 100047207 /// 100047378 /// 67281	ribosomal protein L37 /// similar to ribosomal protein L37	LOC100040532 /// LOC100043481 /// LOC100047207 /// LOC100047378 /// Rpl37	-3.11	1.85E-03	4.71E-02	10.30	11.00	-0.71	-1.64	
776	1438306_at	AV340072	71816	ring finger protein 180	Rnf180	-3.35	8.08E-04	2.50E-02	6.90	7.76	-0.85	-1.81	
777	1438320_s_at	BB464359	17220	minichromosome maintenance deficient 7 (S. cerevisiae)	Mcm7	-3.73	1.91E-04	8.00E-03	7.32	8.23	-0.91	-1.88	
778	1438322_x_at	BB028312	14137	farnesyl diphosphate farnesyl transferase 1	Fdft1	5.56	2.71E-08	4.00E-06	8.29	6.90	1.39	2.62	
779	1438390_s_at	AV105428	30939	pituitary tumor-transforming 1	Pttg1	-5.14	2.80E-07	3.09E-05	9.28	10.40	-1.16	-2.23	
780	1438431_at	BB197269	26874	ATP-binding cassette, sub-family D (ALD), member 2	Abcd2	-3.92	9.01E-05	4.40E-03	6.00	7.07	-1.08	-2.11	
781	1438442_at	AI450236	99634	expressed sequence AI450236	AI450236	-3.27	1.08E-03	3.14E-02	5.40	6.33	-0.93	-1.91	expressed sequence AI450236 (unknown)
782	1438498_at	AI662455	574428	zinc finger, MYND-type containing 15	Zmynd15	-3.31	9.47E-04	2.84E-02	4.96	5.91	-0.95	-1.93	1438498_at (zinc finger, MYND-type containing 15)
783	1438504_x_at	BB120486	67623	Transmembrane 7 superfamily member 3	Tm7sf3	-3.11	1.84E-03	4.70E-02	7.87	8.61	-0.73	-1.66	
784	1438511_a_at	BB408123	66214	RIKEN cDNA 1190002H23 gene	1190002H23Rik	3.32	9.00E-04	2.73E-02	4.74	3.91	0.83	1.77	RIKEN cDNA 1190002H23 gene (Response gene to complement 32 protein (RGC-32))
785	1438568_at	BB373312	244238	MAS-related GPR, member E	Mrgpre	6.13	8.62E-10	1.82E-07	6.58	4.86	1.71	3.28	
786	1438577_at	BB376947	NA	Transcribed locus	NA	-3.53	4.23E-04	1.50E-02	4.02	4.95	-0.92	-1.90	1438577_at (Unknown)
787	1438629_x_at	AV166504	14824	granulin	Grn	3.20	1.39E-03	3.81E-02	14.90	14.50	0.35	1.28	
788	1438634_x_at	BB105164	16796	LIM and SH3 protein 1	Lasp1	3.09	1.98E-03	4.96E-02	13.70	13.10	0.60	1.51	
789	1438677_at	AW764208	227937	plakophilin 4	Pkp4	5.32	1.05E-07	1.30E-05	7.21	5.74	1.47	2.77	
790	1438713_at	BB391868	71323	Ras association (RalGDS/AF-6) domain family 8	NA	6.20	5.69E-10	1.25E-07	6.85	5.12	1.73	3.32	
791	1438852_x_at	BB099487	17219	minichromosome maintenance deficient 6 (MIS5 homolog, S. pombe) (S. cerevisiae)	Mcm6	-4.33	1.51E-05	9.72E-04	7.58	8.62	-1.04	-2.05	
792	1438855_x_at	BB233088	21928	tumor necrosis factor, alpha-induced protein 2	Tnfaip2	5.21	1.89E-07	2.21E-05	12.40	11.20	1.18	2.27	
793	1438882_at	BB199681	73910	Rho GTPase activating protein 18	NA	-3.26	1.10E-03	3.16E-02	3.37	4.05	-0.68	-1.61	
794	1438930_s_at	BB130002	17257	methyl CpG binding protein 2	Mecp2	-4.80	1.56E-06	1.40E-04	3.89	5.15	-1.25	-2.38	
795	1438931_s_at	AV016566	100047324 /// 140742	sestrin 1 /// similar to Sesn1 protein	LOC100047324 /// Sesn1	-3.13	1.77E-03	4.57E-02	11.20	11.90	-0.74	-1.67	
796	1438937_x_at	AI385586	11727	angiogenin, ribonuclease, RNase A family, 5	Ang	-3.80	1.47E-04	6.46E-03	4.31	5.35	-1.04	-2.06	
797	1439012_a_at	BB030204	13178	deoxycytidine kinase	Dck	-3.89	1.01E-04	4.80E-03	12.70	13.50	-0.76	-1.70	
798	1439079_a_at	BM240030	59079	Erbb2 interacting protein	Erbb2ip	-3.22	1.26E-03	3.51E-02	4.09	4.94	-0.85	-1.80	
799	1439106_at	AV320128	242466	zinc finger protein 462	Zfp462	3.18	1.45E-03	3.92E-02	4.77	3.97	0.80	1.74	
800	1439141_at	BG145550	110168	G protein-coupled receptor 18	Gpr18	-5.66	1.50E-08	2.36E-06	4.89	6.47	-1.58	-2.99	
801	1439148_a_at	BE914497	18641	phosphofructokinase, liver, B-type	Pfkl	3.38	7.37E-04	2.32E-02	9.51	8.76	0.75	1.68	
802	1439189_at	BB498793	329679	RIKEN cDNA D630023B12 gene	D630023B12Rik	3.39	7.00E-04	2.23E-02	12.60	11.90	0.77	1.70	RIKEN cDNA D630023B12 gene (MKIAA1450 protein)
803	1439205_at	BM122872	18019	nuclear factor of activated T-cells, cytoplasmic, calcineurin-dependent 2	Nfatc2	-3.81	1.40E-04	6.26E-03	7.45	8.38	-0.92	-1.89	
804	1439255_s_at	BB726971	100044979 /// 664862 /// 83924	G protein-coupled receptor 137B /// G protein-coupled receptor 137B, pseudogene /// similar to Gpr137b protein	Gpr137b /// Gpr137b-ps /// LOC100044979	5.13	2.94E-07	3.22E-05	13.70	12.60	1.05	2.07	
805	1439256_x_at	BB726971	664862	G protein-coupled receptor 137B, pseudogene	Gpr137b-ps	4.36	1.33E-05	8.83E-04	12.70	11.80	0.98	1.97	
806	1439269_x_at	BB407228	17220	minichromosome maintenance deficient 7 (S. cerevisiae)	Mcm7	-4.36	1.32E-05	8.80E-04	5.18	6.42	-1.23	-2.35	

	A	B	C	D	E	F	G	H	I	J	K	L	M
807	1439302_at	AV266743	231380	ubiquitin-like modifier activating enzyme 6	Uba6	-3.17	1.51E-03	4.03E-02	3.93	4.74	-0.81	-1.75	RIKEN cDNA 5730469D23 gene (unknown)
808	1439343_at	BB202287	15101	Transcribed locus	NA	-6.72	1.80E-11	5.30E-09	2.57	3.62	-1.05	-2.07	
809	1439375_x_at	AV030922	11674 /// 237155	aldolase 1, A isoform /// similar to Fructose-bisphosphate aldolase A (Muscle-type aldolase) (Aldolase 1)	Aldoa /// LOC237155	3.35	8.21E-04	2.53E-02	12.20	11.40	0.77	1.71	
810	1439454_x_at	AV337733	69742	TM2 domain containing 2	Tm2d2	-3.18	1.48E-03	3.98E-02	10.10	10.80	-0.72	-1.65	
811	1439497_at	BG065013	108807	RIKEN cDNA 4933415E08 gene	4933415E08Rik	-3.64	2.71E-04	1.06E-02	5.84	6.86	-1.01	-2.02	RIKEN cDNA 4933415E08 gene (unknown)
812	1439622_at	AV291679	213391	Ras association (RalGDS/AF-6) domain family 4	Rassf4	-6.03	1.61E-09	3.22E-07	7.51	8.96	-1.45	-2.73	
813	1439680_at	BB187486	22035	tumor necrosis factor (ligand) superfamily, member 10	Tnfsf10	-3.38	7.31E-04	2.31E-02	6.10	7.03	-0.93	-1.90	
814	1439755_at	BM936013	217692	signal-induced proliferation-associated 1 like 1	NA	-6.80	1.05E-11	3.25E-09	3.41	5.07	-1.65	-3.14	
815	1439787_at	BM124050	18439	purinergic receptor P2X, ligand-gated ion channel, 7	P2rx7	-3.25	1.14E-03	3.24E-02	6.59	7.45	-0.86	-1.81	
816	1439808_at	BQ176089	320495	RIKEN cDNA A130090K04 gene	A130090K04Rik	-5.82	5.93E-09	1.02E-06	2.78	3.80	-1.01	-2.01	
817	1439819_at	BG070233	101736	expressed sequence AU015263	AU015263	-5.83	5.57E-09	9.59E-07	3.74	5.24	-1.50	-2.82	
818	1439830_at	AV377656	675366	similar to mitogen activated protein kinase kinase kinase 5	LOC675366	-4.90	9.48E-07	8.97E-05	5.10	6.48	-1.38	-2.61	
819	1439831_at	AW111920	NA	Transcribed locus	NA	-3.70	2.17E-04	8.89E-03	9.15	9.98	-0.83	-1.78	1439831_at (Interferon inducible GTPase ??)
820	1439902_at	BB552085	12273	complement component 5a receptor 1	C5ar1	-3.12	1.80E-03	4.62E-02	8.97	9.67	-0.70	-1.63	1439902_at (C5a anaphylatoxin chemotactic receptor (C5a-R) (C5aR) (CD88 antigen)
821	1439906_at	BB184086	NA	0 day neonate eyeball cDNA, RIKEN full-length enriched library, clone:E130004J04 product:unclassifiable, full insert sequence	NA	-4.21	2.51E-05	1.50E-03	5.13	6.32	-1.20	-2.29	1439906_at (ETS translocation variant 1 (Protein ER81), Etv1)
822	1439946_at	BM221121	17260	myocyte enhancer factor 2C	NA	-3.10	1.93E-03	4.86E-02	4.39	5.25	-0.86	-1.81	
823	1439956_at	BE692425	69774	Membrane-spanning 4-domains, subfamily A, member 6B	Ms4a6b	-3.30	9.58E-04	2.86E-02	10.70	11.50	-0.77	-1.71	1439956_at (Membrane-spanning 4-domains subfamily A member 6B)
824	1439995_at	AV251613	97086	Na+/H+ exchanger domain containing 2	Nhedc2	4.34	1.41E-05	9.25E-04	5.46	4.28	1.17	2.26	expressed sequence C80638 (Na+/H+ exchanger domain containing 2)
825	1440105_at	AV274019	244694	jumonji domain containing 2D	Jmjd2d	-3.90	9.71E-05	4.66E-03	2.33	2.68	-0.34	-1.27	1440105_at (JmjC domain-containing histone demethylation protein 3D (EC 1.14.11.-), Jumonji domain…)
826	1440193_at	AV346451	106585	ankyrin repeat domain 12	Ankrd12	4.13	3.62E-05	2.03E-03	8.50	7.51	0.98	1.98	
827	1440209_at	AI449126	72925	membrane-associated ring finger (C3HC4) 1	March1	-3.78	1.56E-04	6.74E-03	7.84	8.73	-0.89	-1.85	
828	1440226_at	BB088782	240034	zinc finger protein 760	Zfp760	-3.44	5.77E-04	1.92E-02	3.98	4.87	-0.89	-1.86	cDNA sequence BC029103 (zinc finger protein 760)
829	1440343_at	BQ174267	73086	ribosomal protein S6 kinase, polypeptide 5	Rps6ka5	-4.67	2.99E-06	2.49E-04	3.80	5.00	-1.20	-2.29	
830	1440388_at	AV336852	NA	Transcribed locus	NA	-4.67	3.01E-06	2.50E-04	5.33	6.64	-1.31	-2.49	1440388_at (Unknown)
831	1440421_at	BB485539	78833	RIKEN cDNA 2700085M18 gene	NA	4.35	1.36E-05	8.99E-04	2.60	2.24	0.36	1.28	RIKEN cDNA 2700085M18 gene (unknown)
832	1440522_at	BB349472	331484	predicted gene, ENSMUSG00000073019	ENSMUSG00000073019	-3.67	2.47E-04	9.89E-03	5.44	6.49	-1.04	-2.06	1440522_at (Unknown)
833	1440590_at	BB237434	98910	USP6 N-terminal like	NA	5.57	2.52E-08	3.76E-06	2.54	2.12	0.41	1.33	
834	1440684_at	BB187472	270084	lysophosphatidylcholine acyltransferase 2	Lpcat2	-4.79	1.65E-06	1.46E-04	4.45	5.79	-1.33	-2.52	1440684_at (Acyltransferase-like 1-A (EC 2.3.1.-), Lpcat2, (lysophosphatidylcholine acyltransferase 2)
835	1440865_at	BB193024	213002	interferon induced transmembrane protein 6	Ifitm6	-3.45	5.68E-04	1.90E-02	10.00	10.80	-0.78	-1.72	
836	1441135_at	BB165387	100038519	Predicted gene, ENSMUSG00000053218	ENSMUSG00000053218	3.22	1.28E-03	3.54E-02	3.22	2.78	0.44	1.36	
837	1441139_at	BB321858	328186	predicted gene, ENSMUSG00000071543	ENSMUSG00000071543	-3.63	2.83E-04	1.09E-02	5.18	6.22	-1.04	-2.05	1441139_at (Unknown)
838	1441386_at	BB336546	107746	Transcribed locus	NA	3.62	2.98E-04	1.14E-02	3.08	2.63	0.45	1.36	
839	1441536_at	AV362707	208715	3-hydroxy-3-methylglutaryl-Coenzyme A synthase 1	Hmgcs1	4.33	1.46E-05	9.48E-04	4.59	3.58	1.00	2.00	
840	1441548_at	BM119551	232288	FERM domain containing 4B	Frmd4b	-3.39	7.04E-04	2.24E-02	3.41	4.14	-0.73	-1.66	
841	1441811_x_at	AU040201	66058	transmembrane protein 176A	Tmem176a	-4.12	3.86E-05	2.14E-03	6.35	7.44	-1.09	-2.14	RIKEN cDNA 0610011I04 gene (Transmembrane protein 176A (Kidney-expressed gene 2 protein) (Gene signature 188))
842	1441831_x_at	BB377664	12728	Chloride channel 5	Clcn5	-4.30	1.75E-05	1.10E-03	3.23	4.12	-0.89	-1.85	
843	1441855_x_at	BB554288	14825	chemokine (C-X-C motif) ligand 1	Cxcl1	7.24	4.37E-13	1.73E-10	7.42	5.43	1.99	3.96	
844	1441871_at	AV057045	69798	RIKEN cDNA 1810044D09 gene	1810044D09Rik	4.13	3.61E-05	2.03E-03	5.67	4.53	1.15	2.22	RIKEN cDNA 1810044D09 gene (unknown)
845	1442014_at	BB531645	15982	Transcribed locus	NA	3.47	5.13E-04	1.75E-02	4.64	3.80	0.84	1.79	
846	1442028_at	AI593864	14422	beta-1,4-N-acetyl-galactosaminyl transferase 2	B4galnt2	-3.53	4.08E-04	1.45E-02	2.76	3.24	-0.49	-1.40	expressed sequence AI593864 (Beta-1,4 N-acetylgalactosaminyltransferase 2 (EC 2.4.1.-))
847	1442137_at	BB653800	70252	RIKEN cDNA 2010000I03 gene	2010000I03Rik	-3.40	6.81E-04	2.19E-02	4.23	5.15	-0.92	-1.89	RIKEN cDNA 2010000I03 gene (unknown)
848	1442348_at	BB549862	NA	NA	NA	-3.33	8.69E-04	2.66E-02	3.51	4.26	-0.75	-1.68	1442348_at (Unknown)
849	1442432_x_at	BE630630	380753	ataxin 7-like 1	Atxn7l1	-3.26	1.11E-03	3.20E-02	3.96	4.79	-0.84	-1.79	
850	1442604_at	BB479244	319955	excision repair cross-complementing rodent repair deficiency, complementation group 6	Ercc6	3.43	6.02E-04	1.98E-02	4.34	3.57	0.77	1.70	
851	1442676_at	AV356118	17161	monoamine oxidase A	Maoa	4.39	1.13E-05	7.69E-04	5.77	4.55	1.22	2.33	
852	1442764_at	BE456272	225888	suppressor of variegation 4-20 homolog 1 (Drosophila)	Suv420h1	-4.07	4.75E-05	2.56E-03	3.20	4.02	-0.82	-1.77	
853	1443086_at	BB534113	11658	activated leukocyte cell adhesion molecule	Alcam	3.91	9.14E-05	4.44E-03	7.26	6.19	1.07	2.09	
854	1443104_at	BB541236	71310	0 day neonate eyeball cDNA, RIKEN full-length enriched library, clone:E130112O04 product:unclassifiable, full insert sequence	NA	3.27	1.09E-03	3.15E-02	4.62	3.83	0.79	1.73	RIKEN cDNA 4933431N12 gene (TBC1 domain family member 9)
855	1443163_at	BB049001	214922	solute carrier family 39 (zinc transporter), member 2	Slc39a2	9.81	0.00E+00	0.00E+00	5.46	3.22	2.24	4.73	
856	1443208_at	BB056087	83924	transmembrane 7 superfamily member 1	NA	3.67	2.40E-04	9.64E-03	7.65	6.69	0.96	1.94	
857	1443534_at	BM201095	56758	muscleblind-like 1 (Drosophila)	NA	-3.79	1.51E-04	6.62E-03	6.74	7.72	-0.98	-1.97	
858	1443558_s_at	AU040402	103466	5'-nucleotidase domain containing 3	Nt5dc3	3.28	1.03E-03	3.04E-02	7.56	6.70	0.86	1.81	RIKEN cDNA C630002B14 gene (5apos-nucleotidase domain containing 3)
859	1443579_s_at	AI957118	97998	DEP domain containing 6	Depdc6	-3.52	4.28E-04	1.51E-02	4.26	5.22	-0.96	-1.95	
860	1443777_at	BB343867	NA	NA	NA	-4.91	9.20E-07	8.74E-05	6.23	7.54	-1.30	-2.47	1443777_at (Unknown)
861	1443833_at	BE945021	NA	Transcribed locus	NA	3.10	1.95E-03	4.91E-02	3.90	3.30	0.61	1.52	1443833_at (Unknown)
862	1443841_x_at	AV321904	227620	UDP-N-acteylglucosamine pyrophosphorylase 1-like 1	Uap1l1	3.44	5.78E-04	1.93E-02	4.26	3.51	0.75	1.68	
863	1443889_at	AI789751	105892	RIKEN cDNA 9030619P08 gene	9030619P08Rik	-3.39	7.08E-04	2.25E-02	2.98	3.54	-0.56	-1.47	1443889_at (Unknown)
864	1444009_at	AV217868	213391	Ras association (RalGDS/AF-6) domain family 4	Rassf4	-4.98	6.40E-07	6.39E-05	9.37	10.50	-1.12	-2.17	
865	1444016_at	BE331865	16952	annexin A1	Anxa1	5.06	4.26E-07	4.47E-05	6.18	4.75	1.42	2.68	
866	1444073_at	AV352375	100047419 /// 17132	avian musculoaponeurotic fibrosarcoma (v-maf) AS42 oncogene homolog /// similar to c-Maf long form	LOC100047419 /// Maf	-5.12	3.11E-07	3.36E-05	4.71	6.14	-1.43	-2.70	
867	1444138_at	BB139766	244209	cytochrome P450, family 2, subfamily r, polypeptide 1	Cyp2r1	-3.17	1.52E-03	4.06E-02	3.56	4.28	-0.72	-1.65	
868	1444176_at	AV204216	242341	ATPase, H+ transporting, lysosomal V0 subunit D2	Atp6v0d2	5.35	8.78E-08	1.12E-05	12.10	10.90	1.22	2.32	
869	1444378_at	BQ033298	104318	12 days embryo spinal ganglion cDNA, RIKEN full-length enriched library, clone:D130001H05 product:unclassifiable, full insert sequence	NA	3.71	2.08E-04	8.60E-03	2.90	2.50	0.40	1.32	
870	1444400_at	BG069875	19645	retinoblastoma 1	Rb1	-5.61	2.01E-08	3.09E-06	3.25	4.50	-1.25	-2.39	
871	1444632_at	BF658882	408064	cDNA sequence BC064078	BC064078	-3.18	1.46E-03	3.95E-02	5.18	6.10	-0.91	-1.88	1444632_at (Unknown)
872	1444692_at	BB295954	30919	EST AI316844	AI316844	3.45	5.68E-04	1.90E-02	4.29	3.53	0.76	1.69	
873	1444706_at	BB527432	233235	Neuron navigator 2	Nav2	3.10	1.95E-03	4.90E-02	3.16	2.75	0.41	1.33	
874	1445363_at	BB451286	77994	RIKEN cDNA 2810055G20 gene	2810055G20Rik	-5.53	3.28E-08	4.75E-06	3.78	5.21	-1.42	-2.68	RIKEN cDNA 2810055G20 gene (unknown)
875	1445420_at	BM238940	17260	7 days neonate cerebellum cDNA, RIKEN full-length enriched library, clone:A730043F23 product:hypothetical protein, full insert sequence	NA	-4.23	2.31E-05	1.41E-03	4.18	5.32	-1.15	-2.21	
876	1445532_at	BB081552	244281	Myosin XVI	Myo16	3.38	7.33E-04	2.31E-02	3.35	2.85	0.50	1.41	RIKEN cDNA C230040D10 gene (Myosin-XVI (Unconventional myosin-16))
877	1445626_at	AI426376	16854	lectin, galactose binding, soluble 3	NA	7.47	7.77E-14	3.47E-11	9.39	7.66	1.72	3.31	
878	1445671_at	BG228218	72836	Transcribed locus	NA	-3.24	1.21E-03	3.40E-02	5.28	6.21	-0.93	-1.90	RIKEN cDNA 2810458H16 gene (protection of telomeres 1B)
879	1445831_at	BG069906	328425	deleted in lymphocytic leukemia, 2	NA	-3.45	5.56E-04	1.87E-02	4.92	5.90	-0.99	-1.98	
880	1445966_at	BG075586	16004	Transcribed locus	NA	3.20	1.36E-03	3.74E-02	3.66	3.10	0.56	1.47	
881	1445984_at	BB027193	18647	Adult male pituitary gland cDNA, RIKEN full-length enriched library, clone:5330436L08 product:unclassifiable, full insert sequence	NA	-4.16	3.15E-05	1.81E-03	3.84	4.90	-1.06	-2.09	
882	1446055_at	BB282903	NA	NA	NA	3.98	6.89E-05	3.51E-03	5.75	4.63	1.12	2.17	1446055_at (Unknown)
883	1446617_at	BB224265	20452	ST8 alpha-N-acetyl-neuraminide alpha-2,8-sialyltransferase 4	NA	3.49	4.81E-04	1.67E-02	3.60	3.01	0.59	1.50	
884	1446627_at	BB556211	56274	serine/threonine kinase 3 (Ste20, yeast homolog)	NA	-3.31	9.33E-04	2.81E-02	3.08	3.67	-0.59	-1.50	
885	1446736_at	BM198618	21372	transducin (beta)-like 1 X-linked	NA	5.09	3.52E-07	3.76E-05	3.01	2.44	0.57	1.49	
886	1446834_at	AV367948	13032	Transcribed locus	NA	-8.18	2.79E-16	1.85E-13	4.05	6.21	-2.16	-4.46	

	A	B	C	D	E	F	G	H	I	J	K	L	M
887	1447063_at	BB273020	74211	septin 14	sept14	4.19	2.81E-05	1.64E-03	4.60	3.62	0.98	1.97	RIKEN cDNA 1700017B05 gene (Uncharacterized protein C15orf39 homolog)
888	1447229_x_at	BM248411	228356	RIKEN cDNA 1110051M20 gene	1110051M20Rik	4.15	3.38E-05	1.92E-03	4.08	3.25	0.83	1.78	
889	1447272_s_at	BM249532	11982	ATPase, class V, type 10A	Atp10a	-4.42	9.76E-06	6.79E-04	3.60	4.67	-1.07	-2.10	
890	1447449_at	BE983114	NA	NA	NA	3.11	1.85E-03	4.71E-02	5.25	4.40	0.85	1.80	1447449_at (Unknown)
891	1447766_x_at	AV003249	632329 /// 67803	LIM domain containing 2 /// similar to epithelial protein lost in neoplasm	Limd2 /// LOC632329	-7.28	3.27E-13	1.33E-10	2.97	4.54	-1.57	-2.97	RIKEN cDNA 0610025L06 gene (LIM domain-containing protein 2)
892	1447813_x_at	AV247013	20491	src-like adaptor	Sla	-3.88	1.04E-04	4.90E-03	3.43	4.29	-0.86	-1.82	
893	1447838_x_at	BB359162	78798	echinoderm microtubule associated protein like 4	Eml4	-6.72	1.85E-11	5.42E-09	2.09	2.58	-0.49	-1.40	
894	1447839_x_at	AV378441	11535	adrenomedullin	Adm	7.45	9.59E-14	4.19E-11	4.24	2.82	1.42	2.68	
895	1447849_s_at	AV323441	17132	avian musculoaponeurotic fibrosarcoma (v-maf) AS42 oncogene homolog	Maf	-5.38	7.48E-08	9.65E-06	9.60	10.80	-1.22	-2.33	1447849_s_at (Transcription factor Maf (Proto-oncogene c-maf))
896	1447900_x_at	BB022415	100048085 /// 67464	ectonucleoside triphosphate diphosphohydrolase 4 /// similar to ectonucleoside triphosphate diphosphohydrolase 4	Entpd4 /// LOC100048085	-5.28	1.28E-07	1.55E-05	3.01	4.04	-1.03	-2.04	
897	1448021_at	AA266723	NA	Transcribed locus	NA	6.98	2.94E-12	1.03E-09	8.79	7.09	1.70	3.24	1448021_at (Unknown)
898	1448123_s_at	NM_009369	21810	transforming growth factor, beta induced	Tgfbi	-9.16	5.14E-20	3.93E-17	9.30	11.40	-2.12	-4.36	
899	1448124_at	NM_010368	110006	glucuronidase, beta	Gusb	3.16	1.58E-03	4.18E-02	12.70	12.00	0.71	1.64	
900	1448130_at	NM_010191	14137	farnesyl diphosphate farnesyl transferase 1	Fdft1	5.16	2.44E-07	2.76E-05	7.97	6.64	1.33	2.51	
901	1448134_at	NM_013753	27355	cDNA sequence X99384	X99384	-3.15	1.65E-03	4.33E-02	5.00	5.91	-0.90	-1.87	cDNA sequence X99384 (Paladin)
902	1448158_at	BI788645	20969	syndecan 1	Sdc1	9.67	0.00E+00	0.00E+00	8.26	5.70	2.56	5.88	
903	1448167_at	NM_010511	15979	interferon gamma receptor 1	Ifngr1	-3.90	9.73E-05	4.66E-03	9.37	10.20	-0.87	-1.83	
904	1448175_at	NM_010119	13660	EH-domain containing 1	Ehd1	-5.55	2.92E-08	4.27E-06	7.36	8.71	-1.34	-2.54	
905	1448182_a_at	NM_009846	12484	CD24a antigen	Cd24a	4.51	6.39E-06	4.72E-04	8.16	7.03	1.12	2.18	
906	1448213_at	NM_010730	16952	annexin A1	Anxa1	4.98	6.43E-07	6.40E-05	15.00	14.40	0.55	1.47	
907	1448226_at	NM_009104	20135	ribonucleotide reductase M2	Rrm2	-5.06	4.12E-07	4.34E-05	5.29	6.70	-1.42	-2.67	
908	1448229_s_at	NM_009829	12444	cyclin D2	Ccnd2	-5.17	2.28E-07	2.59E-05	4.82	6.28	-1.45	-2.74	
909	1448303_at	NM_053110	93695	glycoprotein (transmembrane) nmb	Gpnmb	4.19	2.81E-05	1.64E-03	14.50	13.90	0.60	1.52	
910	1448306_at	NM_010907	18035	nuclear factor of kappa light chain gene enhancer in B-cells inhibitor, alpha	Nfkbia	-3.85	1.20E-04	5.53E-03	8.76	9.63	-0.87	-1.83	
911	1448310_at	NM_019987	56542	intestinal cell kinase	Ick	-3.16	1.56E-03	4.14E-02	4.08	4.92	-0.83	-1.78	
912	1448314_at	NM_007659	12534	cell division cycle 2 homolog A (S. pombe)	Cdc2a	-3.76	1.69E-04	7.23E-03	7.18	8.11	-0.93	-1.91	
913	1448325_at	NM_008654	17872	myeloid differentiation primary response gene 116	Myd116	3.15	1.61E-03	4.25E-02	7.79	6.99	0.80	1.74	
914	1448330_at	NM_010358	14862	glutathione S-transferase, mu 1	Gstm1	6.78	1.22E-11	3.70E-09	10.80	9.31	1.52	2.87	
915	1448352_at	BB705823	269593	leucine zipper protein 1	Luzp1	-4.63	3.65E-06	2.93E-04	7.86	8.95	-1.09	-2.13	
916	1448399_at	NM_025816	52440	Tax1 (human T-cell leukemia virus type I) binding protein 1	Tax1bp1	3.29	1.00E-03	2.97E-02	11.40	10.60	0.75	1.69	
917	1448405_a_at	BC010712	58521	EP300 interacting inhibitor of differentiation 1	Eid1	-4.14	3.52E-05	1.98E-03	10.30	11.20	-0.95	-1.93	
918	1448406_at	BC010712	58521	EP300 interacting inhibitor of differentiation 1	Eid1	-3.39	6.96E-04	2.22E-02	11.00	11.80	-0.80	-1.74	
919	1448470_at	NM_019395	14121	fructose bisphosphatase 1	Fbp1	-3.90	9.79E-05	4.68E-03	5.05	6.16	-1.11	-2.16	
920	1448471_a_at	NM_007796	13024	cytotoxic T lymphocyte-associated protein 2 alpha	Ctla2a	-5.00	5.59E-07	5.65E-05	6.38	7.69	-1.31	-2.48	
921	1448475_at	NM_133859	99543	olfactomedin-like 3	Olfml3	-5.19	2.07E-07	2.39E-05	6.75	8.07	-1.32	-2.49	
922	1448552_s_at	NM_025864	66950	RIKEN cDNA 2310028N02 gene	2310028N02Rik	3.26	1.11E-03	3.19E-02	9.15	8.42	0.73	1.66	RIKEN cDNA 2310028N02 gene (Transmembrane protein C1orf75 homolog)
923	1448568_a_at	NM_015747	20515	solute carrier family 20, member 1	Slc20a1	5.57	2.48E-08	3.72E-06	9.14	7.86	1.28	2.42	
924	1448575_at	AI573431	16197	interleukin 7 receptor	Il7r	6.62	3.53E-11	9.77E-09	12.90	11.40	1.46	2.76	
925	1448576_at	AI573431	16197	interleukin 7 receptor	Il7r	6.83	8.22E-12	2.59E-09	11.90	10.30	1.54	2.91	
926	1448595_a_at	NM_009052	19716	brain expressed gene 1	Bex1	13.15	0.00E+00	0.00E+00	9.91	6.67	3.24	9.46	
927	1448596_at	BG069516	102857	solute carrier family 6 (neurotransmitter transporter, creatine), member 8	Slc6a8	6.97	3.21E-12	1.11E-09	10.50	8.98	1.56	2.94	
928	1448605_at	NM_007484	11853	ras homolog gene family, member C	Rhoc	3.32	9.11E-04	2.75E-02	10.00	9.31	0.74	1.67	
929	1448609_at	BC005644	22117	thiosulfate sulfurtransferase, mitochondrial	Tst	7.24	4.34E-13	1.73E-10	7.73	5.78	1.95	3.87	
930	1448656_at	NM_007581	12297	calcium channel, voltage-dependent, beta 3 subunit	Cacnb3	-3.13	1.78E-03	4.57E-02	3.27	3.89	-0.62	-1.53	
931	1448663_s_at	NM_138656	192156	mevalonate (diphospho) decarboxylase	Mvd	3.91	9.33E-05	4.51E-03	5.59	4.51	1.08	2.12	
932	1448731_at	NM_008348	16154	interleukin 10 receptor, alpha	Il10ra	-4.53	5.89E-06	4.40E-04	7.24	8.35	-1.11	-2.16	
933	1448734_at	BB332449	12870	ceruloplasmin	Cp	-3.95	7.79E-05	3.90E-03	5.22	6.34	-1.12	-2.18	
934	1448747_at	AF441120	67731	F-box protein 32	Fbxo32	4.37	1.25E-05	8.40E-04	7.93	6.81	1.12	2.17	
935	1448748_at	AF181829	56193	pleckstrin	Plek	3.92	8.89E-05	4.34E-03	14.10	13.40	0.68	1.60	
936	1448749_at	AF181829	56193	pleckstrin	Plek	4.19	2.77E-05	1.63E-03	13.70	12.80	0.84	1.79	
937	1448771_a_at	NM_010239	14319	ferritin heavy chain 1	Fth1	6.54	6.19E-11	1.62E-08	15.90	15.70	0.20	1.15	
938	1448788_at	AF004023	17470	Cd200 antigen	Cd200	8.05	8.88E-16	5.48E-13	5.92	3.87	2.05	4.14	
939	1448847_at	NM_012032	26943	serine incorporator 3	Serinc3	-3.83	1.29E-04	5.90E-03	7.30	8.23	-0.94	-1.92	
940	1448862_at	NM_010494	15896	intercellular adhesion molecule 2	Icam2	-3.30	9.68E-04	2.88E-02	6.33	7.21	-0.89	-1.85	
941	1448890_at	NM_008452	16598	Kruppel-like factor 2 (lung)	Klf2	-5.30	1.15E-07	1.41E-05	6.49	7.86	-1.37	-2.59	
942	1448891_at	BC016551	80891	macrophage scavenger receptor 2	Msr2	-5.34	9.50E-08	1.21E-05	9.46	10.70	-1.21	-2.31	
943	1448899_s_at	BC003738	19362	RAD51 associated protein 1	Rad51ap1	-5.27	1.36E-07	1.63E-05	2.69	3.48	-0.79	-1.73	
944	1448908_at	NM_080555	67916	phosphatidic acid phosphatase type 2B	Ppap2b	11.89	0.00E+00	0.00E+00	8.81	5.72	3.09	8.53	
945	1448909_a_at	NM_017404	27393	mitochondrial ribosomal protein L39	Mrpl39	-3.87	1.09E-04	5.12E-03	9.07	9.93	-0.87	-1.83	
946	1448919_at	NM_025422	66205	CD302 antigen	Cd302	-3.37	7.53E-04	2.37E-02	9.01	9.76	-0.76	-1.69	
947	1448929_at	NM_028784	74145	coagulation factor XIII, A1 subunit	F13a1	-3.89	1.02E-04	4.82E-03	9.19	10.10	-0.87	-1.83	
948	1448942_at	NM_025331	66066	guanine nucleotide binding protein (G protein), gamma 11	Gng11	4.16	3.20E-05	1.83E-03	10.60	9.65	0.93	1.91	
949	1448955_s_at	NM_012061	27062	Ca<2+>dependent activator protein for secretion	Cadps	4.58	4.75E-06	3.66E-04	6.46	5.15	1.30	2.46	
950	1449005_at	NM_030696	80879	solute carrier family 16 (monocarboxylic acid transporters), member 3	Slc16a3	6.63	3.28E-11	9.13E-09	8.69	7.08	1.62	3.07	
951	1449009_at	NM_011579	100039796 /// 21822	T-cell specific GTPase /// hypothetical protein LOC100039796	LOC100039796 /// Tgtp	-3.75	1.80E-04	7.60E-03	10.80	11.70	-0.88	-1.84	
952	1449078_at	NM_018784	54613	ST3 beta-galactoside alpha-2,3-sialyltransferase 6	St3gal6	-7.93	2.11E-15	1.24E-12	4.05	6.15	-2.10	-4.28	
953	1449079_s_at	NM_018784	54613	ST3 beta-galactoside alpha-2,3-sialyltransferase 6	St3gal6	-6.40	1.55E-10	3.86E-08	4.15	5.87	-1.73	-3.31	
954	1449110_at	BC018275	11852	ras homolog gene family, member B	Rhob	5.81	6.37E-09	1.08E-06	10.80	9.53	1.31	2.47	
955	1449124_at	NM_016846	19731	ral guanine nucleotide dissociation stimulator,-like 1	Rgl1	4.40	1.09E-05	7.46E-04	9.12	8.13	1.00	2.00	
956	1449141_at	BG070068	74202	filamin binding LIM protein 1	Fblim1	4.10	4.11E-05	2.26E-03	7.67	6.60	1.07	2.11	
957	1449146_at	NM_010929	18132	Notch gene homolog 4 (Drosophila)	Notch4	-3.36	7.72E-04	2.41E-02	3.10	3.71	-0.61	-1.52	
958	1449151_at	NM_008795	18557	PCTAIRE-motif protein kinase 3	Pctk3	6.17	7.02E-10	1.51E-07	10.70	9.30	1.38	2.60	
959	1449153_at	BC019135	17381	matrix metallopeptidase 12	Mmp12	8.52	0.00E+00	0.00E+00	13.40	11.50	1.83	3.55	
960	1449168_a_at	BC003735	11641	A kinase (PRKA) anchor protein 2	Akap2	6.50	7.83E-11	2.01E-08	5.90	4.17	1.73	3.32	
961	1449175_at	NM_008152	14744	G-protein coupled receptor 65	Gpr65	-4.81	1.51E-06	1.36E-04	10.80	11.90	-1.13	-2.19	
962	1449176_a_at	NM_007832	13178	deoxycytidine kinase	Dck	-3.59	3.31E-04	1.23E-02	10.60	11.40	-0.83	-1.78	
963	1449193_at	NM_009690	11801	CD5 antigen-like	Cd5l	-4.92	8.77E-07	8.42E-05	7.75	8.91	-1.16	-2.24	
964	1449195_s_at	BC019961	66102	chemokine (C-X-C motif) ligand 16	Cxcl16	-3.50	4.66E-04	1.63E-02	10.00	10.80	-0.79	-1.73	
965	1449221_a_at	NM_133626	81910	ribosome binding protein 1	Rrbp1	3.32	8.85E-04	2.69E-02	9.07	8.31	0.75	1.68	
966	1449227_at	NM_009890	12642	cholesterol 25-hydroxylase	Ch25h	-10.44	1.66E-25	1.39E-22	4.37	7.09	-2.71	-6.57	
967	1449249_at	NM_018764	54216	protocadherin 7	Pcdh7	-3.59	3.32E-04	1.24E-02	9.12	9.93	-0.80	-1.75	
968	1449282_at	BC027102	58861	cysteinyl leukotriene receptor 1	Cysltr1	-3.49	4.77E-04	1.66E-02	8.61	9.41	-0.79	-1.73	
969	1449305_at	NM_007972	14058	coagulation factor X	F10	3.76	1.71E-04	7.27E-03	7.62	6.64	0.98	1.98	
970	1449324_at	BM234652	50527	ERO1-like (S. cerevisiae)	Ero1l	3.98	6.96E-05	3.54E-03	9.49	8.60	0.89	1.85	1449324_at (Endoplasmic oxidoreductin-1-like protein) (ERO1-L)

	A	B	C	D	E	F	G	H	I	J	K	L	M
971	1449363_at	BC019946	11910	activating transcription factor 3	Atf3	3.63	2.82E-04	1.09E-02	12.70	11.90	0.82	1.77	
972	1449379_at	NM_010612	16542	kinase insert domain protein receptor	Kdr	3.64	2.76E-04	1.08E-02	7.38	6.40	0.98	1.97	
973	1449399_a_at	BC011437	16176	interleukin 1 beta	Il1b	-6.02	1.77E-09	3.49E-07	5.79	7.42	-1.63	-3.09	
974	1449490_at	AF072249	17193	methyl-CpG binding domain protein 4	Mbd4	-4.76	1.91E-06	1.66E-04	3.12	4.07	-0.96	-1.94	
975	1449519_at	NM_007836	13197	growth arrest and DNA-damage-inducible 45 alpha	Gadd45a	10.74	0.00E+00	0.00E+00	10.80	8.38	2.43	5.38	
976	1449530_at	NM_032000	100044341 /// 83925	trichorhinophalangeal syndrome I (human) /// similar to Trps1 protein	LOC100044341 /// Trps1	-3.57	3.56E-04	1.31E-02	7.23	8.11	-0.88	-1.84	
977	1449553_at	AK009389	67149	Na+/K+ transporting ATPase interacting 1	Nkain1	6.16	7.43E-10	1.59E-07	8.19	6.61	1.57	2.98	RIKEN cDNA 2610200G18 gene (Sodium/potassium-transporting ATPase subunit beta-1-interacting protein 1 (Na(+)/K(+)-transporting ATPase subunit beta-1-interacting protein 1) (Protein FAM77C))
978	1449620_s_at	AW125421	28001	DNA segment, Chr 16, Wayne State University 65, expressed	D16Wsu65e	-3.38	7.18E-04	2.28E-02	6.00	6.94	-0.94	-1.91	
979	1449640_at	AI604175	170743	toll-like receptor 7	Tlr7	-3.12	1.80E-03	4.61E-02	6.56	7.39	-0.82	-1.77	
980	1449670_x_at	AW546472	100044979 /// 83924	G protein-coupled receptor 137B /// similar to Gpr137b protein	Gpr137b /// LOC100044979	5.32	1.03E-07	1.29E-05	10.00	8.85	1.18	2.27	
981	1449773_s_at	AI323528	17873	growth arrest and DNA-damage-inducible 45 beta	Gadd45b	5.68	1.38E-08	2.19E-06	9.70	8.44	1.27	2.41	
982	1449799_s_at	AA516617	67451	plakophilin 2	Pkp2	3.52	4.28E-04	1.51E-02	4.44	3.63	0.81	1.75	
983	1449815_a_at	NM_024186	66970	single-stranded DNA binding protein 2	Ssbp2	-3.58	3.46E-04	1.28E-02	6.40	7.35	-0.95	-1.94	
984	1449852_a_at	NM_133838	98878	EH-domain containing 4	Ehd4	-3.16	1.56E-03	4.14E-02	11.10	11.80	-0.75	-1.68	
985	1449858_at	NM_019388	12524	CD86 antigen	Cd86	-3.34	8.32E-04	2.56E-02	9.09	9.84	-0.75	-1.68	
986	1449931_at	NM_026252	67579	cytoplasmic polyadenylation element binding protein 4	Cpeb4	4.79	1.69E-06	1.50E-04	6.88	5.54	1.35	2.54	
987	1449954_at	NM_008317	15586 /// 56441	hyaluronoglucosaminidase 1 /// N-acetyltransferase 6	Hyal1 /// Nat6	5.89	3.85E-09	6.89E-07	9.14	7.79	1.36	2.56	
988	1449976_a_at	NM_022320	64095	G protein-coupled receptor 35	Gpr35	-3.37	7.59E-04	2.38E-02	6.42	7.32	-0.90	-1.86	
989	1449984_at	NM_009140	20310	chemokine (C-X-C motif) ligand 2	Cxcl2	3.86	1.15E-04	5.34E-03	6.55	5.45	1.10	2.14	
990	1450006_at	NM_019744	27057 /// 627557	nuclear receptor coactivator 4 /// predicted gene, EG627557	EG627557 /// Ncoa4	3.27	1.09E-03	3.15E-02	10.20	9.51	0.73	1.65	
991	1450008_a_at	NM_007614	12387	catenin (cadherin associated protein), beta 1	Ctnnb1	4.60	4.31E-06	3.38E-04	12.00	10.90	1.05	2.07	
992	1450020_at	BC012653	13051	chemokine (C-X3-C) receptor 1	Cx3cr1	-5.19	2.08E-07	2.39E-05	5.95	7.35	-1.40	-2.65	
993	1450061_at	BM120053	13803	ectodermal-neural cortex 1	Enc1	-3.43	5.98E-04	1.97E-02	8.20	8.99	-0.79	-1.73	
994	1450070_s_at	BF456404	18479	p21 (CDKN1A)-activated kinase 1	Pak1	-3.30	9.62E-04	2.87E-02	6.13	7.03	-0.91	-1.87	
995	1450082_s_at	BG966751	104156	ets variant gene 5	Etv5	-4.74	2.18E-06	1.88E-04	7.79	8.90	-1.12	-2.17	
996	1450129_a_at	NM_018821	54607	suppressor of cytokine signaling 6	Socs6	3.93	8.46E-05	4.18E-03	10.60	9.73	0.88	1.84	
997	1450231_a_at	BF137345	11798	baculoviral IAP repeat-containing 4	Birc4	-3.41	6.49E-04	2.11E-02	3.43	4.17	-0.74	-1.67	
998	1450267_at	NM_133212	170744	toll-like receptor 8	Tlr8	-3.66	2.51E-04	1.00E-02	8.38	9.22	-0.84	-1.79	
999	1450318_a_at	NM_008773	18442	purinergic receptor P2Y, G-protein coupled 2	P2ry2	3.48	5.06E-04	1.73E-02	7.26	6.32	0.94	1.92	
1000	1450323_at	NM_027488	70630	RIKEN cDNA 5730493B19 gene	5730493B19Rik	-4.56	5.19E-06	3.96E-04	2.21	2.56	-0.35	-1.27	RIKEN cDNA 5730493B19 gene (unknown)
1001	1450330_at	NM_010548	16153	interleukin 10	Il10	-6.22	4.97E-10	1.11E-07	3.64	5.22	-1.57	-2.97	
1002	1450382_at	L28176	18016	neurofibromatosis 2	Nf2	3.46	5.45E-04	1.84E-02	7.85	6.98	0.88	1.84	
1003	1450383_at	AF425607	16835	low density lipoprotein receptor	Ldlr	8.21	2.22E-16	1.49E-13	7.61	5.38	2.23	4.68	
1004	1450391_a_at	NM_011844	23945	monoglyceride lipase	Mgll	5.87	4.40E-09	7.72E-07	6.27	4.64	1.63	3.09	
1005	1450407_a_at	AF022957	11737	acidic (leucine-rich) nuclear phosphoprotein 32 family, member A	Anp32a	-3.69	2.20E-04	9.01E-03	8.11	8.97	-0.86	-1.81	
1006	1450449_a_at	NM_021430	75695	Rab interacting lysosomal protein-like 1	Rilpl1	3.82	1.36E-04	6.13E-03	8.28	7.36	0.93	1.90	RIKEN cDNA 2900002H16 gene (UPF0475 protein)
1007	1450488_at	AF281075	56221	chemokine (C-C motif) ligand 24	Ccl24	-3.52	4.30E-04	1.52E-02	4.41	5.39	-0.98	-1.97	
1008	1450495_a_at	AF039026	27007	killer cell lectin-like receptor subfamily K, member 1	Klrk1	-3.39	6.92E-04	2.21E-02	8.24	9.02	-0.78	-1.72	
1009	1450641_at	M24849	22352	vimentin	Vim	4.08	4.60E-05	2.49E-03	14.30	13.60	0.66	1.58	
1010	1450644_at	M58566	12192	zinc finger protein 36, C3H type-like 1	Zfp36l1	-3.87	1.09E-04	5.12E-03	11.40	12.30	-0.91	-1.88	
1011	1450646_at	NM_020010	13121	cytochrome P450, family 51	Cyp51	6.11	1.00E-09	2.10E-07	8.69	7.21	1.48	2.78	
1012	1450652_at	NM_007802	13038	cathepsin K	Ctsk	8.19	2.22E-16	1.49E-13	7.53	5.30	2.23	4.69	
1013	1450684_at	NM_007960	14009	ets variant gene 1	Etv1	-3.29	9.99E-04	2.96E-02	6.83	7.67	-0.85	-1.80	
1014	1450716_at	D67076	11504	a disintegrin-like and metallopeptidase (reprolysin type) with thrombospondin type 1 motif, 1	Adamts1	7.59	3.09E-14	1.43E-11	6.82	4.74	2.08	4.22	
1015	1450717_at	NM_007447	11727	angiogenin, ribonuclease, RNase A family, 5	Ang	-5.53	3.22E-08	4.69E-06	4.73	6.28	-1.54	-2.92	
1016	1450718_at	NM_018825	23921	SH2B adaptor protein 2	Sh2b2	3.48	4.99E-04	1.72E-02	6.83	5.86	0.98	1.97	
1017	1450780_s_at	X58380	15364	high mobility group AT-hook 2	Hmga2	5.50	3.87E-08	5.46E-06	9.70	8.47	1.23	2.34	
1018	1450781_at	X58380	15364	high mobility group AT-hook 2	Hmga2	5.13	2.85E-07	3.13E-05	9.37	8.21	1.16	2.23	
1019	1450826_a_at	NM_011315	20210	serum amyloid A 3	Saa3	-3.66	2.48E-04	9.93E-03	8.77	9.60	-0.83	-1.78	
1020	1450829_at	NM_009397	21929	tumor necrosis factor, alpha-induced protein 3	Tnfaip3	4.39	1.15E-05	7.83E-04	7.44	6.26	1.18	2.27	
1021	1450862_at	AV310220	19366	RAD54 like (S. cerevisiae)	Rad54l	-4.07	4.64E-05	2.51E-03	4.25	5.37	-1.11	-2.16	
1022	1450868_at	BF468249	52120	heparan-alpha-glucosaminide N-acetyltransferase	Hgsnat	-3.65	2.59E-04	1.03E-02	8.16	9.01	-0.85	-1.80	
1023	1450876_at	AI987976	100048018 /// 12628	complement component factor h /// similar to complement component factor H	Cfh /// LOC100048018	-5.47	4.40E-08	6.08E-06	5.85	7.34	-1.48	-2.80	
1024	1450881_s_at	AK010724	83924	G protein-coupled receptor 137B	Gpr137b	4.96	6.96E-07	6.89E-05	11.40	10.30	1.13	2.19	
1025	1450882_s_at	AK010724	100044979 /// 664862 /// 83924	G protein-coupled receptor 137B /// G protein-coupled receptor 137B, pseudogene /// similar to Gpr137b protein	Gpr137b /// Gpr137b-ps /// LOC100044979	5.41	6.16E-08	8.11E-06	11.30	10.10	1.23	2.34	
1026	1450920_at	AK013312	12442	cyclin B2	Ccnb2	-3.56	3.64E-04	1.33E-02	5.49	6.50	-1.01	-2.02	
1027	1450971_at	AK010420	17873	growth arrest and DNA-damage-inducible 45 beta	Gadd45b	6.62	3.68E-11	1.00E-08	11.20	9.74	1.50	2.82	
1028	1450976_at	AI987929	17988	N-myc downstream regulated gene 1	Ndrg1	4.66	3.13E-06	2.58E-04	11.20	10.20	1.06	2.08	
1029	1450977_s_at	AI987929	17988	N-myc downstream regulated gene 1	Ndrg1	4.86	1.20E-06	1.10E-04	10.50	9.44	1.08	2.12	
1030	1450997_at	AV173139	98267	serine/threonine kinase 17b (apoptosis-inducing)	Stk17b	-3.46	5.43E-04	1.83E-02	8.31	9.11	-0.79	-1.73	
1031	1451013_at	BB541271	71279	solute carrier family 29 (nucleoside transporters), member 3	Slc29a3	4.83	1.39E-06	1.26E-04	9.35	8.26	1.09	2.12	
1032	1451053_a_at	AV313203	17245	transformed mouse 3T3 cell double minute 1	Mdm1	-3.69	2.20E-04	9.01E-03	3.97	4.93	-0.96	-1.95	
1033	1451105_at	BC024141	226841	vasohibin 2	Vash2	3.14	1.71E-03	4.46E-02	6.40	5.50	0.90	1.86	RIKEN cDNA B130052G07 gene (Protein angel homolog 2.)
1034	1451122_at	BC004801	319554	isopentenyl-diphosphate delta isomerase	Idi1	6.06	1.35E-09	2.77E-07	8.80	7.36	1.44	2.72	
1035	1451201_s_at	BC010331	107702	ribonuclease/angiogenin inhibitor 1	Rnh1	6.10	1.06E-09	2.21E-07	13.90	12.70	1.21	2.32	
1036	1451206_s_at	BC007144	227929	pleckstrin homology, Sec7 and coiled-coil domains, binding protein	Pscdbp	-7.61	2.75E-14	1.29E-11	8.23	9.99	-1.76	-3.39	
1037	1451230_a_at	BC007478	22381	WW domain binding protein 5	Wbp5	3.34	8.52E-04	2.62E-02	12.80	12.00	0.75	1.68	
1038	1451246_s_at	BC003261	20877	aurora kinase B	Aurkb	-3.57	3.62E-04	1.33E-02	4.13	5.08	-0.95	-1.94	
1039	1451263_a_at	BC002148	11770	fatty acid binding protein 4, adipocyte	Fabp4	11.59	0.00E+00	0.00E+00	12.70	10.20	2.50	5.67	
1040	1451264_at	BC019939	319710	FERM domain containing 6	Frmd6	-3.68	2.37E-04	9.58E-03	7.27	8.17	-0.90	-1.87	
1041	1451288_s_at	BC010330	67884	RIKEN cDNA 1810043G02 gene	1810043G02Rik	-4.92	8.58E-07	8.27E-05	5.46	6.84	-1.37	-2.59	RIKEN cDNA 1810043G02 gene (unknown)
1042	1451306_at	BC006933	217946	cell division cycle associated 7 like	Cdca7l	4.51	6.41E-06	4.73E-04	8.80	7.76	1.05	2.07	
1043	1451310_a_at	J02583	13039	cathepsin L	Ctsl	4.41	1.05E-05	7.24E-04	15.50	15.20	0.28	1.22	
1044	1451341_s_at	BB208266	407243	transmembrane protein 189	Tmem189	3.46	5.42E-04	1.83E-02	10.70	9.95	0.78	1.71	expressed sequence AI840826 (ubiquitin-conjugating enzyme variant Kua)
1045	1451382_at	BC025169	69065	ChaC, cation transport regulator-like 1 (E. coli)	Chac1	3.33	8.80E-04	2.68E-02	6.80	5.86	0.93	1.91	RIKEN cDNA 1810008K03 gene (Cation transport regulator-like protein 1)
1046	1451385_at	BC010826	70186	RIKEN cDNA 2310056P07 gene	2310056P07Rik	3.12	1.78E-03	4.58E-02	10.60	9.90	0.70	1.62	RIKEN cDNA 2310056P07 gene (E2-induced gene 5 protein homolog)
1047	1451386_at	BC027279	233016	biliverdin reductase B (flavin reductase (NADPH))	Blvrb	-3.09	1.98E-03	4.97E-02	10.50	11.20	-0.71	-1.64	
1048	1451411_at	BC020004	64297	G protein-coupled receptor, family C, group 5, member B	Gprc5b	5.19	2.08E-07	2.39E-05	3.09	2.47	0.61	1.53	
1049	1451418_a_at	BC023083	211949	splA/ryanodine receptor domain and SOCS box containing 4	Spsb4	7.01	2.44E-12	8.65E-10	6.84	4.91	1.94	3.83	
1050	1451419_at	BC023083	211949	splA/ryanodine receptor domain and SOCS box containing 4	Spsb4	6.24	4.51E-10	1.01E-07	5.96	4.28	1.68	3.21	
1051	1451421_a_at	BC006914	66049	rogdi homolog (Drosophila)	Rogdi	-3.15	1.63E-03	4.30E-02	6.74	7.56	-0.82	-1.76	
1052	1451457_at	AB016248	235293	sterol-C5-desaturase (fungal ERG3, delta-5-desaturase) homolog (S. cerevisae)	Sc5d	5.66	1.51E-08	2.37E-06	8.65	7.29	1.36	2.57	
1053	1451461_a_at	BC008184	11676	aldolase 3, C isoform	Aldoc	3.62	2.95E-04	1.13E-02	7.86	6.94	0.92	1.89	
1054	1451474_a_at	BC022679	52552	poly (ADP-ribose) polymerase family, member 8	Parp8	-3.99	6.56E-05	3.40E-03	5.72	6.83	-1.11	-2.17	

	A	B	C	D	E	F	G	H	I	J	K	L	M
1055	1451486_at	BC006902	71706	solute carrier family 46, member 3	Slc46a3	-3.81	1.38E-04	6.18E-03	6.63	7.62	-0.99	-1.99	RIKEN cDNA 1200006F02 gene (Solute carrier family 46 member 3 precursor)
1056	1451506_at	BB280300	17260	myocyte enhancer factor 2C	Mef2c	-6.10	1.04E-09	2.16E-07	9.70	11.10	-1.39	-2.63	
1057	1451507_at	BB280300	17260	myocyte enhancer factor 2C	Mef2c	-6.31	2.72E-10	6.31E-08	8.31	9.76	-1.45	-2.74	
1058	1451518_at	BC021921	236193	zinc finger protein 709	Zfp709	3.19	1.43E-03	3.87E-02	7.48	6.64	0.84	1.79	cDNA sequence BC021921 (Zinc finger protein 14)
1059	1451528_at	BC025833	213989	transmembrane protein 82	Tmem82	4.46	8.31E-06	5.92E-04	5.17	4.01	1.15	2.23	cDNA sequence BC025833 (Transmembrane protein 82)
1060	1451558_at	AV338062	50754	F-box and WD-40 domain protein 7, archipelago homolog (Drosophila)	Fbxw7	3.78	1.58E-04	6.83E-03	8.13	7.19	0.93	1.91	
1061	1451563_at	AF396935	52614	EGF-like module containing, mucin-like, hormone receptor-like sequence 4	Emr4	-8.98	2.64E-19	1.98E-16	5.34	7.73	-2.39	-5.23	
1062	1451753_at	D86949	18845	plexin A2	Plxna2	3.25	1.16E-03	3.29E-02	6.91	6.00	0.91	1.87	
1063	1451798_at	M57525	16181	interleukin 1 receptor antagonist	Il1rn	9.02	0.00E+00	0.00E+00	12.30	10.30	2.00	3.99	
1064	1451803_a_at	U48800	22340	vascular endothelial growth factor B	Vegfb	4.55	5.29E-06	4.02E-04	7.89	6.72	1.17	2.25	
1065	1451819_at	BC021311	67263	zinc finger, SWIM domain containing 6	Zswim6	-3.70	2.14E-04	8.82E-03	5.62	6.66	-1.04	-2.06	
1066	1451852_at	AF067060	236219	2-cell-stage, variable group, member 3	Tcstv3	-5.88	4.15E-09	7.32E-07	4.82	6.46	-1.64	-3.11	
1067	1451895_a_at	BC004738	74754	24-dehydrocholesterol reductase	Dhcr24	5.95	2.69E-09	5.04E-07	6.85	5.19	1.67	3.17	
1068	1451986_s_at	BC027199	233328	leucine-rich repeat kinase 1	Lrrk1	-3.66	2.51E-04	1.00E-02	5.94	6.95	-1.01	-2.02	
1069	1452042_a_at	AV306255	70652	transmembrane protein 144	Tmem144	3.19	1.43E-03	3.89E-02	5.22	4.35	0.87	1.82	RIKEN cDNA 5730537D05 gene (Transmembrane protein 144)
1070	1452050_at	BG071931	227541	calcium/calmodulin-dependent protein kinase ID	Camk1d	-3.72	1.96E-04	8.17E-03	8.34	9.20	-0.85	-1.81	
1071	1452078_a_at	BG065264	18174	solute carrier family 11 (proton-coupled divalent metal ion transporters), member 2	Slc11a2	4.53	5.98E-06	4.45E-04	8.63	7.56	1.07	2.10	
1072	1452160_at	BB707122	99929	TCDD-inducible poly(ADP-ribose) polymerase	Tiparp	3.13	1.76E-03	4.55E-02	8.74	8.02	0.72	1.65	
1073	1452198_at	AF176524	30841	F-box and leucine-rich repeat protein 10	Fbxl10	-3.30	9.56E-04	2.86E-02	9.19	9.93	-0.74	-1.67	
1074	1452209_at	AV286396	227937	plakophilin 4	Pkp4	4.90	9.62E-07	9.09E-05	8.08	6.84	1.24	2.36	
1075	1452213_at	BM239615	21763	testis expressed gene 2	Tex2	3.33	8.58E-04	2.63E-02	9.38	8.64	0.74	1.68	
1076	1452283_at	BC004678	71323	Ras association (RalGDS/AF-6) domain family 8	Rassf8	5.62	1.89E-08	2.92E-06	10.10	8.87	1.25	2.38	
1077	1452314_at	BB827235	16551	kinesin family member 11	Kif11	-4.21	2.57E-05	1.53E-03	3.88	4.96	-1.09	-2.12	
1078	1452327_at	BF164393	232227	IQ motif and Sec7 domain 1	Iqsec1	4.50	6.72E-06	4.90E-04	7.97	6.82	1.15	2.21	
1079	1452352_at	BG064656	13025	cytotoxic T lymphocyte-associated protein 2 beta	Ctla2b	-6.89	5.50E-12	1.80E-09	10.60	12.20	-1.61	-3.06	
1080	1452353_at	BB762731	68526	G protein-coupled receptor 155	Gpr155	-5.73	1.02E-08	1.65E-06	7.05	8.47	-1.42	-2.67	
1081	1452406_x_at	AJ007909	170942	erythroid differentiation regulator 1	Erdr1	4.58	4.62E-06	3.58E-04	9.16	8.12	1.04	2.05	
1082	1452408_at	AF140709	436440	G protein-coupled receptor 31, D17Leh66c region	Gpr31c	-3.60	3.14E-04	1.19E-02	7.57	8.44	-0.87	-1.82	
1083	1452440_at	BI730298	100048347 /// 21944 /// 619441	tumor necrosis factor (ligand) superfamily, member 12 /// tumor necrosis factor (ligand) superfamily, member 12-member 13 /// similar to TWEAK	LOC100048347 /// Tnfsf12 /// Tnfsf12-tnfsf13	3.83	1.28E-04	5.84E-03	10.80	9.91	0.86	1.82	
1084	1452458_s_at	BC022648	69706	peptidylprolyl isomerase (cyclophilin) like 5	Ppil5	-11.41	3.95E-30	3.42E-27	1.90	2.91	-1.01	-2.02	
1085	1452521_a_at	X62701	18793	plasminogen activator, urokinase receptor	Plaur	7.64	2.09E-14	1.01E-11	10.60	8.87	1.71	3.27	
1086	1452534_a_at	X67668	97165	high mobility group box 2	Hmgb2	-3.27	1.08E-03	3.14E-02	8.33	9.08	-0.75	-1.68	
1087	1452540_a_at	M25487	100046213 /// 319179 /// 319185 /// 319186 /// 319188 /// 319189 ///	histone cluster 1, H2bc /// histone cluster 1, H2be /// histone cluster 1, H2bl /// histone cluster 1, H2bm /// histone cluster 1, H2bp /// histone cluster 2, H2bb /// H2b histone family, member	Hist1h2bc /// Hist1h2be /// Hist1h2bl /// Hist1h2bm /// Hist1h2bp /// Hist2h2bb ///	-3.72	2.00E-04	8.30E-03	9.53	10.40	-0.83	-1.78	
1088	1452608_at	BB046347	56309	c-myc binding protein	Mycbp	-3.61	3.02E-04	1.15E-02	5.02	6.05	-1.03	-2.05	
1089	1452619_a_at	AI463408	76223	ATP/GTP binding protein-like 3	Agbl3	-3.45	5.64E-04	1.89E-02	3.79	4.65	-0.85	-1.81	
1090	1452639_at	BG920295	224794	ectonucleotide pyrophosphatase/phosphodiesterase 4	Enpp4	-3.29	1.01E-03	2.98E-02	7.33	8.14	-0.81	-1.75	
1091	1452657_at	AK005223	108012	adaptor-related protein complex 1, sigma 2 subunit	Ap1s2	-3.55	3.87E-04	1.40E-02	8.69	9.50	-0.80	-1.75	
1092	1452700_s_at	AK003597	211255	kelch repeat and BTB (POZ) domain containing 7	Kbtbd7	-3.28	1.02E-03	3.01E-02	8.14	8.90	-0.76	-1.69	
1093	1452769_at	AK014408	74315	ring finger protein 145	Rnf145	-3.51	4.56E-04	1.59E-02	9.42	10.20	-0.78	-1.72	RIKEN cDNA 3732413I11 gene (RING finger protein 145)
1094	1452771_s_at	AK012088	74205	acyl-CoA synthetase long-chain family member 3	Acsl3	4.22	2.47E-05	1.48E-03	8.00	6.94	1.07	2.09	
1095	1452830_s_at	AK010453	69719	carbamoyl-phosphate synthetase 2, aspartate transcarbamylase, and dihydroorotase	Cad	-4.67	3.05E-06	2.53E-04	4.06	5.31	-1.25	-2.38	
1096	1452841_at	BG073164	70974	phosphoglucomutase 2-like 1	Pgm2l1	-3.43	6.02E-04	1.98E-02	6.72	7.61	-0.89	-1.85	
1097	1452903_at	AK020090	68176	RIKEN cDNA 6230427J02 gene	6230427J02Rik	-3.33	8.78E-04	2.68E-02	6.63	7.50	-0.87	-1.83	RIKEN cDNA 6230427J02 gene (ubiquitin-activating enzyme E1-like)
1098	1452911_at	AK017680	114715	sprouty protein with EVH-1 domain 1, related sequence	Spred1	-4.47	7.72E-06	5.55E-04	8.12	9.16	-1.04	-2.05	
1099	1452942_at	BF383782	74868	transmembrane protein 65	Tmem65	4.31	1.64E-05	1.04E-03	10.70	9.75	0.97	1.95	RIKEN cDNA 4930438D12 gene (Transmembrane protein 65)
1100	1452954_at	AV162459	68612	ubiquitin-conjugating enzyme E2C	Ube2c	-3.40	6.65E-04	2.15E-02	6.06	6.99	-0.94	-1.91	
1101	1452956_a_at	BI655075	52668	DNA segment, Chr 12, ERATO Doi 647, expressed	D12Ertd647e	4.28	1.83E-05	1.15E-03	8.64	7.63	1.01	2.01	
1102	1452992_at	AK008199	668450	predicted gene, EG668450	EG668450	-3.46	5.37E-04	1.82E-02	4.77	5.75	-0.98	-1.98	
1103	1453006_at	AI506568	72514	fibroblast growth factor binding protein 3	Fgfbp3	7.74	9.99E-15	5.11E-12	7.31	5.19	2.12	4.35	RIKEN cDNA 2610306H15 gene (Fibroblast growth factor binding protein 3)
1104	1453021_at	BM899291	78808	syntaxin binding protein 5 (tomosyn)	Stxbp5	3.51	4.47E-04	1.57E-02	8.33	7.48	0.84	1.79	
1105	1453072_at	BQ265798	71862	G protein-coupled receptor 160	Gpr160	-3.39	7.05E-04	2.24E-02	6.27	7.19	-0.92	-1.89	
1106	1453074_at	AK004912	68440	dual specificity phosphatase 23	Dusp23	3.71	2.08E-04	8.58E-03	3.63	3.00	0.63	1.54	
1107	1453119_at	BB530087	71198	OTU domain containing 1	Otud1	-4.61	4.00E-06	3.15E-04	6.98	8.13	-1.15	-2.23	
1108	1453152_at	AK004794	71738	MAM domain containing 2	Mamdc2	10.36	0.00E+00	0.00E+00	5.82	3.38	2.43	5.39	
1109	1453224_at	AW049828	22682	zinc finger, AN1-type domain 5	Zfand5	3.21	1.32E-03	3.63E-02	7.13	6.25	0.88	1.84	
1110	1453228_at	AK017897	74732	syntaxin 11	Stx11	3.88	1.03E-04	4.85E-03	7.89	6.90	0.99	1.98	
1111	1453234_at	AV222559	74152	RIKEN cDNA 1300002K09 gene	1300002K09Rik	4.25	2.09E-05	1.30E-03	8.24	7.20	1.04	2.06	RIKEN cDNA 1300002K09 gene (unknown)
1112	1453286_at	BB085537	18845	plexin A2	Plxna2	3.26	1.13E-03	3.22E-02	5.80	4.87	0.93	1.90	
1113	1453313_at	AK017464	75747	sestrin 3	Sesn3	-3.42	6.24E-04	2.05E-02	4.13	5.05	-0.91	-1.88	
1114	1453384_at	AK014551	329260	DENN/MADD domain containing 1B	Dennd1b	-3.51	4.45E-04	1.56E-02	3.63	4.46	-0.83	-1.78	RIKEN cDNA 4632404N19 gene (unknown)
1115	1453386_at	AK008612	69136	tumor suppressor candidate 1	Tusc1	5.45	5.05E-08	6.83E-06	10.00	8.80	1.21	2.31	
1116	1453472_a_at	AK016183	75345	SLAM family member 7	Slamf7	3.64	2.73E-04	1.06E-02	10.40	9.58	0.81	1.76	
1117	1453524_at	BB548904	71360	RIKEN cDNA 5530401D11 gene	5530401D11Rik	4.97	6.54E-07	6.49E-05	3.87	2.97	0.90	1.86	
1118	1453596_at	AK013239	15902	inhibitor of DNA binding 2	Id2	4.16	3.12E-05	1.79E-03	5.63	4.48	1.15	2.22	
1119	1453730_at	AK015593	67630	sterile alpha motif domain containing 8	Samd8	4.57	4.80E-06	3.69E-04	5.86	4.58	1.28	2.42	
1120	1453783_at	BE864772	100045958	similar to hCG45299	LOC100045958	-3.98	6.87E-05	3.51E-03	4.55	5.66	-1.12	-2.17	
1121	1453836_a_at	AK006949	23945	monoglyceride lipase	Mgll	5.57	2.48E-08	3.72E-06	5.95	4.42	1.53	2.89	
1122	1453851_a_at	AK007410	23882	growth arrest and DNA-damage-inducible 45 gamma	Gadd45g	7.70	1.38E-14	6.89E-12	8.59	6.66	1.94	3.83	
1123	1454069_at	AK018495	74536	RIKEN cDNA 9030409C19 gene	9030409C19Rik	-3.97	7.21E-05	3.64E-03	2.99	3.68	-0.69	-1.61	RIKEN cDNA 9030409C19 gene (unknown)
1124	1454617_at	BG072824	105171	arrestin domain containing 3	Arrdc3	-4.20	2.64E-05	1.56E-03	9.73	10.70	-0.95	-1.93	
1125	1454620_x_at	AV213405	100043734 /// 20104 /// 236932 /// 434404 /// 545640 /// 624159 ///	ribosomal protein S6 /// similar to 40S ribosomal protein S6 /// predicted gene, EG434404 /// similar to Ribosomal protein S6 /// predicted gene, EG629575 /// predicted gene, EG666577	EG434404 /// EG629575 /// EG666577 /// EG667717 /// LOC100043734 /// LOC236932 ///	-4.66	3.11E-06	2.57E-04	15.00	15.30	-0.36	-1.28	
1126	1454631_at	BI689897	83602	general transcription factor II A, 1	Gtf2a1	4.55	5.24E-06	3.99E-04	10.40	9.36	1.01	2.02	RIKEN cDNA 6330549H03 gene (Transcription initiation factor IIA subunit 1)
1127	1454632_at	AV328515	268567	RIKEN cDNA 6330442E10 gene	6330442E10Rik	-3.48	5.11E-04	1.74E-02	9.59	10.40	-0.78	-1.72	RIKEN cDNA 6330442E10 gene (Uncharacterized protein C14orf83 homolog)
1128	1454656_at	AV271736	219140	spermatogenesis associated 13	Spata13	-5.33	9.62E-08	1.22E-05	4.33	5.79	-1.47	-2.76	
1129	1454663_at	BQ176989	100047658 /// 217869	eukaryotic translation initiation factor 5 /// similar to Eukaryotic translation initiation factor 5	Eif5 /// LOC100047658	3.18	1.47E-03	3.96E-02	10.20	9.47	0.71	1.63	
1130	1454664_a_at	BQ176989	100047658 /// 217869	eukaryotic translation initiation factor 5 /// similar to Eukaryotic translation initiation factor 5	Eif5 /// LOC100047658	4.12	3.86E-05	2.14E-03	12.00	11.00	0.94	1.92	
1131	1454671_at	BB005488	231070	insulin induced gene 1	Insig1	6.25	4.07E-10	9.17E-08	9.95	8.56	1.39	2.62	
1132	1454694_a_at	BM211413	21973	topoisomerase (DNA) II alpha	Top2a	-3.90	9.73E-05	4.66E-03	5.83	6.92	-1.08	-2.12	
1133	1454711_at	BB080177	223435	triple functional domain (PTPRF interacting)	Trio	3.23	1.24E-03	3.48E-02	7.96	7.16	0.80	1.75	
1134	1454737_at	AV295798	75590	dual specificity phosphatase 9	Dusp9	4.91	8.94E-07	8.53E-05	4.89	3.70	1.19	2.28	
1135	1454757_s_at	AW554405	52668	DNA segment, Chr 12, ERATO Doi 647, expressed	D12Ertd647e	3.72	1.99E-04	8.30E-03	8.90	8.05	0.85	1.80	
1136	1454777_at	BB553107	101488	solute carrier organic anion transporter family, member 2b1	Slco2b1	-6.21	5.41E-10	1.19E-07	6.81	8.37	-1.56	-2.95	
1137	1454809_at	BE686893	211329	nuclear receptor coactivator 7	Ncoa7	-3.81	1.38E-04	6.18E-03	8.78	9.65	-0.86	-1.82	
1138	1454816_at	BB431808	19889	retinitis pigmentosa 2 homolog (human)	Rp2h	-3.25	1.17E-03	3.31E-02	7.77	8.54	-0.77	-1.71	
1139	1454847_at	BG917242	218454	lipoma HMGIC fusion partner-like 2	Lhfpl2	5.86	4.76E-09	8.31E-07	10.70	9.35	1.31	2.48	
1140	1454858_x_at	AV171622	70152	methyltransferase like 7A	Mettl7a	4.58	4.70E-06	3.63E-04	9.16	8.13	1.04	2.05	RIKEN cDNA 3300001H21 gene (methyltransferase like 7A)
1141	1454880_s_at	BB212341	171543	Bcl2 modifying factor	Bmf	-5.84	5.33E-09	9.23E-07	5.42	7.03	-1.61	-3.05	
1142	1454896_at	BM235554	19664	recombination signal binding protein for immunoglobulin kappa J region	Rbpj	-3.41	6.56E-04	2.13E-02	10.10	10.90	-0.77	-1.71	
1143	1454933_at	BI409701	230752	RIKEN cDNA 2610027C15 gene	2610027C15Rik	-4.48	7.50E-06	5.43E-04	7.35	8.44	-1.09	-2.13	RIKEN cDNA 2610027C15 gene (Transmembrane protein C1orf78 homolog)
1144	1454939_at	BB268102	239510	Transcribed locus	NA	-3.09	2.00E-03	5.00E-02	4.08	4.90	-0.81	-1.76	
1145	1454942_at	AW554191	63913	niban protein	Niban	-4.19	2.82E-05	1.65E-03	7.75	8.74	-0.99	-1.99	
1146	1454966_at	BQ175493	241226	integrin alpha 8	Itga8	-4.92	8.84E-07	8.47E-05	3.12	4.12	-1.00	-2.00	
1147	1454984_at	AV246615	106020	expressed sequence AW061234	AW061234	-5.12	3.07E-07	3.33E-05	4.67	6.10	-1.43	-2.70	RIKEN cDNA A230075M04 gene (unknown)

	A	B	C	D	E	F	G	H	I	J	K	L	M
1148	1455011_at	BG089858	170459	StAR-related lipid transfer (START) domain containing 4	Stard4	3.67	2.46E-04	9.85E-03	7.41	6.42	0.98	1.97	
1149	1455015_at	BE853276	71310	TBC1 domain family, member 9	Tbc1d9	3.94	8.01E-05	3.98E-03	10.00	9.16	0.88	1.83	RIKEN cDNA 4933431N12 gene (TBC1 domain family member 9)
1150	1455033_at	BB325849	329739	RIKEN cDNA B430201A12 gene	B430201A12Rik	-3.10	1.93E-03	4.88E-02	8.64	9.34	-0.70	-1.63	RIKEN cDNA B430201A12 gene (Protein FAM102B)
1151	1455078_at	BG075662	237860	slingshot homolog 2 (Drosophila)	Ssh2	-5.52	3.48E-08	5.01E-06	6.34	7.78	-1.44	-2.72	1455078_at (Unknown)
1152	1455161_at	BB202185	229694	expressed sequence AI504432	AI504432	-5.14	2.79E-07	3.09E-05	3.69	4.98	-1.29	-2.45	expressed sequence AI504432
1153	1455294_at	BB765875	68722	RIKEN cDNA 1110029L17 gene	1110029L17Rik	3.79	1.54E-04	6.69E-03	8.26	7.35	0.92	1.89	RIKEN cDNA 1110029L17 gene (unknown)
1154	1455324_at	BQ176176	433022	phosphatidylinositol-specific phospholipase C, X domain containing 2	Plcxd2	7.88	3.33E-15	1.90E-12	7.51	5.37	2.15	4.43	1455324_at (Unknown)
1155	1455389_s_at	AI415636	108745	RIKEN cDNA 2310051F07 gene	2310051F07Rik	4.92	8.63E-07	8.31E-05	10.30	9.21	1.09	2.14	
1156	1455477_s_at	AA396586	67182	PDZK1 interacting protein 1	Pdzk1ip1	3.94	8.14E-05	4.04E-03	9.10	8.21	0.89	1.86	
1157	1455550_x_at	BE691746	26943	serine incorporator 3	Serinc3	-3.48	5.01E-04	1.72E-02	13.90	14.40	-0.49	-1.40	
1158	1455577_at	BE196980	56838	chemokine (C-C motif) ligand 28	Ccl28	3.77	1.66E-04	7.13E-03	2.91	2.50	0.41	1.33	
1159	1455592_at	BM211048	93840	vang-like 2 (van gogh, Drosophila)	Vangl2	-5.21	1.86E-07	2.18E-05	2.51	3.15	-0.64	-1.56	
1160	1455618_x_at	BI440178	232670	tetraspanin 33	Tspan33	-3.90	9.59E-05	4.61E-03	6.14	7.20	-1.06	-2.08	
1161	1455646_at	BI904583	76511	RIKEN cDNA 2010004M13 gene	2010004M13Rik	-4.42	1.00E-05	6.92E-04	4.14	5.33	-1.19	-2.28	RIKEN cDNA 2010004M13 gene (unknown)
1162	1455661_at	BB211563	791294	predicted gene, ENSMUSG00000054945	ENSMUSG00000054945	-3.67	2.46E-04	9.85E-03	2.86	3.41	-0.56	-1.47	1455661_at (Unknown)
1163	1455731_at	BI158381	71279	solute carrier family 29 (nucleoside transporters), member 3	Slc29a3	3.35	8.08E-04	2.50E-02	11.30	10.50	0.77	1.70	
1164	1455795_at	BM207218	212898	dermatan sulfate epimerase	Dse	-3.11	1.85E-03	4.72E-02	10.40	11.20	-0.72	-1.64	
1165	1455796_x_at	BB549310	56177	olfactomedin 1	Olfm1	-3.62	2.94E-04	1.13E-02	5.57	6.59	-1.02	-2.03	
1166	1455870_at	AI649048	11641	A kinase (PRKA) anchor protein 2	Akap2	7.03	2.07E-12	7.58E-10	6.34	4.44	1.91	3.75	
1167	1455885_at	AV238106	231842	RIKEN cDNA 6530401C20 gene	6530401C20Rik	8.78	0.00E+00	0.00E+00	8.04	5.70	2.34	5.06	RIKEN cDNA 6530401C20 gene (Archaemetzincin-1 (EC 3.-.-.-) (Archeobacterial metalloproteinase-like protein 1).)
1168	1455956_x_at	AV310588	12444	cyclin D2	Ccnd2	-7.19	6.69E-13	2.58E-10	7.23	8.98	-1.75	-3.37	
1169	1455982_at	BF466891	194952	jumonji domain containing 4	Jmjd4	3.37	7.49E-04	2.36E-02	6.32	5.35	0.97	1.96	
1170	1455995_at	BB125269	215821	DNA segment, Chr 10, Brigham & Women's Genetics 1379 expressed	D10Bwg1379e	6.27	3.72E-10	8.46E-08	7.56	5.85	1.70	3.25	
1171	1456022_at	BB554636	15258	homeodomain interacting protein kinase 2	Hipk2	4.63	3.74E-06	2.97E-04	9.14	8.09	1.05	2.07	
1172	1456064_at	AI323624	229694	expressed sequence AI504432	AI504432	-3.63	2.81E-04	1.09E-02	4.72	5.75	-1.03	-2.04	expressed sequence AI504432 (Potassium voltage-gated channel subfamily A member 3, MK3)
1173	1456116_at	BB431091	100045979 /// 18163	catenin (cadherin associated protein), delta 2 /// similar to arm-repeat protein NPRAP/neurojungin	Ctnnd2 /// LOC100045979	-3.19	1.43E-03	3.87E-02	4.61	5.51	-0.90	-1.86	
1174	1456126_at	BB485735	240354	mucosa associated lymphoid tissue lymphoma translocation gene 1	Malt1	3.98	6.80E-05	3.48E-03	8.56	7.62	0.94	1.92	
1175	1456153_at	BB038915	237860	slingshot homolog 2 (Drosophila)	Ssh2	-5.75	8.94E-09	1.46E-06	6.81	8.26	-1.45	-2.73	
1176	1456163_at	AV127670	108900	RIKEN cDNA 2700049P18 gene	2700049P18Rik	-4.22	2.44E-05	1.47E-03	6.07	7.22	-1.15	-2.21	RIKEN cDNA 2700049P18 gene (unknown)
1177	1456174_x_at	AV309418	17988	N-myc downstream regulated gene 1	Ndrg1	4.29	1.79E-05	1.13E-03	11.80	10.90	0.98	1.98	
1178	1456195_x_at	BB543979	16419	integrin beta 5	Itgb5	-3.56	3.74E-04	1.36E-02	9.77	10.60	-0.80	-1.74	
1179	1456204_at	AV069567	66487	small nucleolar RNA host gene (non-protein coding) 8	Snhg8	-3.78	1.58E-04	6.83E-03	10.30	11.10	-0.87	-1.82	RIKEN cDNA 2010107H07 gene (unknown)
1180	1456209_x_at	BB076850	50794	Adult male hypothalamus cDNA, RIKEN full-length enriched library, clone:A230066G06 product:unclassifiable, full insert sequence	NA	-5.68	1.32E-08	2.11E-06	2.56	3.33	-0.78	-1.71	
1181	1456214_at	BB197591	54216	protocadherin 7	Pcdh7	-3.18	1.49E-03	4.00E-02	7.71	8.47	-0.76	-1.69	
1182	1456250_x_at	BB533460	21810	transforming growth factor, beta induced	Tgfbi	-8.44	3.08E-17	2.20E-14	9.22	11.20	-1.94	-3.85	
1183	1456280_at	BG067086	269582	claspin homolog (Xenopus laevis)	Clspn	-3.91	9.42E-05	4.55E-03	4.21	5.27	-1.06	-2.09	
1184	1456292_a_at	AV147875	22352	vimentin	Vim	3.87	1.11E-04	5.17E-03	14.40	13.80	0.59	1.51	
1185	1456312_x_at	AV224521	227753	gelsolin	Gsn	-4.61	3.99E-06	3.15E-04	12.30	13.30	-0.96	-1.95	
1186	1456324_at	AV127111	212276	zinc finger protein 748	Zfp748	-3.13	1.75E-03	4.54E-02	5.26	6.16	-0.90	-1.86	RIKEN cDNA 2610014M12 gene (zinc finger protein 748 isoform 2)
1187	1456341_a_at	AV354744	16601 /// 70273	Kruppel-like factor 9 /// RIKEN cDNA 2310051E17 gene	2310051E17Rik /// Klf9	3.66	2.49E-04	9.94E-03	9.55	8.74	0.82	1.76	
1188	1456377_x_at	AV010467	632329 /// 67803	LIM domain containing 2 /// similar to epithelial protein lost in neoplasm	Limd2 /// LOC632329	-3.12	1.82E-03	4.65E-02	11.30	12.00	-0.74	-1.67	RIKEN cDNA 0610025L06 gene (LIM domain-containing protein 2)
1189	1456393_at	AI642124	66360	RIKEN cDNA 2310002J21 gene	2310002J21Rik	-3.21	1.33E-03	3.65E-02	7.86	8.62	-0.76	-1.69	
1190	1456439_x_at	BB209438	171580	microtubule associated monoxygenase, calponin and LIM domain containing 1	Mical1	3.87	1.10E-04	5.16E-03	8.91	8.03	0.88	1.85	
1191	1456498_at	BB284583	16401	integrin alpha 4	Itga4	-4.09	4.34E-05	2.37E-03	6.85	7.89	-1.04	-2.06	1456498_at (Integrin alpha-4 precursor (Integrin alpha-IV) (VLA-4), CD49d, Itga4)
1192	1456510_x_at	BB703414	393082	UbiE-YGHL1 fusion protein	Ubie	4.05	5.13E-05	2.74E-03	8.00	6.98	1.02	2.03	
1193	1456543_at	BB037474	58182	prokineticin receptor 1	Prokr1	5.39	6.89E-08	9.00E-06	4.86	3.58	1.28	2.43	
1194	1456573_x_at	BB205930	18115	nicotinamide nucleotide transhydrogenase	Nnt	-3.61	3.09E-04	1.17E-02	8.58	9.40	-0.82	-1.77	
1195	1456574_at	BB224546	627049	zinc finger protein 800	Zfp800	-4.19	2.84E-05	1.65E-03	7.57	8.57	-1.00	-2.01	
1196	1456735_x_at	BB458645	235534	acid phosphatase-like 2	Acpl2	-3.21	1.33E-03	3.66E-02	6.25	7.13	-0.87	-1.83	
1197	1456753_at	AW209075	NA	NA	NA	4.80	1.59E-06	1.42E-04	7.58	6.30	1.28	2.43	1456753_at (Forkhead box protein O4 (Fork head domain transcription factor AFX1) (Afxh))
1198	1456763_at	BB365879	26936	expressed sequence AA536749	AA536749	9.64	0.00E+00	0.00E+00	1.91	1.80	0.11	1.08	expressed sequence AA536749
1199	1456789_at	AW491540	242466	zinc finger protein 462	Zfp462	8.65	0.00E+00	0.00E+00	5.18	3.22	1.96	3.89	
1200	1456792_at	BG967046	100045317 /// 381605	TBC1 domain family, member 2 /// similar to TBC1 domain family, member 2	LOC100045317 /// Tbc1d2	5.32	1.02E-07	1.28E-05	8.81	7.56	1.25	2.38	
1201	1456874_at	BB817332	399558	fibronectin leucine rich transmembrane protein 2	Flrt2	3.47	5.26E-04	1.78E-02	9.10	8.32	0.78	1.72	1456874_at (Fibronectin leucine rich transmembrane protein 2)
1202	1456887_at	AW228687	14747	chemokine-like receptor 1	Cmklr1	-4.78	1.78E-06	1.57E-04	7.62	8.76	-1.14	-2.20	
1203	1456898_at	AI426862	100045958	similar to hCG45299	LOC100045958	-3.26	1.12E-03	3.21E-02	7.42	8.22	-0.79	-1.73	
1204	1457028_at	BM226884	243308	RIKEN cDNA A430033K04 gene	A430033K04Rik	-4.33	1.50E-05	9.72E-04	3.10	3.93	-0.83	-1.78	RIKEN cDNA A430033K04 gene (unknown)
1205	1457067_at	BB535696	20479	vacuolar protein sorting 4b (yeast)	Vps4b	-4.30	1.73E-05	1.09E-03	2.79	3.44	-0.65	-1.57	1457067_at(Vacuolar protein sorting-associating protein 4B (Suppressor of K(+) transport growth defect 1) (Protein SKD1))
1206	1457248_x_at	BB554029	15490	hydroxysteroid (17-beta) dehydrogenase 7	Hsd17b7	5.30	1.15E-07	1.41E-05	7.38	5.93	1.45	2.73	
1207	1457254_x_at	BB302103	268567	RIKEN cDNA 6330442E10 gene	6330442E10Rik	-4.26	2.03E-05	1.26E-03	8.53	9.50	-0.97	-1.96	RIKEN cDNA 6330442E10 gene (Uncharacterized protein C14orf83 homolog)
1208	1457268_at	BM199751	208266	DOT1-like, histone H3 methyltransferase (S. cerevisiae)	Dot1l	3.12	1.84E-03	4.69E-02	7.76	6.96	0.79	1.74	
1209	1457293_at	BF714580	11443	cholinergic receptor, nicotinic, beta polypeptide 1 (muscle)	NA	-3.10	1.94E-03	4.89E-02	4.66	5.53	-0.88	-1.84	
1210	1457322_at	BM938277	NA	Transcribed locus	NA	-4.47	7.82E-06	5.61E-04	6.90	8.03	-1.13	-2.18	1457322_at (Complement C1q subcomponent subunit C precursor, C1qc)
1211	1457359_at	BB540672	234515	inositol polyphosphate-4-phosphatase, type II	Inpp4b	-3.47	5.25E-04	1.78E-02	4.04	4.96	-0.91	-1.88	
1212	1457404_at	BM240058	80859	Nuclear factor of kappa light polypeptide gene enhancer in B-cells inhibitor, zeta	Nfkbiz	-3.36	7.86E-04	2.45E-02	4.61	5.56	-0.95	-1.93	
1213	1457424_at	BB760085	14048	eyes absent 1 homolog (Drosophila)	Eya1	3.14	1.66E-03	4.36E-02	6.97	6.09	0.87	1.83	
1214	1457633_x_at	AV005759	12862	cytochrome c oxidase, subunit VI a, polypeptide 2	Cox6a2	-9.89	4.45E-23	3.58E-20	1.89	2.49	-0.59	-1.51	
1215	1457644_s_at	BB554288	14825	chemokine (C-X-C motif) ligand 1	Cxcl1	3.94	8.22E-05	4.07E-03	5.63	4.54	1.10	2.14	
1216	1457691_at	BB525541	321019	Epstein-Barr virus induced gene 2	Ebi2	-4.63	3.66E-06	2.93E-04	6.41	7.63	-1.22	-2.32	1457691_at (EBV-induced G-protein coupled receptor 2 homolog (EBI2))
1217	1457780_at	BB767243	74732	syntaxin 11	Stx11	3.63	2.85E-04	1.10E-02	5.27	4.29	0.98	1.97	1457780_at (Syntaxin-11)
1218	1458019_at	BB548602	NA	2 days pregnant adult female oviduct cDNA, RIKEN full-length enriched library, clone:E230011B21 product:unclassifiable, full insert sequence	NA	-7.63	2.42E-14	1.16E-11	3.34	5.19	-1.85	-3.59	1458019_at (macrophage scavenger receptor 2)
1219	1458299_s_at	BB820441	18037	nuclear factor of kappa light polypeptide gene enhancer in B-cells inhibitor, epsilon	Nfkbie	-5.43	5.65E-08	7.55E-06	7.90	9.18	-1.27	-2.42	
1220	1458408_at	BB160137	67630	sterile alpha motif domain containing 8	Samd8	5.37	8.08E-08	1.04E-05	8.39	7.07	1.32	2.50	
1221	1458459_a_at	BB312740	319711	RIKEN cDNA E230029C05 gene	E230029C05Rik	-4.39	1.14E-05	7.76E-04	4.13	5.31	-1.18	-2.27	expressed sequence AI452102 (unknown)

	A	B	C	D	E	F	G	H	I	J	K	L	M
1222	1458551_at	BM239547	791388	predicted gene, ENSMUSG00000069163	ENSMUSG00000069163	-4.63	3.59E-06	2.90E-04	4.06	5.30	-1.24	-2.36	
1223	1459253_at	AW556597	105171	Arrestin domain containing 3	Arrdc3	-5.93	2.99E-09	5.51E-07	2.73	3.72	-0.99	-1.99	
1224	1459350_at	BB204648	54607	suppressor of cytokine signaling 6	NA	3.85	1.16E-04	5.35E-03	3.27	2.74	0.53	1.45	
1225	1459351_at	BB188444	11549	adrenergic receptor, alpha 1a	Adra1a	3.68	2.34E-04	9.47E-03	7.19	6.18	1.01	2.01	
1226	1459386_at	AV319015	236193	zinc finger protein 709	Zfp709	5.61	2.07E-08	3.16E-06	5.10	3.72	1.38	2.60	cDNA sequence BC021921 (Zinc finger protein 14)
1227	1459722_at	AI427602	67263	Transcribed locus	NA	-3.49	4.88E-04	1.69E-02	4.62	5.60	-0.98	-1.98	
1228	1459728_at	AI553531	57905	ISY1 splicing factor homolog (S. cerevisiae)	Isy1	-3.81	1.40E-04	6.25E-03	4.26	5.30	-1.04	-2.06	RIKEN cDNA 5830446M03 gene (unknown)
1229	1459742_at	BB800744	NA	NA	NA	-3.16	1.55E-03	4.12E-02	4.42	5.29	-0.88	-1.84	1459742_at (Unknown)
1230	1459861_s_at	AV221085	100039668 /// 30841	F-box and leucine-rich repeat protein 10 /// similar to F-box and leucine-rich repeat protein 10	Fbxl10 /// LOC100039668	-4.61	3.97E-06	3.13E-04	9.37	10.40	-1.04	-2.05	
1231	1459913_at	AI645293	22035	tumor necrosis factor (ligand) superfamily, member 10	Tnfsf10	-6.15	7.82E-10	1.66E-07	4.32	6.00	-1.68	-3.21	
1232	1460038_at	BG065255	18991	POU domain, class 3, transcription factor 1 /// similar to long overlapping ORF	LOC100045707 /// Pou3f1	3.44	5.92E-04	1.96E-02	9.11	8.34	0.77	1.71	
1233	1460064_at	BM237812	407802	cDNA sequence BC028789	BC028789	-4.19	2.78E-05	1.63E-03	2.87	3.55	-0.68	-1.60	
1234	1460082_at	BB298005	71777	inhibitor of growth family, member 3	Ing3	-6.91	4.88E-12	1.62E-09	2.50	3.51	-1.01	-2.02	
1235	1460109_at	AV253069	66855	Transcription factor 25 (basic helix-loop-helix)	Tcf25	3.82	1.36E-04	6.13E-03	3.36	2.80	0.56	1.47	
1236	1460110_at	BB376570	246102	rotatin	Rttn	3.81	1.37E-04	6.18E-03	3.43	2.85	0.58	1.50	
1237	1460116_s_at	AI450584	114715	sprouty protein with EVH-1 domain 1, related sequence	Spred1	-4.47	7.95E-06	5.68E-04	9.86	10.90	-1.01	-2.02	
1238	1460173_at	BC010840	16796	LIM and SH3 protein 1	Lasp1	3.52	4.27E-04	1.51E-02	12.30	11.50	0.81	1.75	
1239	1460196_at	NM_007620	12408	carbonyl reductase 1	Cbr1	3.55	3.86E-04	1.40E-02	9.17	8.37	0.80	1.74	
1240	1460283_at	NM_019453	54483	Mediterranean fever	Mefv	-3.13	1.78E-03	4.57E-02	5.01	5.91	-0.90	-1.86	
1241	1460378_a_at	BC010465	21753	testis derived transcript	Tes	4.48	7.51E-06	5.43E-04	8.67	7.62	1.05	2.08	
1242	1460381_at	BC023179	232855	cDNA sequence BC023179	BC023179	-3.15	1.65E-03	4.33E-02	4.46	5.33	-0.88	-1.83	cDNA sequence BC023179 (Unknown)
1243	1460465_at	BE632850	68169	RIKEN cDNA A930038C07 gene	A930038C07Rik	-3.48	5.08E-04	1.74E-02	4.06	4.98	-0.92	-1.89	RIKEN cDNA A930038C07 gene (Fibronectin type-III domain-containing protein C4orf31 homolog precursor)
1244	1460521_a_at	AK015312	109019	oligonucleotide/oligosaccharide-binding fold containing 2A	Obfc2a	4.62	3.79E-06	3.00E-04	8.19	7.04	1.15	2.22	RIKEN cDNA 5830411E10 gene (oligonucleotide/oligosaccharide-binding fold containing 2A)
1245	1460543_x_at	AV066985	19981	ribosomal protein L37a	Rpl37a	-4.23	2.37E-05	1.44E-03	14.40	14.80	-0.46	-1.38	
1246	1460554_s_at	BB795216	20340	golgi apparatus protein 1	Glg1	-3.12	1.81E-03	4.64E-02	7.22	7.99	-0.77	-1.71	
1247	1460586_at	BE573060	269878	multiple EGF-like-domains 8	Megf8	-3.24	1.18E-03	3.33E-02	4.52	5.43	-0.91	-1.88	
1248	1460650_at	U13836	11975	ATPase, H+ transporting, lysosomal V0 subunit A1	Atp6v0a1	3.72	2.00E-04	8.31E-03	8.66	7.79	0.87	1.82	
1249	1460651_at	AF036907	16797	linker for activation of T cells	Lat	3.95	7.92E-05	3.95E-03	6.25	5.12	1.13	2.19	

Table 2. List of differentially expressed genes between *L. amazonensis*-harboring MΦs and parasite-free MΦs.

Symbol	Name	Probe-set	LocusLink	Affymetrix (RTqPCR)	P-value
abcD2	ATP-binding cassette, sub-family D (ALD), member 2	1438431_at[a]	26874	-2.11	4.40e-03
acaca	acetyl-Coenzyme A carboxylase alpha	1427595_at	107476	-1.32	4.79e-03
acsl3	acyl-CoA synthetase long-chain family member 3	1452771_s_at	74205	+2.09	1.48e-03
adhfe1	alcohol dehydrogenase, iron containing, 1	1424393_s_at	76187	+1.61	4.40e-02
akr1a1	aldo-keto reductase family 1, member A1 (aldehyde reductase)	1430123_a_at	58810	+1.13	1.22e-03
aldoA	aldolase 1, A isoform	1433604_x_at[a]	11674	+1.72	1.28e-02
aldoC	aldolase 3, C isoform	1451461_a_at	11676	+1.89	1.13e-02
anxA1	annexin A1	1444016_at[a]	16952	+2.68	4.47e-05
apo2C	apolipoprotein C-II	1418069_at	11813	-1.63	4.57e-02
arg2	Arginase 2	1418847_at	11847	NM (+1.91)	NS
atf1	activating transcription factor 1	1417296_at	11908	+1.84	4.20e-03
atf3	activating transcription factor 3	1449363_at	11910	+1.77	1.09e-02
atp6V0a1	ATPase, H+ transporting, lysosomal V0 subunit a isoform 1	1460650_at[a]	11975	+1.82	8.31e-03
atp6V0c	ATPase, H+ transporting, V0 subunit C	1435732_x_at	11984	+1.27	5.48e-13
atp6V0d2	ATPase, H+ transporting, V0 subunit D, isoform 2	1444176_at[a]	24234	+2.32	1.12e-05
atp6V1a	ATPase, H+ transporting, V1 subunit A1	1422508_at	11964	+1.57	3.96e-02
atp6V1c1	ATPase, H+ transporting, V1 subunit C, isoform 1	1419546_at[a]	66335	+2.31	1.10e-05
atp6V1d	ATPase, H+ transporting, V1 subunit D	1416952_at[a]	73834	+1.82	6.97e-03

Symbol	Name	Probe-set	LocusLink	Affymetrix (RTqPCR)	P-value
atp6V1g1	ATPase, H+ transporting, V1 subunit G isoform 1	1423255_at[a]	66290	+1.84	3.78e-03
atp6V1h	ATPase, H+ transporting, lysosomal, V1 subunit H	1415826_at	108664	+1.69	2.39e-02
azin1	antizyme inhibitor 1	1422702_at	54375	+1.96	1.46e-03
brd8	bromodomain containing 8	1427193_at	78656	+1.08	3.75e-02
c1qa	complement component 1, q subcomponent, alpha polypeptide	1417381_at	12259	-1.48	3.15e-02
c1qb	complement component 1, q subcomponent, beta polypeptide	1417063_at	12260	-1.77	3.31e-04
c3	complement component 3	1423954_at	12266	-2.37	7.05e-06
c4b	complement component 4 (within H-2S)	1418021_at	12268	-1.76	4.55e-02
c5ar1	complement component 5a receptor 1	1439902_at	247623	-1.63	4.62e-02
ccr2	chemokine (C-C motif) receptor 2	1421187_at[a]	12772	-1.83 (-2.35)	6.42e-03
ccr3	chemokine (C-C motif) receptor 3	1422957_at	12771	-2.58 (-3.88)	2.49e-05
cd14	CD14 antigen	1417268_at	12475	-1.73	1.54e-03
cd200	CD200 antigen	1448788_at	17470	+4.14 (+6.52)	5.48e-13
cd274	CD274 antigen	1419714_at	60533	+1.93	1.61e-03
cd86	CD86 antigen	1420404_at[a]	12524	-1.83 (-1.03)	1.44e-02
cfh	complement component factor h	1450876_at	12628	-2.80	6.08e-06
c-fos	FBJ osteosarcoma oncogene	1423100_at	14281	-1.93	3.30e-03
ch25h	cholesterol 25-hydroxylase	1449227_at	12642	-6.57	1.39e-22
cmklr1	chemokine-like receptor 1	1456887_at	14747	-2.20	1.57e-04

Symbol	Name	Probe-set	LocusLink	Affymetrix (RTqPCR)	P-value
cx3cr1	chemokine (C-X3-C) receptor 1	1450020_at	13051	-2.65 (-5.26)	2.39e-05
cyp51	cytochrome P450, family 51	1450646_at[a]	13121	+2.78	2.10e-07
dhcr24	24-dehydrocholesterol reductase	1451895_a_at	74754	+3.17	2.69e-09
dio2	deiodinase, iodothyronine, type II	1418937_at[a]	13371	+25.92 (+41.03)	0.00e+00
eno2	enolase 2, gamma neuronal	1418829_a_at	13807	+2.60	6.08e-06
fabp3	fatty acid binding protein 3	1416023_at	14077	+2.29	5.58e-05
fabp4	fatty acid binding protein 4	1417023_a_at[a]	11770	+6.42	0.00e+00
fabp5	fatty acid binding protein 5	1416022_at[a]	16592	+1.57	4.70e-08
fbp1	fructose bisphosphatase 1	1448470_at	14121	-2.16	4.68e-03
fdft1	farnesyl diphosphate farnesyl transferase 1	1438322_x_at[a]	14137	+2.62	4.00e-06
fdps	farnesyl diphosphate synthetase	1423418_at	110196	+3.59	9.78e-12
h-2ma	histocompatibility 2, class II, locus DMa	1422527_at	14998	-1.88	3.00e-03
h60	histocompatibility 60	1439343_at	15101	-2.07	5.30e-09
hk2	hexokinase 2	1422612_at	15277	+1.75	1.09e-02
hk3	hexokinase 3	1435490_at	212032	+2.03	3.72e-04
hmgcr	3-hydroxy-3-methylglutaryl-Coenzyme A reductase	1427229_at	15357	+1.95	2.34e-03
hmgcs1	3-hydroxy-3-methylglutaryl-Coenzyme A synthase 1	1433446_at	208715	+2.48	1.07e-06
hsd17b7	hydroxysteroid (17-beta) dehydrogenase 7	1457248_x_at	15490	+2.73	1.41e-05
icam1	intercellular adhesion molecule	1424067_at	15894	-1.75	1.43e-02

Symbol	Name	Probe-set	LocusLink	Affymetrix (RTqPCR)	P-value
icam2	intercellular adhesion molecule 2	1448862_at	15896	-1.85	2.88e-02
idi1	isopentenyl-diphosphate delta isomerase	1451122_at[a]	319554	+2.72	2.77e-07
ifngr1	interferon gamma receptor 1	1448167_at	15979	-1.83 (-2.16)	4.66e-03
il10	interleukin 10	1450330_at	16153	-2.97 (-4.46)	1.11e-07
il10ra	interleukin 10 receptor, alpha	1448731_at	16154	-2.16 (-2.56)	4.40e-04
il11ra1	interleukin 11 receptor, alpha chain 1	1417505_s_at	16157	+2.24 (+3.55)	9.89e-05
il17rb	interleukin 17 receptor B	1420678_a_at	50905	-1.41	2.93e-02
il18	interleukin 18	1417932_at	16173	-1.77 (-2.12)	1.06e-02
il1b	interleukin 1 beta	1449399_a_at	16176	-3.09 (-5.17)	3.49e-07
il1rn	interleukin 1 receptor antagonist	1423017_a_at[a]	16181	+4.19 (+7.86)	0.00e+00
insig1	insulin induced gene 1	1454671_at	231070	+2.62	9.17e-08
itga4	integrin alpha 4	1456498_at[a]	16401	-2.06	2.37e-03
itgal	integrin alpha L	1435560_at	16408	-2.00	7.72e-03
klrk1	killer cell lectin-like receptor subfamily K, member 1	1450495_a_at	27007	-1.72	2.21e-02
ldhA	lactate dehydrogenase 1, A chain	1419737_a_at	16828	+1.79	2.71e-04
ldlr	low density lipoprotein receptor	1450383_at[a]	16835	+4.68	1.49e-13
lipe	lipase, hormone sensitive	1422820_at	16890	-2.20	2.90e-03
lpl	lipoprotein lipase	1431056_a_at	16956	-1.44	3.24e-02
lss	lanosterol synthase	1420013_s_at	16987	+2.05	2.29e-03

Symbol	Name	Probe-set	LocusLink	Affymetrix (RTqPCR)	P-value
maoa	monoamine oxidase A	1428667_at[a]	17161	+2.56	4.71e-06
mapk14	mitogen activated protein kinase 14 (p38 mapk)	1416703_at	26416	-1.61	4.97e-02
mgll	monoglyceride lipase	1426785_s_at	23945	+3.40	3.75e-08
mvd	mevalonate (diphospho) decarboxylase	1417303_at[a]	192156	+2.15	6.33e-04
ncoa4	nuclear receptor coactivator 4	1450006_at	27057	+1.65	3.15e-02
nfkbia	nuclear factor of kappa light chain gene enhancer in B-cells inhibitor, alpha	1448306_at	18035	-1.83	5.53e-03
nos2	nitric oxide synthase 2, inducible, macrophage	1420393_at	18126	NM (+1.28)	NS
odc1	Ornithine decarboxylase 1	1427364_a_at	18263	NM (+1.18)	NS
p4ha2	procollagen-proline, 2-oxoglutarate 4-dioxygenase (proline 4-hydroxylase), α II polypeptide	1417149_at	18452	+2.27	1.96e-03
pfkl	phosphofructokinase, liver, B-type	1439148_a_at	18641	+1.68	2.32e-02
pkg1	phosphoglycerate kinase 1	1417864_at	18655	+1.70	8.88e-03
pkm2	pyruvate kinase, muscle	1417308_at	18746	+1.51	4.57e-02
ppap2B	phosphatidic acid phosphatase type 2B	1448908_at[a]	67916	+8.53	0.00e+00
pros1	protein S (alpha)	1426246_at	19128	-2.09	2.66e-03
relb	avian reticuloendotheliosis viral (v-rel) oncogene related B	1417856_at	19698	-1.91	1.94e-02
sat1	spermidine/spermine N1-acetyl transferase 1	1420502_at	20229	+1.47	2.30e-02
sc4mol	sterol-C4-methyl oxidase-like	1423078_a_at	66234	+2.28	1.61e-05
sc5d	sterol-C5-desaturase (fungal ERG3, delta-5-desaturase) homolog (S. cerevisae)	1451457_at[a]	235293	+2.57	2.37e-06
scd1	stearoyl-Coenzyme A desaturase 1	1415964_at[a]	20249	+2.68	4.50e-05

Symbol	Name	Probe-set	LocusLink	Affymetrix (RTqPCR)	P-value
scd2	stearoyl-Coenzyme A desaturase 2	1415824_at[a]	20250	+2.45	1.32e-06
serping1	serine (or cysteine) peptidase inhibitor, clade G, member 1	1416625_at	12258	-1.35	4.84e-05
slc7a2	solute carrier family 7 (cationic amino acid transporter, y+ system), member 2	1436555_at[a]	11988	+4.14	6.07e-12
sms	spermine synthase	1434190_at[a]	20603	NM [-1.38[b]]	NS
socs6	suppressor of cytokine signaling 6	1450129_a_at	54607	+1.84	4.18e-03
sqle	squalene epoxidase	1415993_at	20775	+4.30	0.00e+00
srebf2	sterol regulatory element binding factor 2	1426744_at	20788	+1.84	1.26e-02
srm	spermidine synthase	1421260_a_at	20810	NM [-1.22 [b]]	NS
stard4	StAR-related lipid transfer (START) domain containing 4	1429239_a_at[a]	170459	+2.31	2.43e-04
tlr2	toll-like receptor 2	1419132_at	24088	-3.11 (-1.58)	1.83e-08
tlr7	toll-like receptor 7	1449640_at	170743	-1.77 (-1.07)	4.61e-02
tlr8	toll-like receptor 8	1450267_at	170744	-1.79	1.00e-02
tollip	toll interacting protein	1423048_a_at	54473	+1.69	3.57e-02

This table is an excerpt from the table of the 1,248 significantly modulated probe-sets, available as online supplementary material, and contains some genes tested by RTqPCR.

[a] when several probe-sets detect a target gene, data are only shown for the most modulated one. NM: No Modulation significantly detected with Affymetrix technology. NS: Not Significant *p*-value. [[b]] mean values obtained from the raw fluorescence intensities. Primer sequences for genes tested by RTqPCR are available upon request.
